# Benchmarking Cantilever Torque Magnetometry as a Platform for Characterizing Molecular Qubits: A Case Study on Ni(II) Complexes

**DOI:** 10.1021/jacs.6c00500

**Published:** 2026-03-05

**Authors:** Jett T. Janetzki, Arsen Raza, Matteo Briganti, Rocco Duquennoy, Anne-Laure Barra, Costanza Toninelli, Mauro Perfetti, Lorenzo Sorace

**Affiliations:** † Department of Chemistry “Ugo Schiff” and INSTM Research Unit, 9300 University of Florence, Via della Lastruccia, 13, Sesto Fiorentino 50019, Italy; ‡ European Laboratory for Non-Linear Spectroscopy (LENS), Via Nello Carrara 1, Sesto Fiorentino 50019, Italy; § National Institute of Optics (CNR-INO), Via Nello Carrara 1, Sesto Fiorentino 50019, Italy; ∥ Laboratoire National des Champs Magnétiques Intenses, CNRS, Université Grenoble Alpes, 25 Avenue des Martyrs, Grenoble 38042, France

## Abstract

Precise and experimentally accessible determination of the electronic structure of transition metal complexes remains a challenge in the development of molecular qubits, particularly for leading candidates with integer spin. Existing techniques often require large-scale facilities and substantial sample quantities or offer limited spectral access and sensitivity to subtle anisotropies. Here, we demonstrate that cantilever torque magnetometry (CTM) overcomes these limitations by combining high sensitivity to magnetic anisotropy with wide sample compatibility, minimal sample demands, and true laboratory-scale accessibility. By exploiting the distinct temperature dependences of *g*-tensor anisotropy and zero-field splitting (ZFS), CTM enables their experimental decoupling, yielding exceptionally precise bulk-mean value determination of spin Hamiltonian parameters from microgram-scale single crystals. The parameters extracted by CTM were found to be qualitatively consistent but quantitatively different from those determined using high-frequency electron paramagnetic resonance spectroscopy (∼1% for *g* and ∼5–15% for ZFS), highlighting that perfect agreement between magnetometric and resonance techniques is not guaranteed. Our study establishes CTM as a powerful and broadly accessible complement to magnetic resonance methods, opening new routes for high-precision characterization of low-anisotropy spin systems in molecular quantum information science.

## Graphical abstract

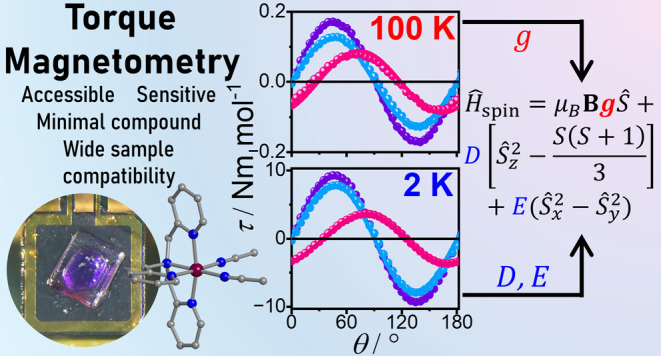

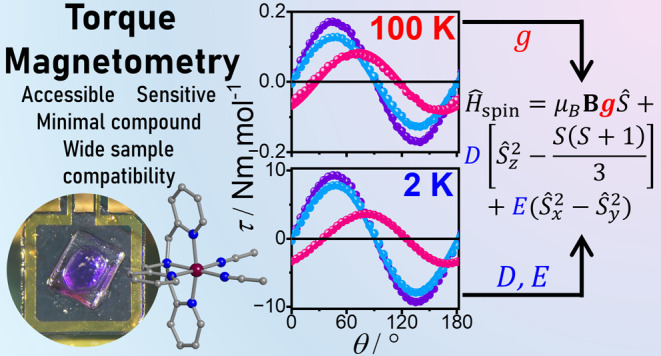

## Introduction

Quantum information science (QIS) is transforming digital technologies by harnessing quantum phenomena in computation, communication, sensing, and metrology.^1–4^ At the core of all quantum platforms is the realization of coherent and controllable qubits. Electron spin defects like nitrogen-vacancy centers in diamond offer optical initialization and single-qubit readout but suffer from limited synthetic tunability and scalability.^1,5,6^ On the other hand, a molecular approach offers atomically precise synthetic and electronic control unavailable in conventional solid-state systems.^2,3,7–11^ Groundbreaking preliminary results have yielded optically addressable molecular spin qubits with integer spins and small dc zero-field splitting (ZFS) based on Cr­(IV), V­(III), and Ni­(II), enabling coherent microwave control and efficient optical transitions.^7,12–14^ However, molecular qubits do face challenges regarding fast spin decoherence,^2,3,7,15^ sensitivity to local structure,^12,16–18^ and in the methods to initialize and readout.^2,3,7,12,19^ Sustained progress requires additional and complementary screening techniques to determine the electronic structure parameters governing qubit performance with high throughput and precision while remaining broadly accessible.

While each existing technique offers distinct advantages, all face intrinsic limitations that restrict their accessibility, applicability, or precision. Magnetic resonance techniques are crucial for probing spin coherence and addressing molecular qubits.^12–14,16,20–23^ However, in integer-spin systems, the ZFS often exceeds the microwave range of conventional electron paramagnetic resonance (EPR) spectrometers, leaving spectra partially or entirely unresolved.^24,25^ This typically necessitates high-frequency EPR (HF-EPR),^24^ which has proved extremely powerful for studying integer-spin molecules^24,26–31^ and molecular qubits.^13,14,16,21^ Single-crystal and/or multifrequency HF-EPR measurements can provide highly detailed spin Hamiltonian information, including tensor magnitudes, orientations, and correlations with molecular structure.^32–39^

Despite these strengths, the widespread application of HF-EPR is limited by practical considerations. Measurements are typically performed using specialized high-field instrumentation. While advanced laboratory-scale HF-EPR spectrometers can be constructed^40–42^ at costs comparable to commercial magnetometry platforms, such instruments are not commercially standardized and typically require specific technical expertise, custom development, and ongoing specialized maintenance. As a result, HF-EPR capabilities remain concentrated in a relatively small number of specialist laboratories, currently limiting broad accessibility and routine high-throughput use. As HF-EPR is most commonly performed on powder samples, measurements typically require tens of milligrams of material. In addition, while most HF-EPR studies are performed without dilution because line widths are typically dominated by inhomogeneous broadening, magnetic dilution into an isostructural diamagnetic host may be beneficial in cases where dipolar interactions contribute significantly to line broadening. Suitable diamagnetic hosts, however, are not always available. Complementary approaches to HF-EPR such as frequency-domain Fourier transform THz-EPR and far-infrared magnetic resonance directly probe ZFS^25,43,44^ but similarly depend on specialized sources and substantial sample quantities^25,44–49^ and are generally less sensitive to rhombicity (*E*/*D*) and *g*-anisotropy.^43,47,48^ By contrast, conventional powder magnetometry is widely accessible but cannot reliably quantify anisotropy due to orientation averaging and limited sensitivity,^24,50^ while single-crystal magnetometry cannot resolve anisotropy in systems containing noncollinear molecules.^50,51^ Collectively, these limitations leave a gap between molecular qubit design and experimentally accessible high-precision characterization.

Cantilever torque magnetometry (CTM) offers an underused yet powerful and practically accessible alternative that bridges the precision of HF-EPR and the availability of standard magnetometry.^50^ CTM is a thermodynamic technique that probes the ensemble-averaged magnetic response of a bulk sample and is highly sensitive to magnetic anisotropy. It is applicable to both integer- and half-integer-spin systems and can disentangle noncollinear contributions. Unlike EPR, CTM does not rely on linewidth resolution and does not benefit from magnetic dilution, providing a practical advantage for systems where dilution is impractical or ineffective. Critically, CTM’s instrumental accessibility is one of its key advantages: measurements require only microgram-scale single crystals and can be performed on widely available laboratory magnetometry platforms (e.g., Quantum Design Physical Property Measurement System (PPMS)) using a relatively low-cost cantilever insert. Because many laboratories focused on molecular magnetism and solid-state physics already possess compatible magnetometry systems, CTM can be readily implemented without new infrastructure. While CTM, like single-crystal EPR, requires well-oriented crystals, its minimal sample requirements at the microgram scale and laboratory-scale accessibility make it attractive for qubit analysis.

A further strength of CTM is its ability to deliver measurable torque signals across a broad temperature range, including ambient conditions. While HF-EPR can be performed above liquid N_2_ temperatures (77 K) in certain cases,^52–55^ routine measurements typically use cryogenic conditions to maximize signal intensity and mitigate line broadening.^13,16,21,26,56^ The broad temperature window of CTM allows to exploit the contrasting effects of the temperature dependences of *g-*anisotropy and ZFS, enabling these contributions to be disentangled within a single experiment.

Despite all of these advantages, CTM remains underutilized in molecular qubit studies. To highlight its capabilities, here, we apply CTM to weakly anisotropic integer-spin systems directly relevant to molecular qubits. We focus on octahedral Ni­(II) complexes (*S* = 1), which capture key features of integer-spin molecular qubits^13,16,21^ and whose ZFS commonly exceeds the frequency range of conventional commercial EPR spectrometers, thereby limiting routine spectroscopic analysis.^26–31,56–62^ While *S* = 2 complexes are also attractive, all currently optically addressable molecular qubits are *S* = 1.^3,12–14,23^ Although CTM studies on Ni­(II) systems remain scarce, prior work has demonstrated sensitivity to ZFS differences and magnetic-axis orientations;^63,64^ however, these studies relied on complementary single-crystal magnetometry or HF-EPR and focused on strongly exchanged systems or large ZFS values incompatible with qubit applications. Here, we select the six-coordinate octahedral complexes [Ni­(tpa)­(MeCN)_2_]­(OTf)_2_ (**1**)^65^ and [Ni­(tpa)­(phen)]­(PF_6_)_2_ (**2**) (tpa = tris­(2-pyridylmethyl)­amine, MeCN = acetonitrile, OTf^–^ = triflate, phen = 1,10′-phenanthroline) (Figure 1) for their closely related ligand environments, deliberately designed to differ in octahedral distortion and resulting small-to-medium ZFS parameters (*D* and *E*),^13,66^ to highlight the power of CTM.

**Figure 1:**
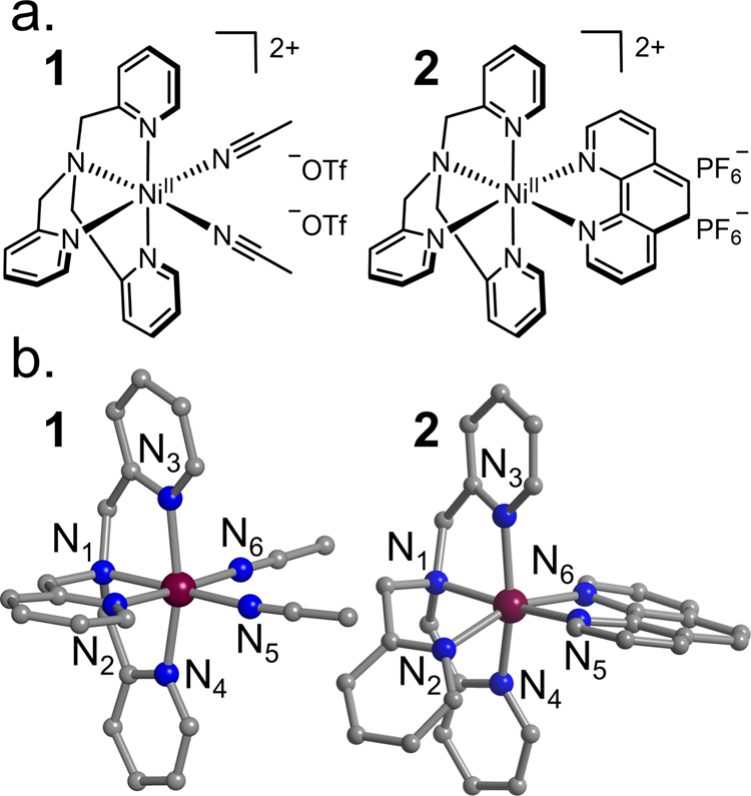
(a) Representation of [Ni­(tpa)­(MeCN)_2_]­(OTf)_2_ (**1**) and [Ni­(tpa)­(phen)]­(PF_6_)_2_ (**2**). (b) Cationic structures of **1** and **2** determined by X-ray diffraction. Counterions, lattice solvent, and hydrogen atoms are omitted. Color code: Ni (purple), C (gray), and N (blue).

Our study demonstrates that CTM enables high-precision determination of spin Hamiltonian parameters in molecular qubits using accessible laboratory instrumentation. This capability arises from CTM’s sensitivity to magnetic anisotropy, combined with a temperature-dependent protocol that experimentally decouples the effects of *g*-tensor anisotropy and ZFS. The resulting parameters are rigorously benchmarked against powder magnetometry, EPR spectroscopy, and *ab initio* CASSCF/NEVPT2 calculations, alongside a detailed analysis of experimental uncertainties and potential systematic errors establishing the reliability of the extracted values. Together, our results position CTM as a powerful and experimentally accessible method for the precise characterization of electronic structure parameters that govern the molecular qubit performance.

## Results and Discussion

Both **1** (monoclinic, *P*2_1_/*n*) and **2** (triclinic, *P*-1) exhibit [NiN_6_] distorted octahedral coordination (Figure 1, Table S5) (see Figures S1 and S2 for powder X-ray diffraction and infrared spectroscopy). Steric interactions between the phen and tpa ligands (N_2_, N_5_ rings, Figure 1) in **2** induce larger bond length asymmetry and octahedral angular deviation compared to **1** (Table S5, Figure S3).^67–69^ Both complexes feature an elongated axis along the N_pyridine_–N_MeCN_ (**1**) or N_pyridine_–N_phen_ (**2**) direction (N_2_–N_6_ bond axis, Figures 1, S4), with the elongation more pronounced in **2** (3.6%) compared to that in **1** (1.5%). Notably, several *cis* angles in **2** deviate more substantially from 90° (Figure 1, Table S5): N_1_–Ni–N_6_: **1** = 94°, **2** = 106°. N_2_–Ni–N_3_: **1** = 89°, **2** = 107°. N_5_–Ni–N_6_: **1 =** 88°, **2** = 106°. N_2_–Ni–N_4_: **1** = 92°, **2** = 80°. Global distortion metrics reinforce this observation, with the octahedral *SHAPE* index (0.62 vs 2.78), Σ (59° vs 117°), and Θ (165° vs 388°) all larger for **2**.^67–69^ To probe the effect of these distortions on the electronic structure of **1** and **2**, we analyzed the electronic absorption spectra within a classical ligand-field framework,^66,70–76^ which has long been applied to six-coordinate Ni­(II) complexes. From this analysis detailed in the Supporting Information and alongside solution- and single-crystal spectra of **1** and **2**, we extracted octahedral splitting, Δo (10Dq). The increased distortion in **2** reduces ligand field strength (Δo = 11780 cm^–1^) to a small degree relative to **1** (Δo = 11900 cm^–1^) (Figure S5). Complementary *ab initio* CASSCF/NEVPT2 ^77–79^ calculations provided the relative energy of the lowest triplet (^3^T_2_ and ^3^T_1_) and singlet (^1^E) excited states (Figure S6, Table S6, Supporting Information for more details), giving Δo of 12400 and 10630 cm^–1^ for **1** and **2**, respectively, reflecting the weaker ligand field in **2**.

Powder DC magnetometry shows that **1** and **2** have χ_M_ *T* values of 1.20 and 1.18 cm^3^ mol^–1^ K at 300 K, consistent with *S* = 1 and second-order orbital contributions (Figure 2).^80,81^ Both follow Curie–Weiss behavior between 10 and 300 K (Figure S7),^80^ below which χ_M_ *T* decreases to 0.96 and 0.80 cm^3^ mol^–1^ at 2 K for **1** and **2**, respectively, due to selective *m*_s_ depopulation from ZFS. The larger drop and lack of magnetization saturation in **2** (Figures 2, S8) indicate greater ZFS than in **1**. The DC and magnetization data are very well simulated (Figure 2) using the spin Hamiltonian $Ĥ=\mu_{B}B\cdot g\cdot Ŝ+D\left[{Ŝ}_{z}^{2}-\frac{1}{3}S\left(S+1\right)\right]+E\left({Ŝ}_{x}^{2}-{Ŝ}_{y}^{2}\right)$ and the best-fit parameters derived from CTM (*vide infra*). The difference in ZFS between **1** and **2** is supported by *ab initio* calculations (see Supporting Information for more details) using the complete active space self-consistent field (CASSCF) method, including corrections with the second-order *n*-electron valence state perturbation theory (NEVPT2) method^72–74^ (Figures S9, S10, Table S7).^77–79^ This method, which has been shown to be reliable in calculating the magnetic properties of Ni­(II) complexes,^21,31,82–84^ estimated *D* = 1.96 and −5.66 cm^–1^ for **1** and **2**, respectively, and *E*/*D* = 0.28 for both complexes. Simulation of the DC and magnetization data with the *g* values and ZFS determined using CASSCF/NEVPT2 generally captures the shape of the data, especially the larger drop in χ_M_ *T* at low temperature and poorer superimposition of the *M* vs *B*/*T* curves for **2** (Figure S8, S11) but clearly shows a difference in the calculated vs actual spin Hamiltonian parameters of both complexes. Given the small difference in Δo, the contrasting ZFS is more plausibly associated with differing elongation and angular distortion and distinct ligand π-bonding character: MeCN ligands are cylindrical π-interacting, whereas phen is not.^75^ Overall, the combination of structural similarity but differing ZFS makes **1** and **2** ideal to showcase CTM’s ability to extract *g*- and ZFS-tensor parameters with high precision using only microgram-scale crystals and a widely available magnetometry setup.

**Figure 2:**
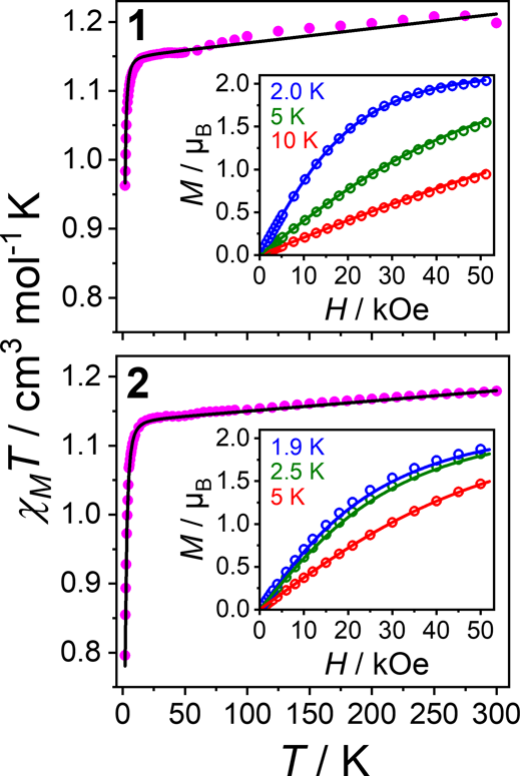
χ_M_ *T* vs *T* for **1** and **2**. Inset: magnetization versus field at three temperatures. Solid lines are simulations using CTM data best-fit parameters (Table 1) and inclusion of temperature-independent paramagnetism (TIP): **1**: *g_x_* = 2.1481, *g_y_* = 2.1628, *g_z_* = 2.1175, *D* = 1.80 cm^–1^, *E* = 0.152 cm^–1^, TIP = 2.1 × 10^–4^ cm^3^ mol^–1^. **2**: *g_x_* = 2.076, *g_y_* = 2.130, *g_z_* = 2.182, *D* = −3.895 cm^–1^, *E* = 1.264 cm^–1^, TIP = 1.5 × 10^–4^ cm^3^ mol^–1^. The kink at ∼50 K is due to the presence of a small amount of solid O_2_.

We conducted comprehensive magnetic anisotropy investigations of **1** and **2** using CTM (see Supporting Information for details).^50^ In the CTM, a single crystal is fixed to a miniature cantilever that acts as the movable piezoelectric plate. When placed in a homogeneous magnetic field, the sample experiences a magnetic torque (**τ** = **M** × **B**), which causes a measurable deflection of the cantilever.^50,85^ By rotating the crystal within the field, the angular dependence of this torque can be recorded, providing a direct probe of the magnetic anisotropy. At low field and high temperature, the torque varies linearly with the anisotropy of the magnetic susceptibility tensor, while at high field and low temperature, the angular dependence becomes more complex.^50^ In all cases, the torque signal vanishes every 90°, corresponding to field alignment parallel or perpendicular to the principal magnetic axis within the scanned crystallographic plane. Unlike powder magnetic susceptibility measurements, which involve a one-dimensional magnetometric effect (i.e., force along the field gradient axis), CTM measures a two-dimensional magnetometric effect (i.e., torque in the scanned *xy* plane).

Measurements are typically performed on indexed single crystals weighing tens of μg, though a few μg can be used,^86^ with linear dimensions comparable to those required for X-ray diffraction experiments (∼0.1–0.3 mm). Air-sensitive samples can also be measured,^86,87^ as demonstrated for **1**, by glovebox manipulation and indexing crystals on an X-ray diffractometer under an N_2_ gas flow. Mapping the full magnetic anisotropy generally requires rotation about three orthogonal axes; however, in cases of higher molecular symmetry, such as compounds with a single magnetically equivalent molecule possessing a *C*_3_ ^88^ or *C*_4_ ^89^ symmetry axis per unit cell, only two orthogonal rotations are needed (one along and one perpendicular to the symmetry axis). For **1** and **2**, CTM data were collected over three orthogonal rotation planes across 2–200 K and in magnetic fields between 1 and 9 T (Figure 3b for representative data sets; Figures S13–S18 for full data sets).

**Figure 3:**
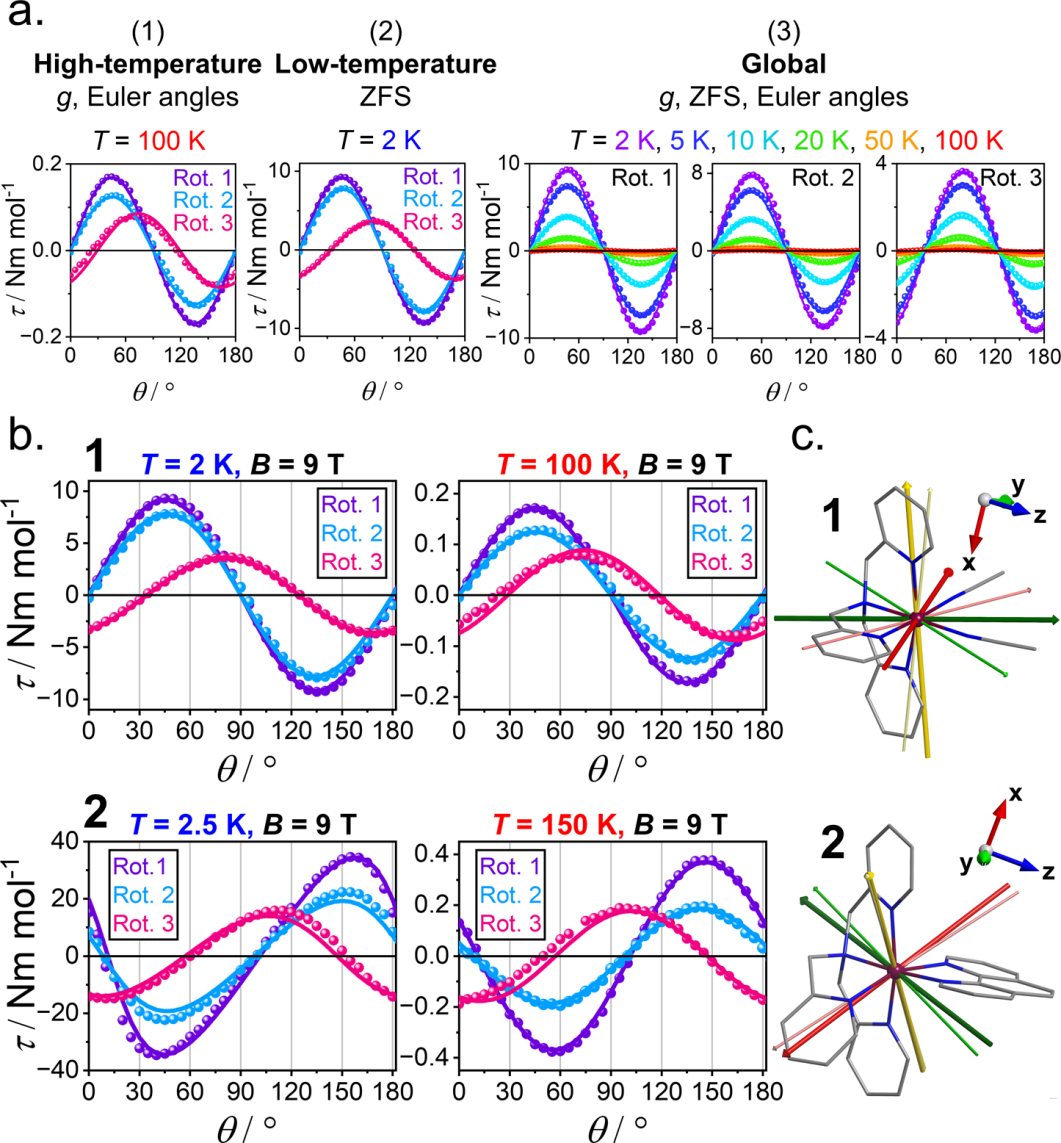
(a) CTM fitting protocol, represented for **1** at 9 T. (b) Torque signals for **1** and **2** at low and high temperatures for the three rotations (rot.). Solid lines are the final best fit. (c) Structures of **1** and **2** overlaid with experimental (thick; easy = dark green, intermediate = dark yellow, hard = dark red) and *ab initio* CASSCF/NEVPT2 (thin; easy = light green, intermediate = light yellow, dark = light red) magnetic reference frames. Easy axis (largest *g*) = *g_y_* for **1**, and *g_z_* for **2**. Intermediate axis (middle *g*) = *g_x_* for **1**, *g_y_* for **2** . Hard axis (smallest *g*) = *g_z_* for **1**, *g_x_* for **2**. Color code: Ni (purple), C (gray), N (blue). Hydrogen atoms were omitted.

Conventional CTM analyses typically perform a single global fit of the torque data across all measured temperatures to extract spin Hamiltonian parameters. However, this approach fits *g* and ZFS principal values and orientations simultaneously, reducing the ability to determine them with enhanced precision. Instead, we adopted a temperature-dependent protocol that exploits the distinct thermal behaviors of the magnetic anisotropy described by the *g* and *D* tensors to decouple and refine them independently. When ZFS is small (as for **1** and **2**), thermal energy at high temperatures populates all three *m*_s_ states essentially equally (ZFS ≪ *k*_B_ *T*), causing torque signals to be dominated by *g* anisotropy. As the temperature decreases and ZFS becomes comparable to *k*_B_ *T*, the Boltzmann distribution becomes increasingly weighted toward the lowest *m*_s_ level, while the higher levels remain partially populated. Changes in temperature alter this distribution, making the low-temperature torque primarily sensitive to ZFS. In contrast, for complexes with large ZFS, the higher energy states remain partially unpopulated even at elevated temperatures, with the torque signals significantly influenced by ZFS across all temperatures, preventing efficient *g* and ZFS decoupling. The small ZFS regime in our complexes enables a multistep strategy that isolates and then globally refines both contributions, enhancing the precision in the determination of the relevant parameters (***g***, *D*, *E*) in the spin Hamiltonian (same as used to simulate the powder magnetometry). Our fitting procedure follows three simple steps (Figure 3a): (1) fit high-temperature torque for principal *g* values and Euler angles assuming negligible ZFS. The position of zero-torque points shows little temperature variation indicating ***g*** and ***D*** tensors collinearity, allowing a fixed frame from the high-temperature fit; (2) fit low-temperature torque for ZFS (*D* and *E*), with fixed *g* values and Euler angles from the high-temperature fit; (3) global fit to refine all parameters simultaneously. *Ab initio* data can serve as initial guess parameters.

High-temperature fits (100 K for **1**, 150 K for **2**) are in very good agreement with experimental data (Figures 3, S19), yielding precise *g* values (Table 1). This enabled confident low-temperature fits (Figure 3, S19, 2 K for **1**, 2.5 K for **2**) to produce *D* and *E* values close to those obtained from the final global fit (Table 1). For **2**, the low-temperature fit yields *E*/*D* > 1/3, which compensates for inaccuracies in the *g* values derived from the high-temperature fit. These inaccuracies arise from residual ZFS effects at high temperature that are more pronounced in **2** than in **1**. Although *E*/*D* > 1/3 typically indicates the need to reorient the coordinate system, we intentionally maintained a fixed reference frame across all fits to ensure consistent and comparable analysis across temperature regimes. The final global fit refined all parameters (Figures 3, S13–S18), achieving principal *g* values precise to an impressive four decimal places for **1** and three for **2** (Table 1), as confirmed by sensitivity analysis evaluating the effect of parameter variations on fit residuals. Simulated torque data isolating one of the two spin Hamiltonian contributions confirm the dominance of ***g*** anisotropy at high temperatures and ZFS at low temperatures (Figure S20–S22) and restores the *E*/*D* < 1/3 condition. The precision with which our CTM protocol determines the *g* values appears to match or surpass the precision from previous HF-EPR studies on Ni­(II) complexes.^13,16,21,27,31,90–92^ We found that the precision of *g* values determined from CTM for **1** and **2** is higher than the values extracted from HF-EPR (*vide infra*).

**Table 1:** Spin Hamiltonian Parameters for **1** and **2** Derived from CTM, HF-EPR, and *Ab Initio*

	*g*_*x*_	*g*_*y*_	*g*_*z*_	*D*/cm^–1^	*E* /cm^–1^	*E*/*D*
CTM High-Temperature						
**1**	2.1505	2.1668	2.1074	-	-	-
**2**	2.0611	2.1298	2.1953	-	-	-
CTM Low-Temperature						
**1**	2.1505^a^	2.1668^a^	2.1074^a^	1.703	0.148	0.087
**2**	2.0611^a^	2.1298^a^	2.1953^a^	–3.847	1.358^b^	0.353^b^
CTM Global						
**1**	2.1481(1)	2.1628(1)	2.1175(1)	1.80(1)	0.152(1)	0.083
**2**	2.076(1)	2.130(1)	2.182(1)	–3.895(1)	1.264(1)	0.325
HF-EPR						
**1**	2.155(5)	2.155(5)	2.14(1)	1.55(5)	0.16(1)	0.103
**2**	2.14(1)	2.16(1)	2.21(1)	–4.25(5)	1.416(16)	0.333
*Ab initio* CASSCF/NEVPT2						
**1**	2.1836	2.1897	2.1712	1.961	0.549	0.280
**2**	2.1727	2.1957	2.2245	–5.660	1.585	0.280

a The values of *g* in the low-temperature fit are fixed from the high-temperature fit and are not derived from a fit of the low-temperature data.

b *E*/*D* > 1/3 reflects compensation for residual ZFS at high-temperture for **2**.

The larger magnitude of both *D* and *E* for **2** reflects its increased bond elongation and angular distortion of the octahedron compared to **1** (Table S5). The average value of *g* of ∼2.14 (**1**) and ∼2.13 (**2**) is consistent with reported octahedral Ni­(II) complexes, which typically span 2.05–2.3.^13,16,21,26,27,29–31,57,62,76,81,93–95^ The extracted ZFS values also fall within the reported range of NiN_6_ complexes (|*D*| ≈ 0.5–5 cm^–1^),^13,26,27,29,57,96^ placing **1** near the middle and **2** toward the upper end. By comparison, larger |*D*| values are common for mixed donor sets, with ∼3–12 cm^–1^ for N_4_O_2_ spheres,^27,28,30,56,61,81,91,96^ 4.4 cm^–1^ for N_3_O_3_,^92^ and ∼3–10 cm^–1^ for N_2_O_4_ spheres.^57,58,81,96^

The very high precision of our CTM-derived parameters arises from the deliberate decoupling of *g* and ZFS enabled by the temperature-dependent protocol combined with the high signal-to-noise ratio, mechanical stability, and thermal stability of the instrument. Indeed, the temperature-decoupling protocol used here substantially improves *g* precision compared to previous applications of CTM on Ni­(II) complexes; these studies were unable to decouple ***g*** and ZFS either because only low-temperature data were collected (<4 K)^63^ or the large *D* (∼22 cm^–1^) would have required temperatures far above the maximum of 30 K used to observe an effect.^64^ While ZFS governs qubit energy splitting and may critically affect coherence, the ***g*** tensor influences qubit control and magnetic response. Reducing the uncertainty in *g* values narrows the allowed ZFS range, improving overall accuracy. All parameters are well determined for **1** and **2** disregarding their crystal symmetries, highlighting CTM’s ability to disentangle noncollinear magnetic contributions.^50,86^

It is important to distinguish that precision reflects how finely the parameters can be extracted from the CTM data, whereas accuracy reflects their closeness to the true physical values. The latter is limited by systematic factors such as cantilever calibration, field homogeneity, temperature, field stability, crystal orientation, and crystal mass and molar mass determination. Many such contributions are inherent to single-crystal magnetic measurements and are rarely analyzed in detail because they are difficult to quantify. These effects were minimized through strict control of measurement conditions (see Supporting Information for technical details). Because the crystal and instrument remain fixed across all temperature and field sweeps, these systematic factors act as constant scale offsets that influence the absolute accuracy but not the relative precision. To confirm that the small temperature and field uncertainties do not influence the extracted parameters, we regenerated the CTM data for **1** after artificially shifting the temperature by ± 0.01 K (Figure S23, Table S8 for measured stability and set-point deviations) and the magnetic field by ±1 mT (Figures S24, S25). Refitting these modified data sets produced no changes in *D* or *E* and variations in *g* within uncertainty. Crystals were weighed on a microbalance with microgram (±1 μg) precision. Like the temperature and field, we regenerated the CTM data for **1** by changing the mass by ±1 μg (Figure S26), with fitting the modified data sets also producing no change in *D* or *g* values and variation in *E* within uncertainty. Crystal misalignment with respect to the expected orientation could be a major source of systematic error in ***g*** and ZFS determination using CTM, and the same can in principle happen in single-crystal HF-EPR.^24,35–37,97,98^ For HF-EPR, the principal values of the ZFS (and ***g***) tensor can be retrieved from powder spectra, for which the accuracy of the ZFS parameters is governed by the precision of the microwave frequency. Crystal orientations were adjusted optically, and the effect of mounting uncertainty was assessed by deliberately removing and remounting the crystal of **2** three times at a known arbitrary angle (Figure S27). The resulting 100 K torque curves were nearly identical, and each data set was reproduced using the same spin Hamiltonian parameters (Figure S27), further confirming the validity of the extracted parameters. The lattice-solvent content of **2** was verified on the exact crystal used for CTM via single-crystal X-ray diffraction, with precautions taken to prevent solvent loss (rapid transfer, grease coating, and low-temperature mounting). The minimal loss of lattice solvent was further confirmed using the above orientation reproducibility test: the same crystal of **2**, after six months of ambient storage, was subjected to three measurement cycles in which it was cooled to 100 K, measured, warmed to 300 K, removed, and remounted at the new arbitrary angle. All three 100 K torque curves were essentially identical and were reproduced with the same spin Hamiltonian parameters (Figure S27).

We acknowledge that beyond the sources of systematic error explicitly investigated here, additional effects could, in principle, influence the absolute accuracy of CTM-derived parameters. These include crystal shape anisotropy, sample dimensions relative to the region of magnetic field homogeneity, potential anisotropy in temperature-independent paramagnetism (TIP), and other instrumental factors affecting torque detection (see Supporting Information). While such effects could affect absolute accuracy, the CTM uncertainties quoted here represent the statistical precision of the global fit under stable, well-calibrated conditions. Our aim is not a full metrological deconvolution of every instrumental contribution but to demonstrate the analytical power and attainable precision of CTM when implemented under standard, reproducible laboratory conditions.

Euler angles extracted from CTM fitting for **1** (monoclinic) yield two possible magnetic reference frames, as expected for a system containing two magnetically inequivalent molecules (Figures 3, S28; Supporting Information for Euler angles). In contrast, **2** (triclinic) contains only one magnetically inequivalent molecule and therefore has a single CTM solution (Figure 3). To distinguish between the two possible orientations for **1**, we compared the CTM-derived axes with CASSCF/NEVPT2 calculations (Figures 3, S9). The angular deviations between the calculated and experimental axes for solution 1 (Figure 3) are 12.4°, 40.2°, and 38.4°, whereas for solution 2 (Figure S28), they are 44.0°, 24.4°, and 36.6°. Although neither solution yields perfect agreement, solution 1 exhibits a smaller overall deviation and is therefore assigned as the more reasonable orientation. Multiconfigurational *ab initio* approaches, including CASSCF/NEVPT2, reliably reproduce ZFS magnitudes and magnetic-axis orientations for both lanthanide^99–103^ and transition-metal complexes,^104–111^ including Ni­(II).^21,27,31,64,82–84^ Benchmarking calculated axes against CTM results has been reported for lanthanide complexes^87,99,100,112^ and highly anisotropic transition-metal systems.^64,111,113^ This work expands the number of systems for which *ab initio* predictions have been compared directly with CTM-derived axes. Agreement is very good for **2**, whereas for **1**, the correspondence is less satisfactory, reflecting the challenges associated with the magnetic-axis orientation prediction. For both complexes, the easy axis (largest *g*; *g*_*y*_ for **1**, *g*_*z*_ for **2**) aligns approximately along the tpa N_amine_ bond (N_1_–N_5_ axis), while the hard axis (smallest *g*; *g*_*z*_ for **1**, *g*_*x*_ for **2**) lies roughly along the elongated N_pyridine_ bond (N_2_–N_6_) (Figures 1, S4).

The experimental trends are overall supported by CASSCF/NEVPT2 calculations (Table 1), aligning with this method’s reliability in the determination of magnetic properties of Ni­(II).^21,31,82–84^ The larger and more rhombic *g*-anisotropy of **2** relative to **1** is reproduced: experimentally, |*g*_hard_ – *g*_intermediate_| and |*g*_easy_ – *g*_intermediate_| are 0.031 and 0.016 for **1** and 0.054 and 0.052 for **2**; calculations give 0.012 and 0.006 for **1** and 0.023 and 0.029 for **2**. The calculated *D* values of 1.96 cm^–1^ (**1**) and −5.66 cm^–1^ (**2**) agree qualitatively with CTM-derived values of 1.80 and −3.90 cm^–1^, with agreement improved when excited singlet states are included (Table S7). Crucially, calculations capture the change in the sign of *D* between the two compounds. While *D* > 0 for **1** is consistent with axial elongation along the N_2_–N_6_ (Figures 1, S4, N_py_–N_MeCN_),^96^ the large rhombicity and *D* < 0 for **2** highlight how angular distortion and subtle π-donor/acceptor and σ-donor effects complicate the prediction of the sign of *D* from structure alone.^83^ While the CASSCF/NEVPT2 calculations successfully replicate the experimental sign of the axial ZFS parameter for both complexes, some quantitative discrepancies are noted for compound **1**. Specifically, the calculated rhombicity (*E*/*D* = 0.27) and the near-isotropic ***g*** tensor deviate from the CTM-derived values (Table 1). These deviations are common in multiconfigurational treatments including only the 3d orbitals inside the active space,^114,115^ as they can struggle to capture the full extent of dynamic correlation and metal–ligand covalency, factors that significantly influence the magnetic anisotropy and electronic relaxation in compound **1**. The CTM-derived *E*/*D* of **2** (0.32) lies very close to the fully rhombic limit. In this regime, small parameter variations can lead to ambiguity in principal-axis assignment and complicate determination of the sign of *D*. Nonetheless, the global CTM fit, supported by multiconfigurational *ab initio* calculations, supports the assigned sign of *D* for **2**. Even when *D* is minimized (−3.894 cm^–1^) and *E* maximized (1.265 cm^–1^) within their CTM uncertainties, *E*/*D* remains below 1/3 in the same reference frame.

Having compared CTM and *ab initio*, we next turned to independent experimental benchmarks. The CTM spin Hamiltonian parameters reproduce powder DC susceptibility and variable-field magnetization data extremely well (Figure 2). The simulations required inclusion of TIP of 2.1 × 10^–4^ and 1.5 × 10^–4^ cm^3^ mol^–1^ for **1** and **2**, respectively, which are within the range reported for pseudo-octahedral Ni­(II) complexes^30,84,93,95,116,117^ and do not affect the CTM curves, assuming that TIP is an isotropic contribution to the susceptibility. The high-temperature χ_M_ *T* values support that the average *g* values derived from CTM are correct (Figure 2); we note, however, that even in the case in which powder magnetization was not reproduced adequately, an average *g* from χ_M_ *T* can be used to fit the CTM data without affecting the well-determined Δ*g* feature.

Following powder magnetometry, we turned to a comparison with spectroscopy. It is important to emphasize the distinct and complementary information provided by EPR and CTM. EPR is a spectroscopic technique and, particularly in single-crystal and multifrequency implementations, provides microscopic insight into magnetic parameter distributions, line widths, and strain effects reflecting local environments.^16,118,119^ In contrast, CTM is a thermodynamic measurement that probes the ensemble-averaged magnetic anisotropy and yields the central, bulk-effective spin Hamiltonian parameters that govern the macroscopic magnetic response. For **1**, CTM indicates an *E* value (0.152 cm^–1^) close to half the X-band microwave frequency (*hν* ≈ 0.3 cm^–1^). Consistent with this, continuous-wave (cw) X-band EPR reveals a peak near ∼70 mT in the perpendicular mode (selection rule Δ*m*_s_ = ±1) and ∼40 mT in the parallel mode (selection rule Δ*m*_s_ = 0) (Figure 4). Simulating both spectra using EasySpin,^120^ fixing *g* and *D* from CTM, yields an exceptionally precise value of *E* = 0.15615(5) cm^–1^. Simulations using *E* = 0.156, 0.1561, and 0.1562 cm^–1^ (Figure S29) did not reproduce the spectra as well. This level of precision of *E* surpasses typical EPR-only analyses and illustrates how CTM provides a way to refine otherwise challenging parameters. Zeeman plots (Figure S30) confirm that the observed peak corresponds to a near-zero-field *m*_s_ ≈ |+1⟩ and *m*_s_ ≈ |−1⟩ transition with energy gap 2*E* (Figure 4). Compound **1** could display decoherence-protected clock transitions, enhancing coherence times by reducing sensitivity to magnetic field fluctuations.^16,84^ As expected, due to its large *D* and *E* values, **2** is EPR silent at X-band.

**Figure 4:**
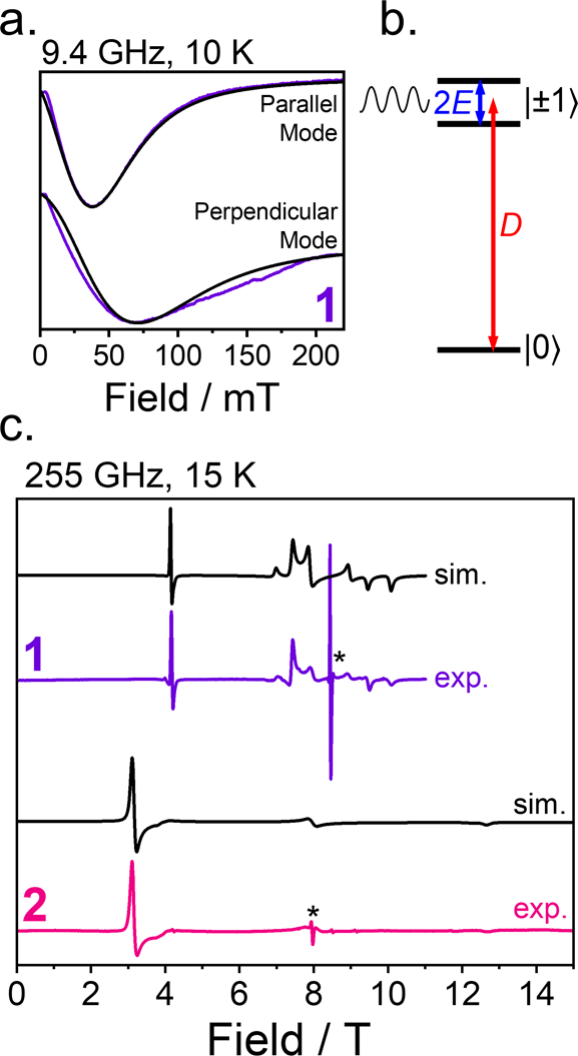
(a) Continuous-wave X-band EPR spectra of **1** (blue line) in perpendicular (microwave frequency = 9.387403 GHz) and parallel (microwave frequency = 9.382712 GHz) modes at 10 K. Black lines are simulations using parameters *g_x_* = 2.1481, *g_y_* = 2.1628, *g_z_* = 2.1175, *D* = 1.80 cm^–1^, and *E* = 0.15615 cm^–1^. Line broadening of [3 60] and [20 39] were used for perpendicular and parallel modes, respectively. Line broadening parameters are given in the EasySpin^120^ notation [Gaussian, Lorentzian], expressed as full widths at half-maximum in MHz. (b) Energy ordering of the ground *S* = 1 state of **1,** resulting from *D* and *E*, showing EPR transitions between *m*_s_ ≈ |+1⟩ and *m*_s_ ≈ |−1⟩. (c) Continuous-wave high-frequency experimental (exp.) EPR spectra of **1** and **2** at 255.36 GHz at 15 K (* denotes double quantum transition). Best simulation (sim.) in black: **1**; *g_x_* = 2.155(5), *g_y_* = 2.155(5), *g_z_* = 2.14(1), *D* = 1.55(5) cm^–1^, *E* = 0.16(1) cm^–1^, **2**; *g_x_* = 2.14(1), *g_y_* = 2.16(1), *g_z_* = 2.21(1), *D* = −4.25(5) cm^–1^, and *E* = 1.416(16) cm^–1^.

Due to the energy of its microwave frequency, X-band EPR provided information only on *E* for complex **1**. To establish an experimental benchmark for comparison with CTM and to assess the precision of the principal *g* values obtained from our protocol, we measured multifrequency, variable-temperature HF-EPR on **1** (128 GHz at 5–125 K and 255 GHz at 5–15 K) and **2** (221, 255, and 331 GHz at 5–15 K) (Figures 4, S31–S35). Magnetic resonances were observed across all frequencies and temperatures. Simulations of these spectra using the CTM-derived parameters did not reproduce the resonance positions (Figures S31, S32). Instead, satisfactory simulations were obtained using a single set of HF-EPR parameters for each compound: for **1**, *g*_*x*_ = 2.155(5), *g*_*y*_ = 2.155(5), *g*_*z*_ = 2.14(1), *D* = 1.55(5) cm^–1^, and *E* = 0.16(1) cm^–1^; and for **2**, *g*_*x*_ = 2.14(1), *g*_*y*_ = 2.16(1), *g*_*z*_ = 2.21(1), *D* = −4.25(5) cm^–1^, and *E* = 1.416(16) cm^–1^ (Table 1, Figures 4, S33–S35). These parameters are in line with expectations for pseudo-octahedral Ni­(II) systems.^13,16,21,26,27,29–31,57,62,76,81,93–95^ The CTM and HF-EPR values show moderate differences; *g* values deviate by 0.6% (**1**) and 1.3% (**2**); *D* differs by 14% (**1**) and 8% (**2**); and *E*/*D* remains similar. These differences prompted further investigation.

For both complexes, the HF-EPR parameters did not simulate the CTM torque curves (Figures S36–S43), nor did they reproduce the powder magnetic data (Figure S44), whereas the CTM-derived parameters accurately reproduced both CTM and powder measurements. Several possible sources of error in the CTM measurements were assessed. Mass errors were excluded because they would lead to a simple rescaling without correcting for the temperature dependence (e.g., mass-error compensation for powder magnetometry of **2**, Figure S45). Attempts to refit the CTM data for **1** and **2** using the HF-EPR parameters while allowing the mass to vary converged to an unphysical value (∼75% of the true mass) for **1** with a poor fit, and +1 μg for **2** also with a poor fit (Figure S46). Likewise, fits in which the Euler angles were varied while fixing the HF-EPR parameters for **1** did not converge, and for **2** yielded a poor fit (Figure S47), indicating that orientation uncertainty cannot account for the differences. This aligns with earlier discussion that crystal alignment (Figure S27), as well as temperature and field fluctuations (Figures S25 and S26), do not produce significant errors. Lastly, we attempted to fit the CTM for **1** and **2** using starting values as derived from HF-EPR and freely fitting for *g*, *D*, *E*, and Euler angles; no meaningful fit was achieved. We note that the discrepancy is also unlikely to arise from a powder versus single-crystal effect, as the (single-crystal) CTM parameters quantitatively reproduce the powder magnetometry data. Furthermore, the field positions of the principal transitions (especially the half-field transition) in the HF-EPR spectra of **1** collected on unground crystals are unchanged relative to the ground (powdered) sample (Figure S48), and a preliminary single-crystal W-band (94 GHz) spectrum of **1** (field parallel to −c*) is better simulated with the HF-EPR parameters (Figure S49).

Taken together, these observations indicate that the discrepancy between CTM- and HF-EPR-derived parameters does not originate from experimental artifacts, sample handling, mass determination, orientation uncertainty, grinding, or solvent retention. As discussed earlier, additional systematic contributions not explicitly quantified here could, in principle, affect the absolute accuracy of the CTM-derived parameters. Although we cannot assign a single definitive origin to the observed differences, the consistency between thermodynamic techniques (CTM and DC magnetometry) hints toward a difference based on the type of detection (i.e., magnetometric vs spectroscopic).

The present study constitutes a test case for cross-technique comparison: the systems exhibit small magnetic anisotropy, minimal exchange interactions, high sample quality, and very high-quality CTM and HF-EPR data sets. Even under these favorable conditions, systematic differences in the spin Hamiltonian parameters are observed between the two approaches. These results demonstrate that perfect quantitative agreement between high-precision magnetometry (here, CTM) and magnetic resonance (here, HF-EPR) is not guaranteed, even in a best-case scenario. We propose that the level of agreement achieved here, approximately ∼1% for *g* and ∼5–15% for ZFS, provides a realistic benchmark for quantitative comparisons between CTM and EPR in molecular spin systems.

The HF-EPR measurements allow a comparison of practical precision with CTM. In this data set, the CTM-derived *g*-values exhibit higher numerical precision than those obtained from HF-EPR. In addition, CTM yields formal numerical uncertainties via global data fitting, whereas the HF-EPR spectra collected in this study are simulated rather than fitted and therefore do not provide comparable statistical errors. The precision of the spin Hamiltonian parameters derived from HF-EPR could be improved using very high magnetic fields, where the Zeeman interaction dominates and separates *g*-anisotropy from ZFS, or through the construction of frequency-versus-field plots, whose slopes yield *g* and intercepts/curvature reflect ZFS, allowing for a fit and therefore deduction of numerical uncertainties.^24,52,121–123^ However, again, these measurements require specialized facilities and resources. HF-EPR can also achieve very high precision in ideal cases with intrinsically narrow line widths. In many molecular systems, including qubit candidates, line widths are limited by inhomogeneous broadening rather than dipolar interactions, such that magnetic dilution does not significantly sharpen cw spectra. In cases where line widths are dipolar-broadened, dilution into an isostructural diamagnetic host can be effective, enabled by EPR’s exceptional spin sensitivity. It is possible that dilution could improve the line width of the signal for **1** and **2** and therefore the precision of the extracted parameters. However, no suitable host exists for **1**: the Zn­(II) analogue is not isostructural ([Zn­(tpa)­(MeCN)]^2+^),^124^ and the low-spin Fe­(II) analogue undergoes spin crossover in solution^125^ and remains partially high-spin in the solid state (Figure S50).

On the other hand, HF-EPR provides complementary advantages associated with its spectroscopic nature; it has allowed for direct assessment of possible temperature-dependent changes in the spin Hamiltonian. For **1**, resonances remain observable up to 125 K without measurable shifts (Figure S34), indicating a temperature-invariant spin Hamiltonian across 5–125 K. For **2**, CASSCF/NEVPT2 calculations on structures obtained at 100 K and 293 K and on an optimized 0 K geometry likewise predict minimal variation in *g*, *D*, and *E*/*D* values (Figure S51, Table S9). These findings mirror those reported for a Dy­(III) system.^126^ The ability to simulate the powder magnetic susceptibility with one set of parameters (Figure 2) supports the idea that the spin Hamiltonian does not change with temperature. When available, HF-EPR therefore remains the benchmark experimental technique for the direct spectroscopic determination of *g* and ZFS parameters. However, the results presented here demonstrate that CTM can achieve quantitatively comparable spin Hamiltonian information using only standard laboratory equipment, supporting its broader adoption as a precise and accessible tool for characterizing the electronic structure of molecular qubit candidates.

To guide future CTM studies and better frame the potential of the method, we assessed how ZFS magnitude affects *g*-value precision and overall spin Hamiltonian accuracy. In our Ni­(II) systems, the difference (mean absolute error, MAE, see Cantilever Torque Magnetometry in the Supporting Information section, eq S1) between *g* values obtained from high-temperature and global fits is small: 0.0053 (0.25%) for **1** and 0.0095 (0.45%) for **2** (Table 1). However, comparison of **1** and **2** shows that larger |*D*| values increasingly influence high-temperature torque data. For **2**, the residual ZFS contribution at high-temperature was sufficient to drive the low-temperature fit to *E*/*D* > 1/3. This highlights a broader principle: as ZFS increases, the ability to decouple *g* and ZFS, and thus extract precise spin Hamiltonian parameters with CTM, diminishes.

To generalize this effect and investigate the range of ZFS magnitudes that allow for high precision in *g*-value determination, we generated simulated “experimental” torque curves at 100 K and 9 T using the experimental parameters of **2**. We imposed *g* values of 2.00, 2.02, and 2.04, which we define as *g*_set_ (assigned to *g*_*x*_, *g*_*y*_, *g*_*z*_ depending on the sign of *D*), across a range of |*D*| from 0.05 to 5 cm^–1^ assuming *E*/*D* = 1/3 (Figure S52). The values of Δ*g* are comparable to those observed for molecular qubits.^13,14,16,21^ These simulated curves were then fit for just *g*, denoted *g*_fit_ (Table S10). The difference between *g*_set_ and *g*_fit_ provides a measure of the CTM precision as a function of |*D*|. We observed a linear increase in MAE between *g*_set_ (2.00, 2.02, 2.04) and *g*_fit_ (Table S10), described by MAE = 0.0034 × |*D*| (Figures 5, S53). This predicts MAE for **1** and **2** of 0.006 and 0.013, respectively, close to the experimentally observed difference between the high-temperature and global fit of *g*. Based on these findings, we identify three regions that define plausible *g*-value precision when determined from our CTM protocol (maximized signal-to-noise) (Figure 5): three decimal places (red, |*D*| > 3 cm^–1^), four decimal places (blue, 0.3 < |*D*| < 3 cm^–1^), and five decimal places (green, |*D*| < 0.3 cm^–1^). For **1** and **2**, where ZFS is modest, the *g* values from the high-temperature fit already agree well with the global fit (Table 1); Figure 5 makes clear that such agreement would not hold for systems with larger ZFS.

**Figure 5:**
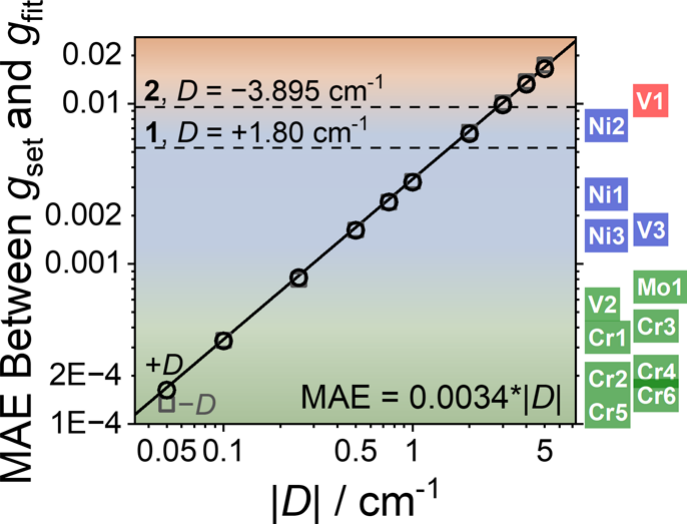
Correlation of |*D*| with mean absolute error (MAE) between *g*_set_ and *g*_fit_ (as defined in the text). Solid line is a linear regression fit (*R* ^2^ = 0.999), with the inset equation. Black circles represent +*D* values, and grey squares represent −*D* values. Color regions represent plausible precision of *g* values determined using CTM. Reported *S* = 1 molecular qubits on the right (Figure S54) are ordered by predicted MAE based on experimental |*D*| values.^12–14,21–23^

By projecting *D* values (0.04–3.6 cm^–1^) for *S* = 1 optically addressable molecular qubits (Figure S54) onto our analysis (Figure 5), we predict that CTM can achieve *g*-factor precision to five decimals for Cr­(IV) and four decimals for V­(III) and Ni­(II) complexes, achieving comparable precision to existing powder EPR results on the same compounds.^12–14,21–23^ This performance arises from CTM’s ability to disentangle *g*-anisotropy from ZFS within a single experiment. Beyond precision, CTM offers several other practical advantages: it routinely operates on microgram-scale single crystals, does not rely on line width narrowing or magnetic dilution, and is equally applicable to integer- and half-integer spin systems. We emphasize that these predictions represent a working hypothesis based on two experimentally validated benchmark systems and that their broader generality needs to be confirmed through future studies. This would include extending our CTM protocol to other spin multiplicities, such as *S* = 3/2 and 2, to see if the same principles explored here hold. Such studies are underway and will allow for a continued assessment of the regimes in which the CTM delivers the highest achievable precision.

## Conclusion

We demonstrate here that cantilever torque magnetometry enables high-precision bulk-mean value determination of spin Hamiltonian parameters in molecular qubits using widely available laboratory instrumentation. Using two closely related octahedral Ni­(II) complexes with small-to-moderate ZFS as benchmark systems, we establish a temperature-based CTM protocol that experimentally separates ***g***-tensor anisotropy and ZFS. This capability positions CTM as a powerful and experimentally accessible method for characterizing low-anisotropy integer-spin systems relevant to molecular quantum information science.

While HF-EPR remains the gold-standard technique for qubit analysis, providing exceptional precision, sensitivity to low spin concentrations, and detailed information about relaxation and coherence, CTM offers a complementary route to quantitative magnetic anisotropy determination. The two techniques probe distinct but mutually informative aspects of a spin system: CTM measures the bulk-averaged thermodynamic response, whereas EPR provides a spectroscopic view of microscopic distributions. Although still underused in molecular qubit research, CTM can be implemented on standard commercial magnetometry platforms (e.g., PPMS) equipped with a relatively low-cost cantilever insert, enabling true laboratory-scale, accessible high-precision anisotropy mapping without the need for dedicated HF-EPR infrastructure. Like single-crystal EPR, CTM requires well-oriented single crystals, which can pose experimental challenges for systems that do not crystallize readily; however, this constraint is offset by its minimal sample requirements, demonstrated precision, and broad accessibility. We emphasize that while the precision reported here reflects the CTM protocol of decoupling *g* and ZFS, the absolute accuracy of the extracted parameters is bounded by systematic factors inherent to all single-crystal techniques.

This work shows that even under favorable conditions, perfect quantitative agreement between magnetometric and spectroscopic approaches is not necessarily expected. The level of agreement observed here therefore represents a realistic expectation for comparisons between high-precision magnetic resonance and magnetometry in molecular spin systems.

By showcasing CTM’s ability to deliver precise spin-Hamiltonian parameters from minimal single-crystal sample quantities using standard instrumentation, we anticipate that our study will encourage broader adoption of CTM. Together, these features position CTM as a powerful technique for accurate characterization of low-anisotropy spin systems, including optically addressable qubits,^12–14^ magnetic refrigerants,^127–129^ and subtle *g*-shifts under electric field modulation,^130^ improving atomistic tuning and the design of next-generation quantum materials.

## Supplementary Material



## Notes

Experimental procedures, synthesis, crystallography, powder X-ray diffraction, IR, magnetic measurements, UV–vis spectroscopy, *ab initio* calculations, EPR, and cantilever torque magnetometry (PDF)
