# Supplementary material for: Benchmarking Cantilever Torque Magnetometry as a Platform for Characterizing Molecular Qubits: A Case Study on Ni(II) Complexes
Source: J Am Chem Soc. 2026 Mar 5;148(10):11260–73. doi: 10.1021/jacs.6c00500 (PMC13003497; doi:10.1021/jacs.6c00500)
Supplement: Supplementary file 1 [file ja6c00500_si_001.pdf]

Supporting Information to accompany

Benchmarking Cantilever Torque Magnetometry as a Platform for Characterizing Molecular Qubits: a Case Study on Ni(II) Complexes

Jett T. Janetzki,[1] Arsen Raza,[1] Matteo Briganti,[1] Rocco Duquennoy,[2,3] Anne-Laure Barra,[4] Costanza Toninelli,[2,3] Mauro Perfetti,*[1] and Lorenzo Sorace*[1]*

[1] Department of Chemistry "Ugo Schiff" and INSTM Research Unit, University of Florence, Via della Lastruccia, 3, 50019, Sesto Fiorentino, Italy

[2] European Laboratory for Non-Linear Spectroscopy (LENS), Via Nello Carrara 1, 50019, Sesto Fiorentino, Italy

[3] National Institute of Optics (CNR-INO), Via Nello Carrara 1, 50019, Sesto Fiorentino, Italy

[4] Laboratoire National des Champs Magnétiques Intenses, CNRS, Université Grenoble Alpes, 25 Avenue des Martyrs, 38042 Grenoble, France

EXPERIMENTAL

Synthesis

All manipulations, unless otherwise indicated, were performed under anaerobic conditions in an M-Braun N_2-atmosphere glove box or on a Schlenk line with N_2 gas using standard Schlenk techniques. All chemicals purchased were of reagent grade or higher and used as received. Acetonitrile (MeCN) was dried over molecular sieves (3 Å) for a minimum of 3 days and stored under N_2 on molecular sieves until used. Dry diethyl ether (Et_2O) was purchased from Sigma-Aldrich and stored under N_2 on molecular sieves until used. Toluene was distilled for K and stored under N_2 on molecular sieves. Solvents were degassed prior to use via three freeze-pump-thaw cycles. Molecular sieves were activated by heating at 300 °C for 48 hours and then cooled under vacuum. Complex $[Ni(tpa)(MeCN)_2](OTf)_2$ (**1**) was prepared by minor variation of a literature procedure (see below).[1] Ligand tpa[2] and compounds $[Ni^{II}(tpa)(H_2O)(OAc)]PF_6$ [3] and $[Fe^{II}(tpa)(MeCN)_2](OTf)_2$ [1] were prepared according to literature procedures.

$[Ni(tpa)(MeCN)_2](OTf)_2$ (1). In a glovebox, to a stirring solution of $Ni(OTf)_2$ (120 mg, 0.34 mmol) in 6 mL MeCN was added a solution of tpa (100 mg, 0.34 mmol) in 6 mL MeCN, resulting in a pale-yellow suspension. The reaction mixture was left stirring for 72 hours at room temperature, after which the reaction was filtered to yield a pink solution. The filtrate was concentrated under reduced pressure until viscous, before being separated into three 1.5 mL solutions which were each layered below 5 mL Et_2O. After 24 hours, pink-purple blocks formed (200 mg, 80%). Anal. Calcd for $C_{24}H_{24}N_6F_6O_6S_2Ni$: C, 39.53; H, 3.32; N, 11.52. Found C, 39.72; H, 3.52; N, 11.29. Selected FT-IR data (ATR, cm^{-1}): 1607 (m), 1443 (m), 1252 (s), 1141 (s), 1027 (s), 912 (w), 763 (m), 634 (s). UV-Vis (acetonitrile) λ_{max} (ε): 535 (20), 840 nm (24 L mol^{-1} cm^{-1}).

$[Ni(tpa)(phen)](PF_6)_2$ (2). In air, to a stirring solution of $[Ni(tpa)(H_2O)(OAc)]PF_6$ (140 mg, 0.25 mmol) in 3.5 mL MeOH was added solid KPF_6 (58 mg, 0.32 mmol). The solution

was cooled on ice, before adding 1,10'-phenanthroline (43 mg, 0.24 mmol) in 3.5 mL MeOH dropwise. A solid began to form, and the reaction was stirred on ice for 2 hours. The precipitate was vacuum collected in air and washed with MeOH and Et_2O to yield [Ni(tpa)(phen)]$(PF_6)_2$ as a purple precipitate (0.17 g, 85%). Layering a saturated MeCN solution with Et_2O yielded purple blocks of **2**·MeCN suitable for X-ray diffraction. The crude product was recrystallized by dissolving 150 mg of **2** in 4.5 mL MeCN, filtering, and adding 15 mL Et_2O dropwise to yield a microcrystalline solid, analyzing as **2**·0.5MeCN (130 mg). Anal. Calcd for $C_{30}H_{26}N_6P_2F_{12}Ni{\cdot}0.5CH_3CN$: C, 44.34; H, 3.30; N, 10.84. Found C, 44.64; H, 2.94; N, 10.62. Selected FT-IR data (ATR, cm^{-1}): 1609 (m), 1519 (w), 1448 (m), 1427 (m), 827 (s), 761 (s), 727 (s), 555 (s). UV-Vis (MeCN) λ_{max} (ε): 890 nm (20 L mol^{-1} cm^{-1})

X-ray Data Collection and Structure Solution

Single X-ray diffraction data for **2**·MeCN was collected at 100 and 293 K using a Bruker D8 Venture diffractometer equipped with a PHOTON II detector and a microfocus source (Cu Kα radiation, λ = 1.54178 Å). Crystals were transferred directly from the mother liquor to crystallographic oil to prevent solvent loss and loss of crystallinity. Data were corrected for absorption effects using the multiscan method (SADABS). Structures were solved with SHELXT[4] and refined using a full matrix least squared procedure based on F^2 using SHELXL within Olex2.[5,6] All non-hydrogen atoms were refined using anisotropic displacement factors. Hydrogen atoms were placed at geometrical positions and refined using the riding model.

Powder X-ray diffraction (PXRD) data for **1** was measured on a Rigaku Synergy Dual Wavelength Rotating Anode X-ray Diffractometer System using Cu-Kα (λ = 1.54 Å) at 100 K. Powder samples were prepared by crushing the sample gently and creating a slurry with crystallographic oil. Data were collected at 2θ = 5 – 50° with an exposure time of 60 seconds per frame and processed using CrysAlisPro. PXRD data for **2** was measured on an Anton Paar

XRDynamic 500 X-ray Diffractometer system using Co- Kα (λ = 1.79 Å) at room temperature. Data were collected at 2θ = 3 – 70° with an exposure time of 120 seconds per frame, and then converted to Cu-Kα (λ = 1.54 Å). Crystalline powder was mounted on a zero-background diffraction plate. Powder X-ray diffraction verified bulk samples of **1** and **2** have the same phase as the single crystals (Figure S1).

Infrared and UV-Vis Spectroscopy

Attenuated total reflectance infrared spectra were measured on a Bruker Alpha spectrometer and normalized as absorbance spectra. Solution state UV-Vis spectra were measured on a Jasco V-670 double-beam spectrophotometer at room temperature in MeCN. Solution UV-Vis measurements samples for **1** were prepared in an M-Braun N_2-atmosphere glove box and placed in a gastight cuvette.

Single Crystal Absorption Measurements

Low-temperature single crystal absorption measurements were performed in a Montana Cryostation cryostat at 3.5 K. Crystals of **1** and **2** were placed in sealed glass cells to avoid crystal sublimation during the vacuum pumping cycle of the cryostat. In the case of **1**, the sealed cell is closed in an M-Braun N_2-atmosphere glove box. The crystals are illuminated with a Thorlabs OSL1 tungsten 3200 K lamp. The transmitted light is analyzed on an Andor Shamrock 303i spectrometer equipped with an Andor iXon3 EMCCD camera. The absorption spectrum S_abs is obtained as

$$S_{||} = 1 - \frac{S_t - S_{bg}}{S_{wl} - S_{bg}}$$

where S_t and S_{wl} are the spectra of the light transmitted by the crystal and by the glass of the cell alone, respectively. S_{bg} is the spectrum measured in the absence of light, that needs to be

subtracted from S_t and S_{wl} to correct for the dark counts of the spectrometer's camera. To obtain the right panel of Figure S5, the raw data S_t, S_{wl} and S_{bg} have been smoothed with a gaussian filter to lower the effect of the read-out noise of the spectrometer's camera.

Magnetic Measurements

The DC and AC susceptibility and magnetization measurements for **1** and **2**·0.5MeCN were performed on a Quantum Design Magnetic Properties Measurement magnetometer equipped with a Superconducting Quantum Interference Device Direct. The samples were prepared by wrapping the powder of **1** or **2**·0.5MeCN in Teflon and pressing into a pellet. The temperature-dependent DC measurements for **1** were performed between 2.0–300 K with an applied field of 0.1 T. The temperature-dependent DC measurements for **2**·0.5MeCN were performed at 0.1 T between 2.0–28 K and 1 T between 30–300 K. The field-dependent magnetization measurements were performed at 2, 5 and 10 K for **1** and 1.9, 2.5 and 5 K for **2**·0.5MeCN with applied fields 0–5 T. The measured magnetic moments have been corrected for the diamagnetic contribution of the Teflon tape and straw (-2.7×10^{-6} emu) and of the sample using Pascal's constants.[7] No correction was applied for temperature independent paramagnetism (TIP). The DC and magnetization data was modeled using the *curry* function on EasySpin.[8]

Torque Magnetometry

The cantilever torque measurements on a single crystal of **1** and **2** on a Quantum Design PPMS using a homemade two-leg CuBe cantilever separated by 0.1 mm from a gold plate. The crystal was secured on an acetate plate with Apiezon N-grease and indexed crystallographically using a Bruker D8 Venture diffractometer equipped with a PHOTON II detector and a microfocus source (Cu Kα radiation, λ = 1.54178 Å). Instrument calibration was verified before each run. The PPMS magnet was confirmed to provide highly homogeneous fields. We assume that the

crystal sample size is smaller than the region in which the field is homogeneous. Measurements were performed such that the CuBe cantilever is always operated strictly within its certified linear regime, taking care of measuring torque below 1×10^{-5} Nm to avoid deviations and to remove nonlinearity-related effects, per Quantum Design certification. The measured temperature deviated 0.001-0.013 K from the nominal set point and had stability ±0.002-0.008 K across the full measurement. The field stability was maintained at ±1 mT. The possibility that crystal shape anisotropy significantly affects the torque signals for weakly anisotropic paramagnetic samples such as **1** and **2** is likely negligible. Shape anisotropy could contribute measurably to torque in ferromagnetic or strongly magnetized systems; however, such magnetic behavior is incompatible with the properties required for spin qubits and is not observed in the present compounds. We also considered whether anisotropic temperature-independent paramagnetism (TIP) could contribute appreciably to the observed CTM signals. The TIP term for **1** and **2** is approximately 2×10^{-4} cm^3 mol^{-1}. This value is much smaller in comparison to the molar susceptibility (χ_M) across the full temperature range studied: $\chi_M \approx 0.5$ cm^3 mol^{-1} at 1.9 K, ≈ 0.01 cm^3 mol^{-1} at 100 K, and $\approx 4 \times 10^{-3}$ cm^3 mol^{-1} at 300 K. Therefore, any fractional component from anisotropic TIP is too small to meaningfully influence the measured torque response. The torque measurements were simulated using a home-written program partially based on EasySpin.[8]

For compound **2**, which contains lattice acetonitrile, we assume no loss of solvent during the measurement process. To minimize this risk, crystals are removed from the mother liquor immediately prior to CTM analysis, coating crystals with low-temperature-compatible grease, collecting the X-ray structure on the crystal used for CTM to resolve the solvent occupancy, and swiftly transferring the crystal to the magnetometer after indexing, where they are cooled to the lowest measurement temperature. These precautions help preserve the lattice solvent and ensure accurate molar mass determination.

X- and W-band Electron Paramagnetic Spectroscopy

X-band ($v \cong 9.40$ GHz) CW-EPR spectra were recorded for **1** (powder) on a Bruker Elexsys E500 spectrometer equipped with an ER4122SHQE resonator for perpendicular polarization and a ER4116DM resonator for parallel polarization. Low temperature measurements were obtained using an Oxford Instruments ESR900 continuous flow helium cryostat and temperature controlled by an Oxford Instruments ITC503. The EPR spectra for **1** was simulated using EasySpin.[8] For the simulation in perpendicular and parallel mode, a strain of E of 0.001 cm^{-1} for both modes were used. In general, EasySpin describes EPR line broadening using a two-parameter pseudo-Voigtian lineshape, specified as [Gaussian Lorentzian]. The two values correspond to the full width at half maximum (FWHM) of the Gaussian and Lorentzian contributions, respectively. In the present simulations, linewidths are given in units of MHz. Gaussian and Lorentzian broadening of 20 MHz and 29 MHz, respectively, was used for parallel mode simulation. Gaussian and Lorentzian broadening of 3 MHz and 60 MHz, respectively, was used for perpendicular mode simulation.

W-band ($v \cong 94$ GHz) CW-EPR spectra were recorded for **1** (single crystal) on a Bruker E600 continuous-wave spectrometer with cylindrical cavity equipped with a split-coil superconducting magnet that generates a horizontal magnetic field (Oxford Instruments). Temperature was controlled with a continuous-flow cryostat (Oxford CF94), operating at 35 K. The crystal of **1** was indexed (using a Bruker D8 Venture diffractometer equipped with a PHOTON II detector and a microfocus source (Cu Kα radiation, $\lambda = 1.54178$ Å) and positioned on a crystal cube of NaCl such that the magnetic field was parallel to $-c^*$ and placed on a 0.8 mm diameter quartz rod to perform the measurement. The EPR spectra was simulated using EasySpin.[8]

High-Frequency Electron Paramagnetic Spectroscopy

Multi-frequency HF-EPR spectra for **1** and **2** were recorded on ground microcrystalline powder samples (**1** was also recorded on a non-ground crystalline sample) pressed into pellets to reduce torquing effects. For **1**, the pellet was prepared in a N_2 atmosphere glovebox and wrapped using Teflon tape to protect from moisture. HF-EPR spectra were recorded with a multi-frequency spectrometer operating in a double-pass configuration.[9] Frequencies were provided either by a 127.68 or a 110.4 GHz frequency source (Virginia Diodes Inc.) associated to multipliers up to the third harmonic. The detection is performed with a hot electron InSb bolometer (QMC Instruments). The main magnetic field is supplied by a 16 T superconducting magnet equipped with a VTI (Cryogenic). Compound **1** was measured at 127.68 GHz at 5–125 K and 255.36 GHz at 5–15 K. Compound **2** was measured at 220.8, 255.36, and 331.2 GHz at 5–15 K. For each HF-EPR frequency, we recorded experimental spectra sweeping the magnetic field upfield and the other one downfield (with each different temperature measurement). Due to the large self-inductance of the superconducting magnet, an artificial hysteresis appears between these two spectra. We corrected the raw data by averaging the resonance of the sharp double quantum transition peak. The double quantum transition occurs at approximate fields of: **1** – 4.2 T (128 GHz) and 8.5 T (255 GHz), **2** – 6.7 T (221 GHz), 8 T (255 GHz) and 10.6 T (331 GHz). The double quantum transition is a transtion that occurs at the same field/frequency for the $m_s = -1 \rightarrow 0$ and $m_s = 0 \rightarrow +1$, which results in specific power dependence and exceptionally small linewidth.[10,11] The HF-EPR spectra for **1** and **2** were simulated using EasySpin.[8] For the simulation of **1**, phase corrections were applied individually to each spectrum (Table S1). For the simulation of the HF-EPR spectra of **1** and **2**, different Gaussian and Lorentzian broadening was utilized for the lower field (forbidden transition) and higher field regions (Table S2 and S3). For **1**, the two regions were defined by a separation at 5 T for the 255.36 GHz data and at

2.5 T for the 127.68 GHz data. For **2**. The two regions were defined by a separation at 5.5 T for the 331.2 GHz data, 4 T for the 255.36 GHz data, and 3.2 T for the 220.8 GHz data.

Table S1. Phase corrections used in simulations of HF-EPR spectra of **1**.

255 GHz, 5 K	255 GHz, 15 K					
π/8	π/5					
128 GHz, 5 K	128 GHz, 15 K	128 GHz, 30 K	128 GHz, 50 K	128 GHz, 70 K	128 GHz, 100 K	128 GHz, 125 K
14π/3	11π/10	π/4	10π/3	10π/3	10π/3	10π/3

Table S2. Gaussian and Lorentzian broadening parameters used in the EasySpin simulations of the HF-EPR data for **1**. EPR line broadening is described using a two-parameter Voigt lineshape, specified as [Gaussian, Lorentzian], where the two values correspond to the full widths at half maximum (FWHM) of the Gaussian and Lorentzian contributions, respectively, expressed in MHz.

	331 GHz, 5 K	331 GHz, 15 K	255 GHz, 5 K	255 GHz, 15 K	221 GHz, 5 K	221 GHz, 15 K
Low Field	[20 40]	[20 60]	[40 90]	[60 60]	[50 100]	[50 90]
High Field	[80 100]	[80 100]	[120 120]	[100 100]	[80 100]	[80 100]

Table S3. Gaussian and Lorentzian broadening parameters used in the EasySpin simulations of the HF-EPR data for **2**. EPR line broadening is described using a two-parameter Voigt lineshape, specified as [Gaussian Lorentzian], where the two values correspond to the full widths at half maximum (FWHM) of the Gaussian and Lorentzian contributions, respectively, expressed in MHz.

	255 GHz, 5 K	255 GHz, 15 K					
Low Field	[20 20]	[20 20]					
High Field	[50 50]	[20 60]					
	128 GHz, 5 K	128 GHz, 15 K	128 GHz, 30 K	128 GHz, 50 K	128 GHz, 70 K	128 GHz, 100 K	128 GHz, 125 K
Low Field	[20 30]	[20 30]	[20 20]	[20 20]	[20 20]	[20 20]	[20 20]
High Field	[30 80]	[50 60]	[20 70]	[20 60]	[20 70]	[20 70]	[20 70]

Ab Initio calculations

All the calculations have been performed with Orca 5.0.1 software.[12,13] Simulations have been performed on X-ray and DFT-optimized structures. The Douglas–Kroll–Hess (DKH) Hamiltonian for scalar relativistic effects has been employed. For all the atoms, the DKH-def2-TZVP basis set has been used.[14] DFT optimizations and simulations have been done with PBE0 functional.[15] All optimized geometries were ensured as local minima through frequency analysis, with no imaginary modes obtained in the resulting vibrational spectra. Wavefunction-based calculations were conducted at the complete active space self-consistent field (CASSCF) level of theory.[16] The active space comprised 8 electrons in the 3d orbitals of the Ni^{2+} ion, denoted as CAS(8,5). State-average calculations were performed to include all 10 triplet states, followed by time-dependent second-order N-electron valence perturbation theory (NEVPT2)[17,18] and spin-orbit perturbation. The *g*- and ZFS tensors have been mapped on the ground triplet wavefunction, as implemented in Orca.[19]

We calculated the spin Hamiltonian parameters of **1** and **2** with both DFT (PBE0) and CASSCF/NEVPT2 (Table S7). DFT reproduced the *g* values better than CASSCF/NEVPT2 (Table S7), and the main axes' directions of the *g*-tensors are almost colinear within the two methods. However, the *g* and *D* magnetic reference frames for both **1** and **2** are not colinear when calculated with DFT but are with CASSCF/NEVPT2. The positions of zero torque (and therefore Euler angles) in the CTM measurements of **1** and **2** do not vary significantly between high- and low-temperature. As the low-temperature data should be primarily influenced by ZFS and the high-temperature data by *g*, the minimal difference in Euler angles across the entire temperature range would suggest that the *g* and *D* magnetic reference frames are colinear. Fitting the CTM data for **1** and **2**, assuming non-colinearity, yielded essentially the same Euler angles for *g* and *D*. We conclude that CASSCF/NEVPT2 is overall more reliable in these systems with respect to DFT.

Table S4. Crystallographic data and structure refinement parameters for compound **2**·MeCN

	**2**·MeCN (100 K)	**2**·MeCN (293 K)
CCDC deposition number	2468331	2502207
Empirical formula	$C_{32}H_{29}F_{12}N_7NiP_2$	$C_{32}H_{29}F_{12}N_7NiP_2$
Formula weight	860.27	860.27
Temperature (K)	100.0(1)	293(2)
Crystal system	triclinic	triclinic
Space group	*P*-1	*P*-1
a (Å)	11.3157(2)	11.5276(13)
b (Å)	11.9773(3)	12.1985(13)
c (Å)	15.2245(3)	15.3765(16)
α (°)	93.3640(10)	93.923(5)
β (°)	109.5730(10)	109.377(4)
γ (°)	115.9900(10)	116.232(5)
Volume (Å^3)	1695.34(6)	1769.6(3)
Z	2	2
ρ_{calc} (g/cm^3)	1.685	1.615
μ (mm^{-1})	2.666	2.555
F(000)	872.0	872.0
Crystal size (mm^3)	0.78 × 0.623 × 0.205	0.78 × 0.61 × 0.2
Radiation	CuKα (λ = 1.54178)	CuKα (λ = 1.54184)
2θ range for data collection (°)	6.352 to 137.11	6.3 to 137.398
Index ranges	$-13 \le h \le 13, -14 \le k \le 14, -16 \le l \le 18$	$13 \le h \le 13, -14 \le k \le 14, -17 \le l \le 18$
Reflections collected	45032	53292
Independent reflections	6220 [R_{int} = 0.0449, R_{sigma} = 0.0283]	6489 [R_{int} = 0.0514, R_{sigma} = 0.0339]
Data/restraints/parameters	6220/0/488	6489/21/544
Goodness-of-fit on F^2	1.048	1.028
Final *R* indexes [$I \geq 2\sigma (I)$]	R_1 = 0.0298, wR_2 = 0.0774	R_1 = 0.0398, wR_2 = 0.1065
Final *R* indexes [all data]	R_1 = 0.0306, wR_2 = 0.0780	R_1 = 0.0419, wR_2 = 0.1087
Largest diff. peak/hole (e Å^{-3})	0.52/-0.47	0.35/-0.28

Table S5. Octahedral distortion parameters for **1** and **2**.

	Oh *SHAPE* [a]	Σ / ° [b]	Θ / ° [b]	∠N_1−Ni−N_2 / °	∠N_2−Ni−N_5 / °	∠N_5−Ni−N_6 / °	∠N_1−Ni−N_6 / °	∠N_1−Ni−N_3 / °	∠N_2−Ni−N_3 / °	∠N_3−Ni−N_5 / °	∠N_3−Ni−N_6 / °	∠N_1−Ni−N_4 / °	∠N_2−Ni−N_4 / °	∠N_4−Ni−N_5 / °	∠N_4−Ni−N_6 / °
**1**	0.62	59	165	82.97	95.06	87.56	94.40	81.49	88.56	98.54	89.46	80.96	91.85	99.09	89.32
**2**	2.78	117	388	81.40	95.62	79.51	105.85	79.36	106.77	94.37	93.41	81.61	79.63	105.39	82.58

[a] *SHAPE* index for octahedral geometry in SHAPE 2.1.[20,21] A value of 0 represents a perfect octahedron. [b] Σ = sum of the deviation of the 12 N−Ni−N angles from 90°. Θ = sum of the deviation of 24 unique torsional angles between the N atoms on opposite triangular faces of the octahedron from 60°, providing the degree of trigona1 distortion from an octahedron to trigonal prism. These were calculated using OctaDist - a program for determining the structural distortion of the octahedral complexes. For a perfect octahedron, Σ and Θ are zero.[22]

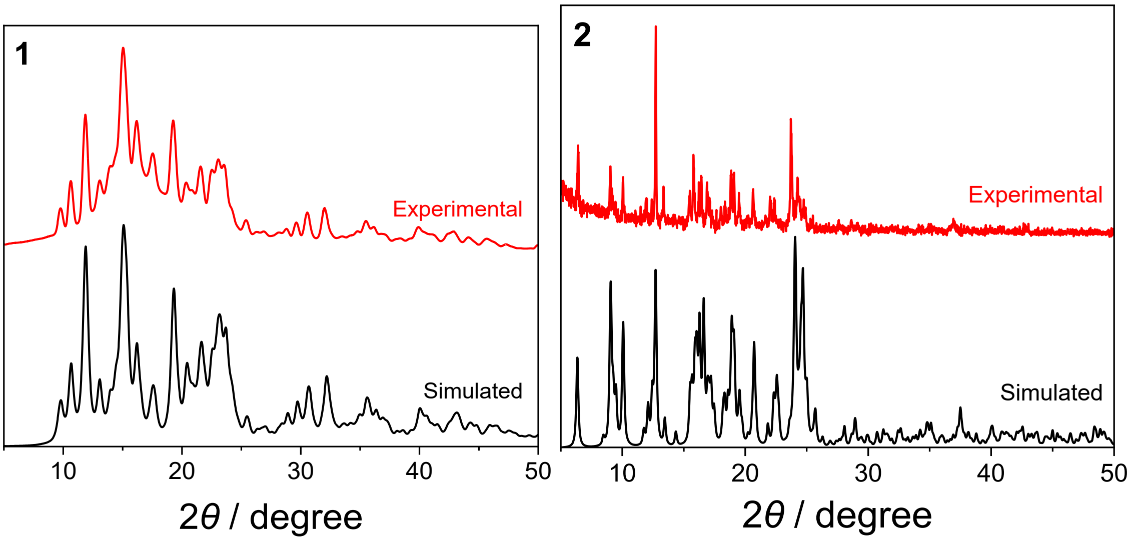

Figure S1. Left: Experimental PXRD pattern of **1** at 300 K (red) and simulated PXRD pattern from crystal structure of **1** at 100 K (black). Right: Experimental PXRD pattern of **2**·0.5MeCN at 300 K (red) and simulated PXRD pattern from crystal structure of **2**·0.5MeCN at 100 K.

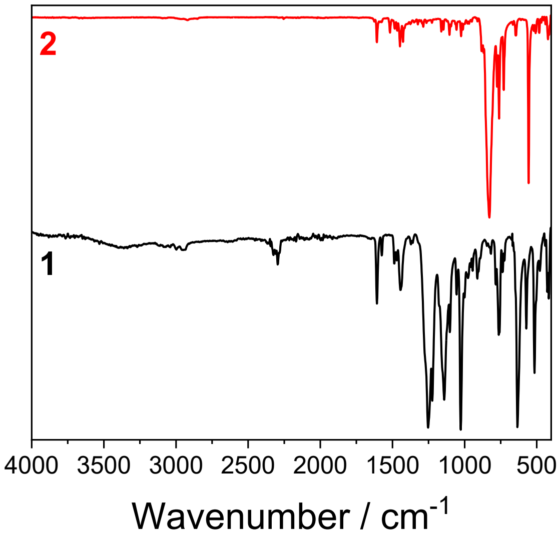

Figure S2. ATR-IR spectra of **1** and **2**.

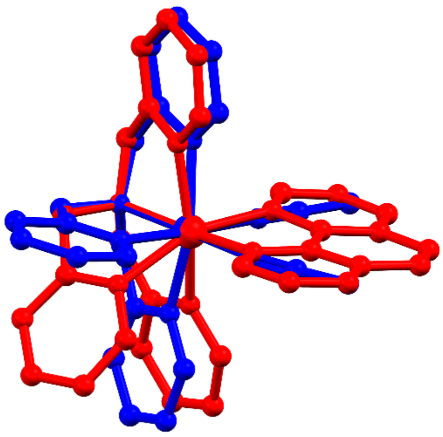

Figure S3. Overlay of the cationic structures of **1** (blue) and **2** (red) determined by X-ray diffraction at 100 K. Hydrogen and counterions omitted.

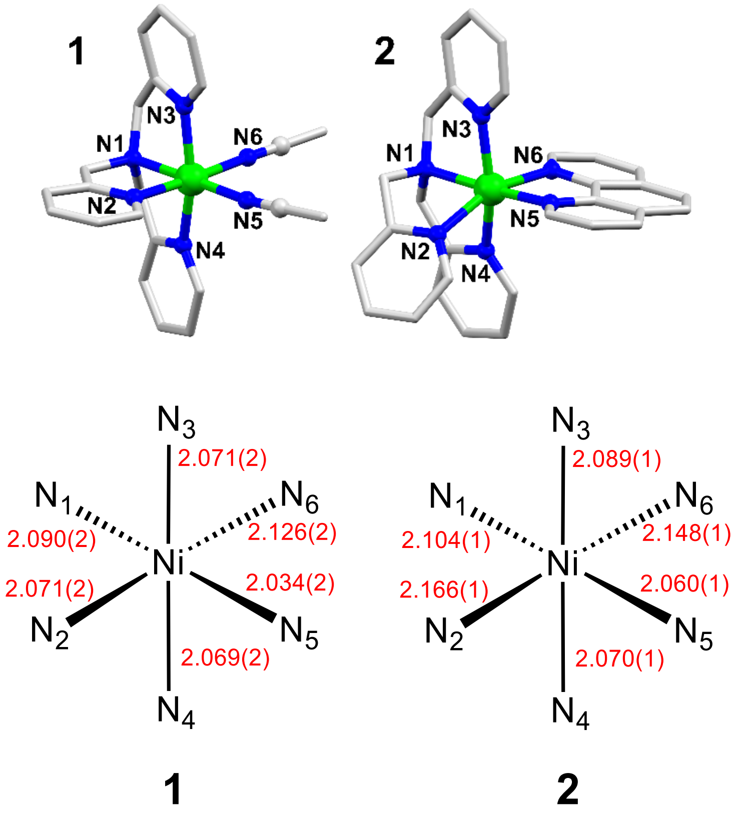

Figure S4 Ni–N bond lengths for compounds **1** and **2**·MeCN at 100 K. The N_2/N_6 bond axis is elongated relative to the other two bond axes.

Optical Measurements and Ligand Field Analysis

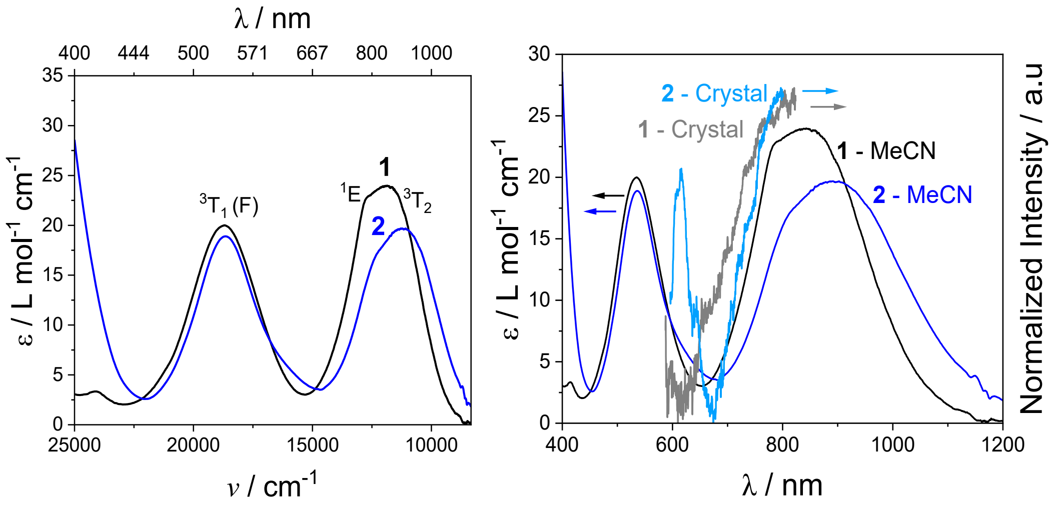

Figure S5. Left: UV-Vis absorption spectra of **1** and **2** in MeCN at 298 K. Right: UV-Vis absorption spectra of **1** and **2** in MeCN at 298 K overlaid with single-crystal absorption spectra of **1** and **2** at 3.5 K.

UV-Visible absorption spectra of **1** and **2** in MeCN at room temperature was used to determine the ligand field splitting energy, Δo (10*Dq*). Octahedral Ni(II) (d^8) complexes have a $^3A_{2g}$ ground state, giving rise to three spin allowed d-d transitions, in increasing energy $^3A_{2g} \rightarrow {}^3T_{2g}$ (F), $^3A_{2g} \rightarrow {}^3T_{1g}$ (F), $^3A_{2g} \rightarrow {}^3T_{1g}$ (P), with the former corresponding to Δo. For **1**, $^3A_{2g} \rightarrow {}^3T_{2g}$ (F) is observed at 11920 cm^{-1} and $^3A_{2g} \rightarrow {}^3T_{1g}$ (F) is observed at 18710 cm^{-1}, with the third band obscured by ligand-based transitions. A high energy shoulder at 12840 cm^{-1} is observed on the $^3A_{2g} \rightarrow {}^3T_{2g}$ (F) transition, which corresponds to the spin forbidden $^3A_{2g} \rightarrow {}^1E_g$,[23–25] which gains intensity through SOC with $^3T_{2g}$.[24,25] For **2**, $^3A_{2g} \rightarrow {}^3T_{2g}$ (F) is observed at 11,270 cm^{-1} and $^3A_{2g} \rightarrow {}^3T_{1g}$ (F) is observed at 18660 cm^{-1}, with the third band obscured by ligand-based transitions. A high energy shoulder is observed at 12420 cm^{-1} on the $^3A_{2g} \rightarrow {}^3T_{2g}$ (F) transition, which corresponds to the spin forbidden $^3A_{2g} \rightarrow {}^1E_g$.[23–25] Therefore, it appears that

Δo is 11900 and 11270 cm^{-1} for **1** and **2**, respectively. However, when Δo is close to 12000 cm^{-1}, mixing of the $^3A_{2g} \rightarrow {}^3T_{2g}$ (F) and $^3A_{2g} \rightarrow {}^1E_g$ bands becomes significant, complicating the analysis of Δo.[26] The following empirical relationship was developed to deal with this issue:[26]

$10Dq$ (corrected) = 10630 + 1370(ε1/ε2)

where ε1/ε2 is the ratio of the extinction coefficients of the lower to higher wavelength components. This results in a corrected value for Δo of 11900 and 11780 cm^{-1} for **1** and **2** respectively.

The Racah parameter B (covalency parameter) can be calculated with:[27]

$$15\mathrm{B} = \frac{(\sigma_2 - 2\Delta_o)(\sigma_2 - \Delta_o)}{(\sigma_2 - 1.8\Delta_o)}$$

where $\sigma_1 = E(^3A_{2g} \rightarrow {}^3T_{2g})$ and $\sigma_2 = E(^3A_2 \rightarrow {}^3T_{1g}(F))$. This yields B = 853 and 883 cm^{-1} for **1** and **2**, respectively. The free ion Racah B parameter of Ni(II) is 1041 cm^{-1},[28,29] resulting in a β = 0.82 and 0.85 for **1** and **2**, respectively.

The higher energy $^3A_{2g} \rightarrow {}^3T_{1g}$ (P) (σ_3) band can be calculated using:[27,30]

$$\sigma_3 = \frac{15B}{2} + \frac{3\Delta_o}{2} + \frac{1}{2}\sqrt{(9B - \Delta_o)^2 + 144B^2}$$

This gives an approximate value of 29780 and 29930 cm^{-1} for **1** and **2**, respectively.

We used quantum chemical calculations to study the energy of the excited triplet and singlet states of **1** and **2** (Figure S6, Table S6). *Ab initio* CASSCF/NEVPT2[16–18] calculations were used to estimate the energies of the interconfigurational excited triplet state (lowest microstates of $^3T_2$ and $^3T_1$ origin) and singlet state (lowest microstates of ^1E origin) levels. The relative energy ordering of the triplet and singlet excited states for both compounds are captured correctly; i.e the lowest excited state is $^3T_2$ with ^1E being higher in energy (Figure S6,

Table S6). The calculated energies of the $^3T_2$, ^1E and $^3T_1$ states are consistently higher for **1** compared to **2**, reflecting experimental observations and emphasising the increased ligand field strength in **1** compared to **2**. The calculations slightly overestimate the energy of the ^1E state compared to experimental observations but otherwise capture the trends well (Figure S6, Table S6). Importantly, the degeneracies of the $^3T_2$ and $^3T_1$ levels are more significantly lifted in **2** than in **1** (Figure S6), cconsistent with its larger deviation from ideal octahedral geometry (Figure 1).[24] In contrast, the splitting of the ^1E level is comparatively modest. (Figure S6).

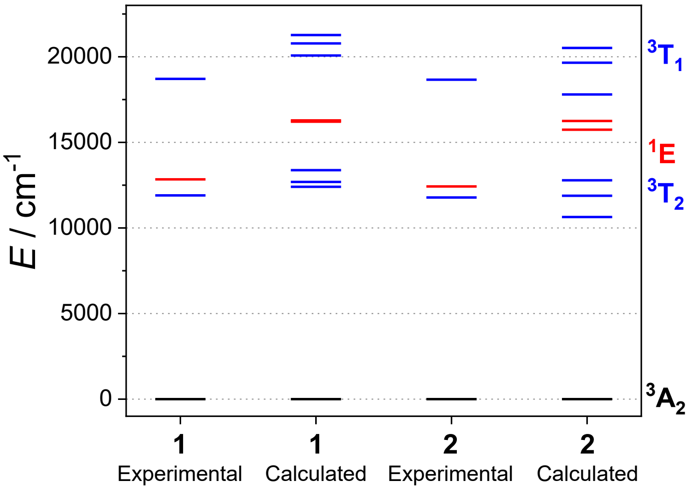

Figure S6. Energies of the lowest triplet (blue) and singlet (red) states of **1** and **2** at the ground-state geometry as determined experimentally or from CASSCF/NEVPT2 calculations.

Table S6. Tabulated energies of the lowest triplet (blue) and singlet (red) states of **1** and **2** at the ground-state geometry as determined experimentally or from CASSCF/NEVPT2 calculations.

	$^3T_{2g}$ / cm^{-1}	^1E_g / cm^{-1}	$^3T_{1g}$ (F) / cm^{-1}	$^3T_{1g}$ (P) / cm^{-1}
**1** (experimental)	11900	12840	18710	29780
**1** (calculated)	12400	16200	20070	33000
**2** (experimental)	11780	12420	18660	29930
**2** (calculated)	10630	15740	17790	31010

Table S7. Calculated spin Hamiltonian parameters for **1** and **2** using DFT and CASSCF/NEVPT2.

	g_x	g_y	g_z	g_{iso}	$\lvert g_{hard}-g_{intermediate}\rvert$	$\lvert g_{easy}-g_{intermediate}\rvert$	$\lvert g_{easy}-g_{hard}\rvert$	$\lvert g_x-g_y\rvert/\lvert g_{easy}-g_{hard}\rvert$	D / cm^{-1}	E / cm^{-1}	E/D
DFT											
**1**	2.1579	2.1604	2.1481	2.155	0.010	0.003	0.012	0.21	0.872	0.223	0.26
**2**	2.1473	2.1609	2.1766	2.162	0.014	0.016	0.029	0.47	−1.856	0.377	0.20
CASSCF/NEVPT2 without inclusion of singlet excited states											
**1**	2.1938	2.2007	2.1812	2.192	0.013	0.007	0.020	0.35	2.645	0.709	0.27
**2**	2.1833	2.2074	2.2381	2.210	0.024	0.031	0.055	0.44	−7.091	1.910	0.27
CASSCF/NEVPT2 with inclusion of singlet excited states											
**1**	2.1836	2.1897	2.1712	2.182	0.012	0.006	0.019	0.32	1.961	0.549	0.28
**2**	2.1727	2.1957	2.2245	2.198	0.023	0.029	0.052	0.44	−5.660	1.585	0.28
Experimental CTM (final fit)											
**1**	2.1481	2.1628	2.1175	2.1428	0.031	0.016	0.046	0.34	1.80	0.152	0.08
**2**	2.076	2.130	2.182	2.129	0.054	0.052	0.106	0.51	−3.895	1.264	0.32
Experimental HF-EPR											
**1**	2.155	2.155	2.14	2.15	0.015	0	0.015	0	1.55	0.16	0.10
**2**	2.14	2.16	2.21	2.17	0.02	0.05	0.07	0.29	−4.25	1.416	0.33

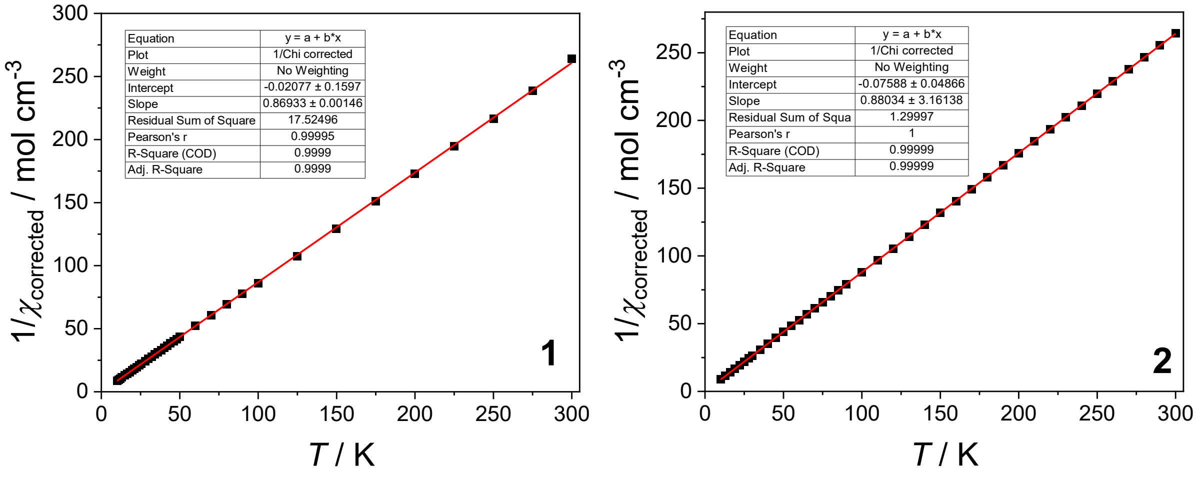

Figure S7. Plot of $1/\chi_{\text{corrected}}$ vs T to show linear regime between 10-300 K, confirming Curie Weiss behavior. $\chi_{\text{corrected}}$ represents a correction of χ from TIP, with the values of 2.1×10^{-4} and 1.5×10^{-4} $\text{cm}^3\ \text{mol}^{-1}$ for **1** and **2**, respectively.

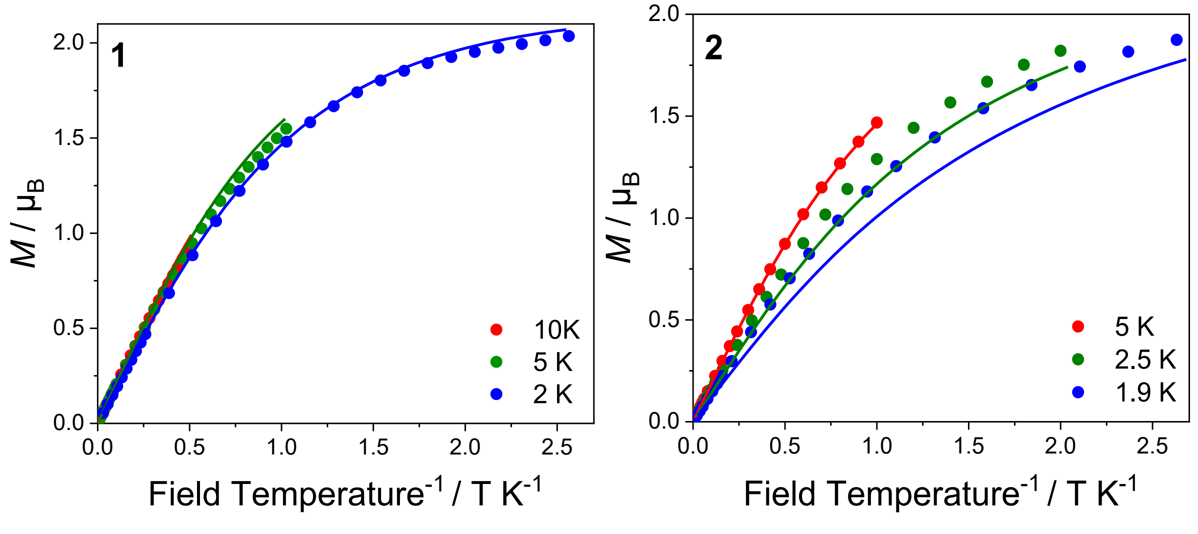

Figure S8. Plot of magnetization vs field temperature^{-1} for **1** and **2** at specified temperatures (dots). Solid lines are simulations using CASSCF/NEVPT2 calculated parameters (Table 1): **1**: g_x =2.1836, g_y = 2.1897, g_z = 2.1712, D = 1.96 cm^{-1}, E = 0.55 cm^{-1}. **2**: g_x = 2.1727, g_y = 2.1957, g_z = 2.2245, D = −5.66 cm^{-1}, E = 1.59 cm^{-1}

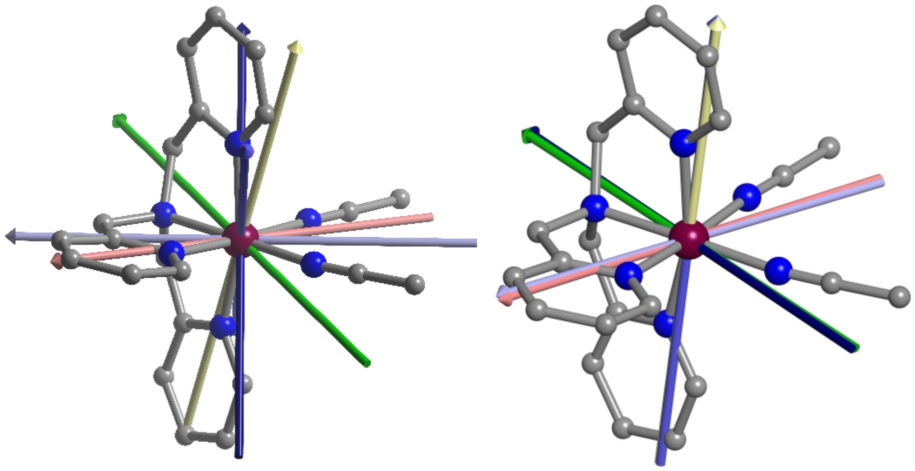

Figure S9. DFT (left) and CASSCF/NEVPT2 (right) calculated magnetic references (g_x (intermediate axis) in yellow, g_y (easiest axis) in green, and g_z (hard axis) in red) and ZFS reference frames (D_x (intermediate axis) blue, D_y (easiest axis) dark blue, and D_z (hard axis) light blue) of **1**.

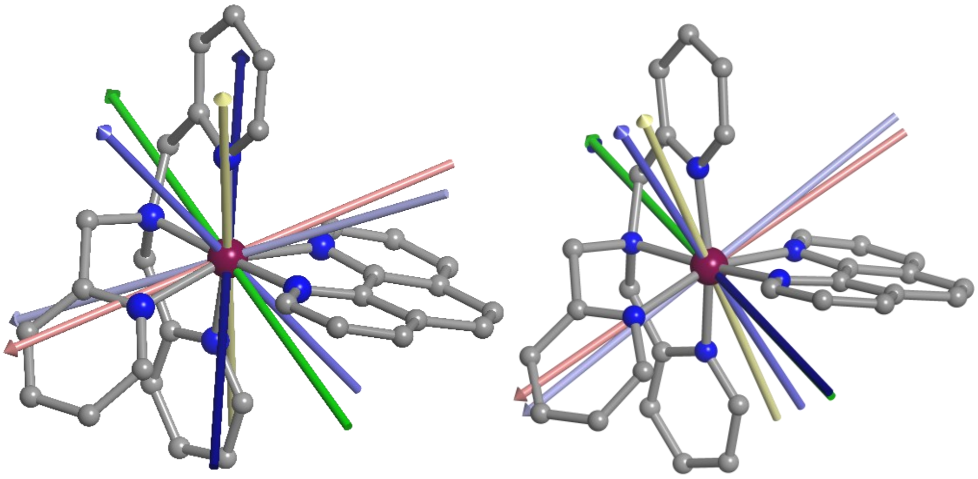

Figure S10. DFT (left) and CASSCF/NEVPT2 (right) calculated magnetic references (g_x (hard axis) in red, g_y (intermediate axis) in yellow, and g_z (easy axis) in green) and ZFS reference frames (D_x (hard axis) light blue, D_y (intermediate axis) blue, and D_z (easy axis) dark blue) of **2**.

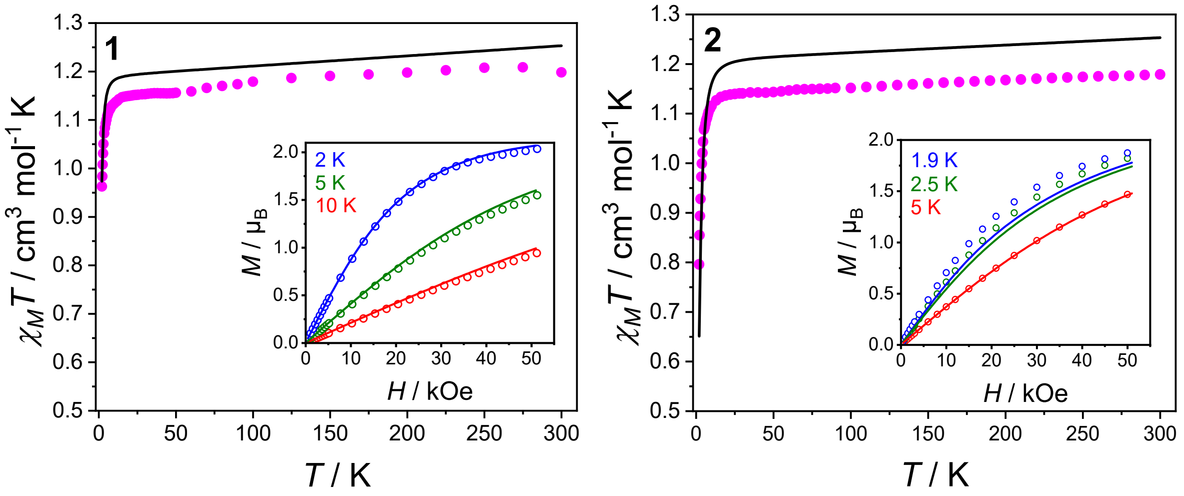

Figure S11. $\chi_M T$ vs T for **1** and **2**. Inset: magnetization vs field at three temperatures. Solid lines are simulations using CASSCF/NEVPT2 calculated parameters (Table 1) and inclusion of temperature-independent paramagnetism (TIP): **1**: g_x =2.1836, g_y = 2.1897, g_z = 2.1712, D = 1.96 cm^{-1}, E = 0.55 cm^{-1}, TIP = 2.1 × 10^{-4} cm^3 mol^{-1}. **2**: g_x = 2.1727 , g_y = 2.1957, g_z = 2.2245, D = −5.66 cm^{-1}, E = 1.59 cm^{-1}, TIP = 1.5 × 10^{-4} cm^3 mol^{-1}.

Cantilever Torque Magnetometry

The magnetic torque ($\boldsymbol{\tau}$) is the vector product between magnetization (**M**) and magnetic field (**B**). In the low-field/high-temperature regime, $\boldsymbol{\tau}$ depends linearly on the magnetic anisotropy of the susceptibility tensor.[31] Therefore, when an anisotropic paramagnet is rotated in a homogenous magnetic field, $\boldsymbol{\tau}$ vanishes every 90° (i.e whenever projection of main magnetic axis of sample in scanned plane is parallel or perpendicular to the applied field). For high fields and/or low temperatures, the angular dependence of $\boldsymbol{\tau}$ becomes less trivial, but the fundamental characteristic still is that $\boldsymbol{\tau}$ vanishes when the field is parallel (easy zero) and perpendicular (hard zero) to the projection of the easiest magnetization direction (lowest free energy direction) in the scanned crystallographic plane.

Compound **1** crystallizes in the monoclinic $P2_1/n$ space group with four molecules in the unit cell, with an inversion center reducing the number of magnetically equivalent molecules to two. Measurements were performed on three different rotations of the crystal. In the first rotation (Rot. 1), at $\theta = 0°$, the magnetic field is parallel to c^* and the rotation axis is a, with the projection of the magnetization in the bc^* plane detected. Due to the presence of a C_2 axis along the b axis of the cell (due to the monoclinic symmetry), τ vanishes when the field is parallel and perpendicular to b (i.e at 0° and 90°). In the second rotation (Rot. 2) at $\theta = 0°$, the magnetic field is parallel to a and the rotation axis is c^*. This means the scanned plane is ab, and likewise τ vanishes at 0° and 90°. In the third rotation (Rot. 3) at $\theta = 0°$, the magnetic field is parallel to c^* and the rotation axis is $-b$; therefore, no zeros are imposed by symmetry and are found at 33° and 123°.

Compound **2** crystallizes in the triclinic $P\bar{1}$ space group with the two molecules in the unit cell related by an inversion center, resulting in one magnetically inequivalent molecule. Measurements were performed on three different rotations of the crystal. Due to the lack of

symmetry in triclinic crystals, there are no preferential rotation planes for CTM. Therefore, rotations were selected to be along the three coordination axes of the complex in **2**. Rotation 1 scans the plane made up of N_{amine}, N_5 and N_6 of phen, and N_2, with rotation along N_3 and N_4 (Figure S12). Rotation 2 scans the plane of the three pyridines of tpa (N_2, N_3, N_4) and N_6 of phen, with the rotation axis along the N_{amine} N_5 phen axis (Figure S12). Rotation 3 contains N_{amine} and N_5 of phen, and N_3 and N_4, with rotation axis alone the N_2 and N_6 phen axis (Figure S12).

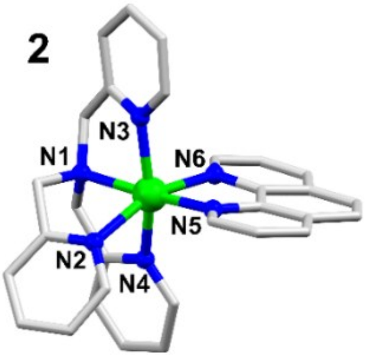

Figure S12. Molecular structure of $[Ni(tpa)(phen)]^{2+}$ with N-donor atoms labeled. Hydrogen atoms have been omitted for clarity. Colour code: C (grey), N (blue), Ni (green).

From CTM, the Euler angles (in degrees) determined for the magnetic tensors of **1** and **2** are [71.5, 67.8, 82.4] and [278.9, 138.7, 320.5], respectively, with respect to the ab'c* reference frame. For the analysis of how the ZFS magnitude affects *g* parameter precision, we used:

$$MAE = \frac{1}{n}\sum_{i=1}^{n}|g_i - \hat{g}_i| \qquad \text{(eq. S1)}$$

where n is number of data points, g_i is g value from high-temperature fit (for experimental CTM for **1** and **2**) or g_{set}, and $\hat{g}_i$ is the g value from global fit (for experimental CTM for **1** and **2**) or g_{fit}.

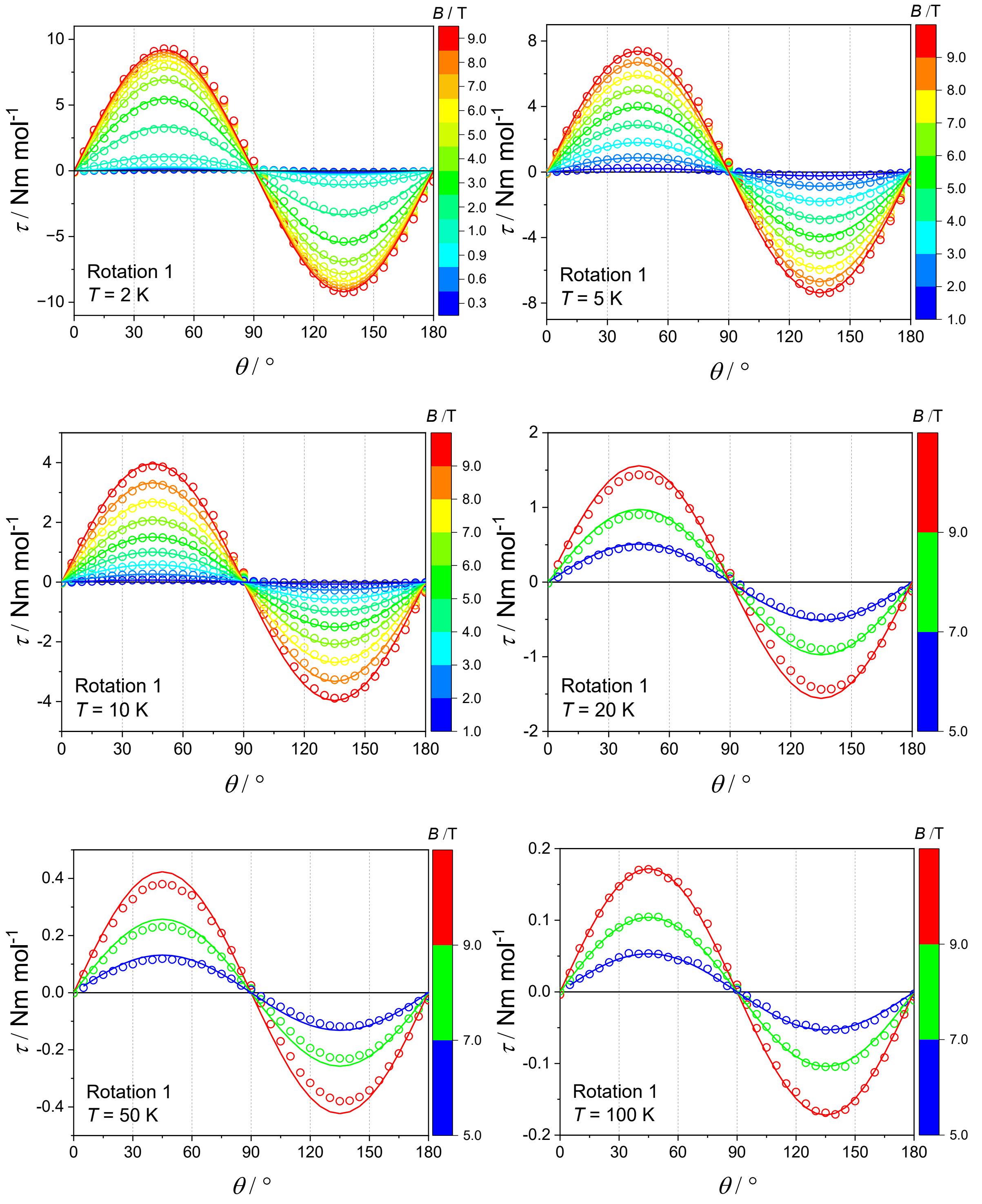

Figure S13. Experimental (circles) and simulated (line) torque curves for rotation 1 of **1** at 2, 5, 10, 20, 50 and 100 K at various fields.

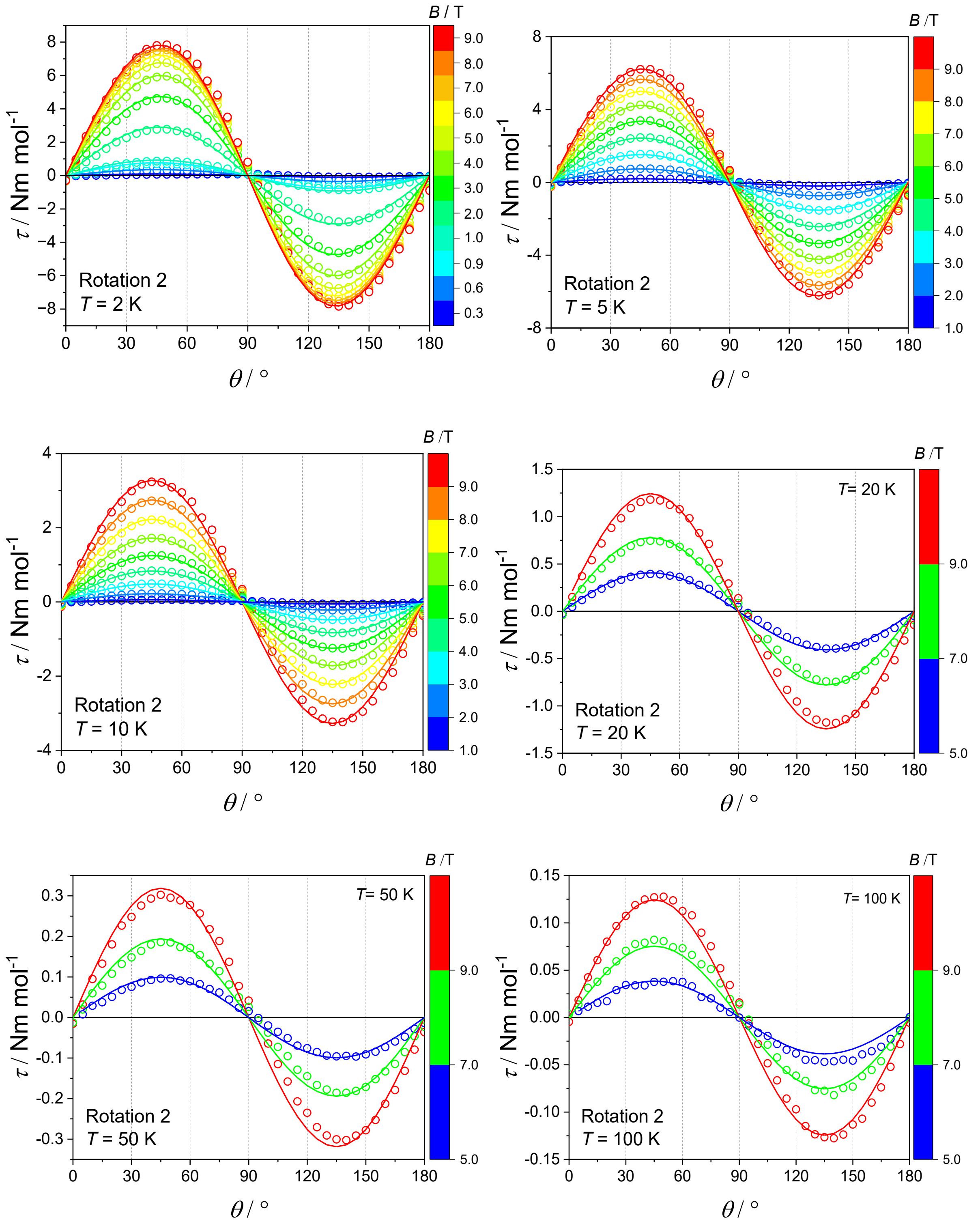

Figure S14. Experimental (circles) and simulated (line) torque curves for rotation 2 of **1** at 2, 5, 10, 20, 50 and 100 K at various fields.

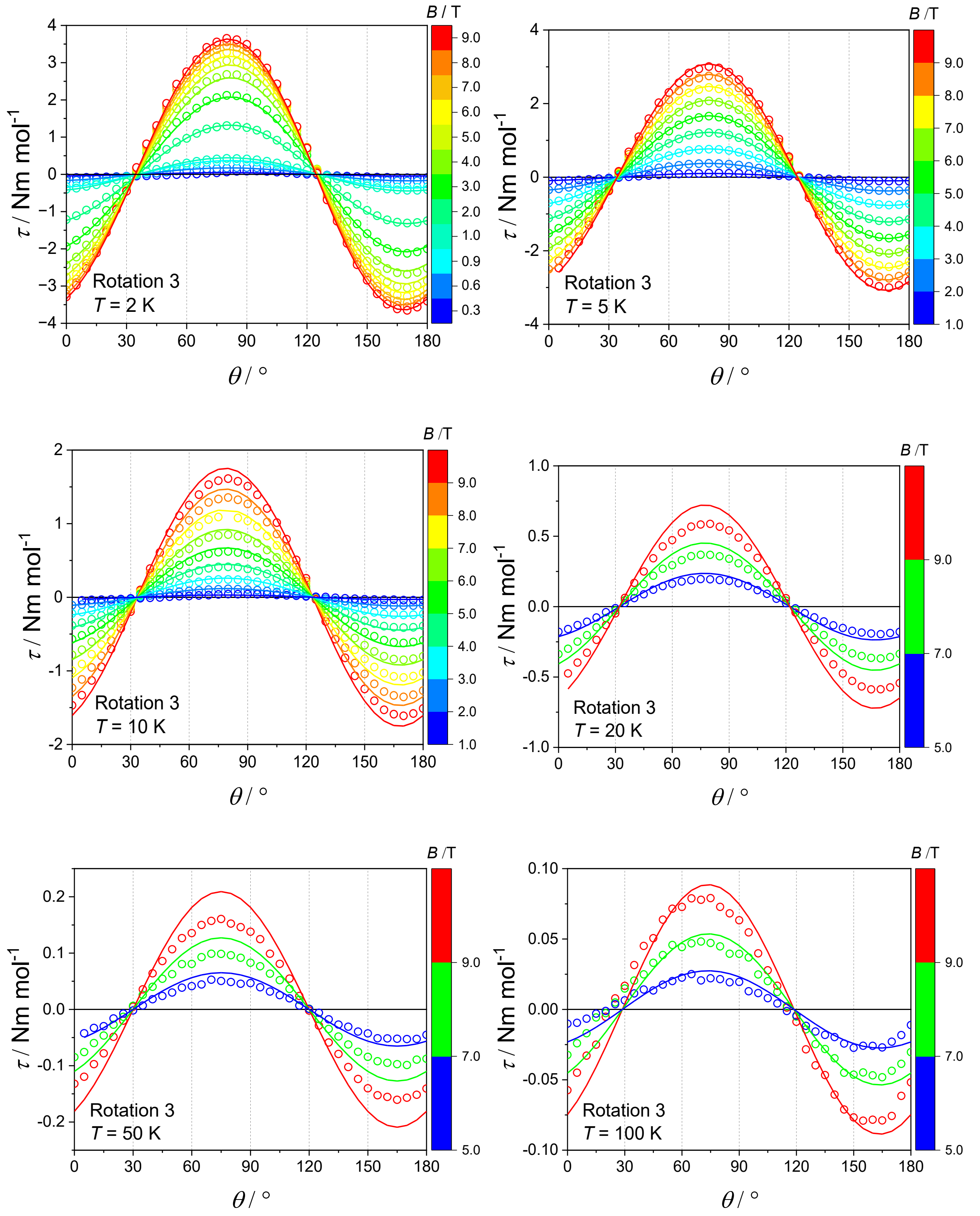

Figure S15. Experimental (circles) and simulated (line) torque curves for rotation 3 of **1** at 2, 5, 10, 20, 50 and 100 K at various fields.

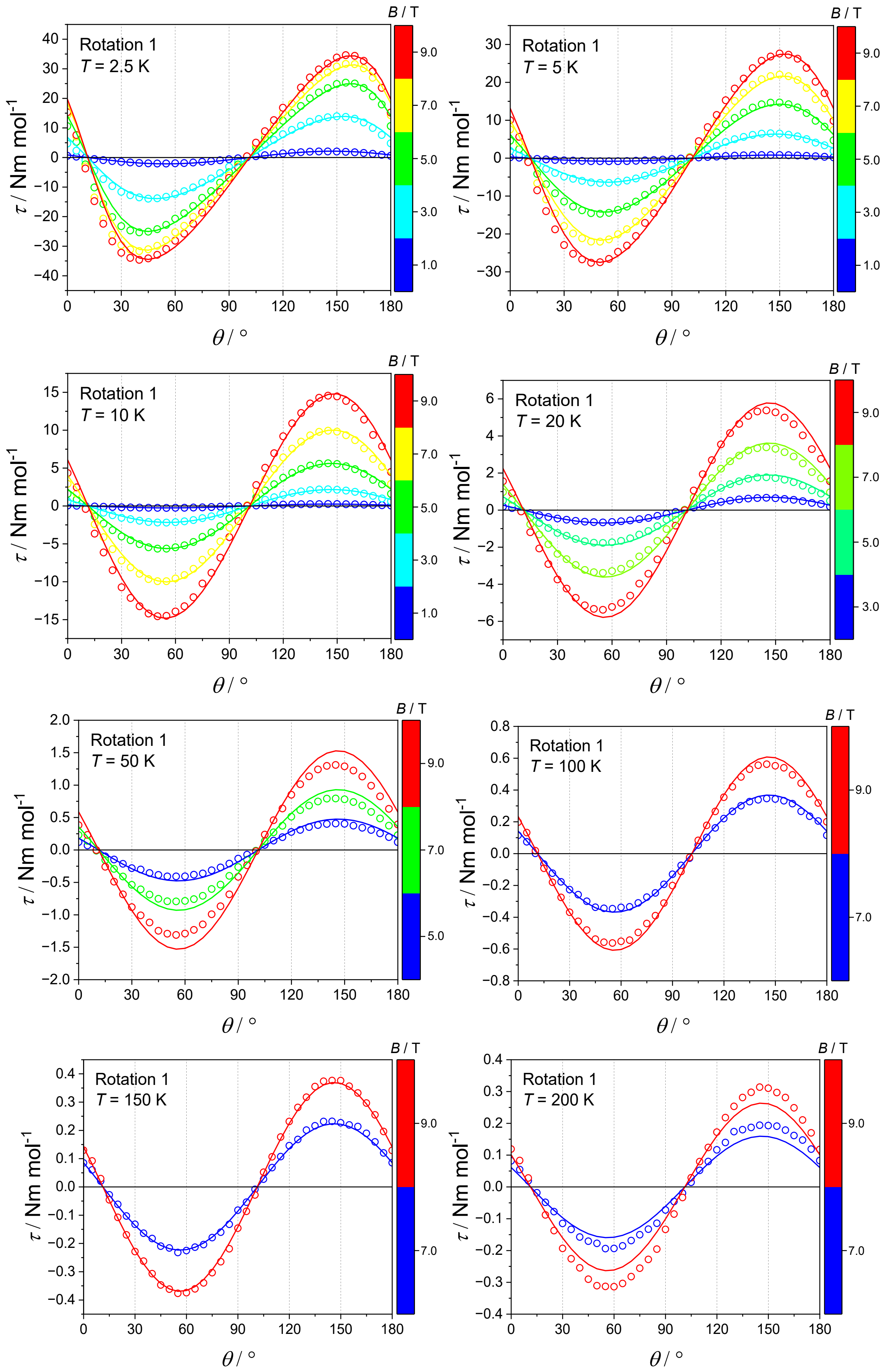

Figure S16. Experimental (circles) and simulated (line) torque curves for rotation 1 of **2** at 2.5, 5, 10, 20, 50, 100, 150 and 200 K at various fields.

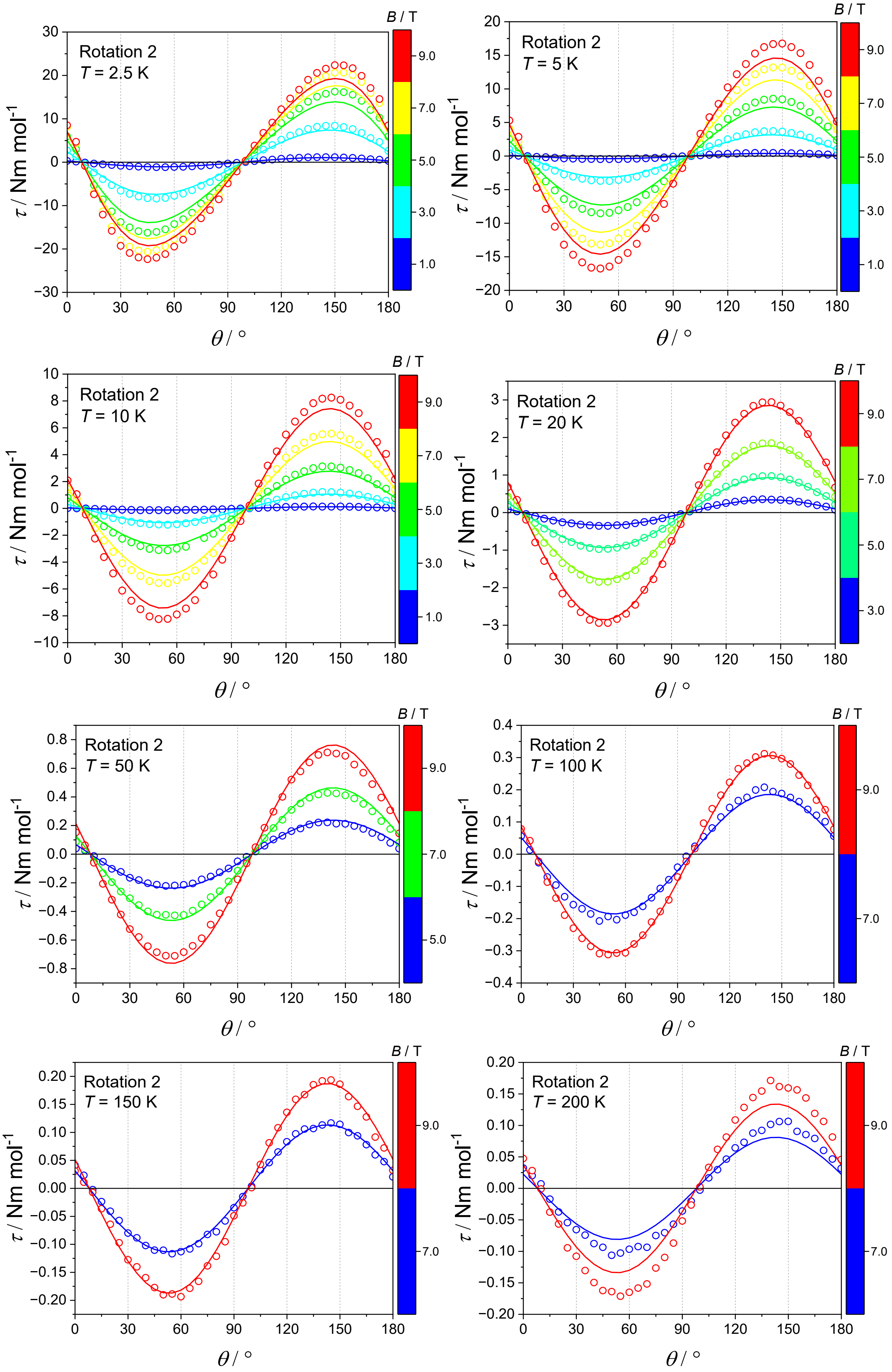

Figure S17. Experimental (circles) and simulated (line) torque curves for rotation 2 of **2** at 2.5, 5, 10, 20, 50, 100, 150 and 200 K at various fields.

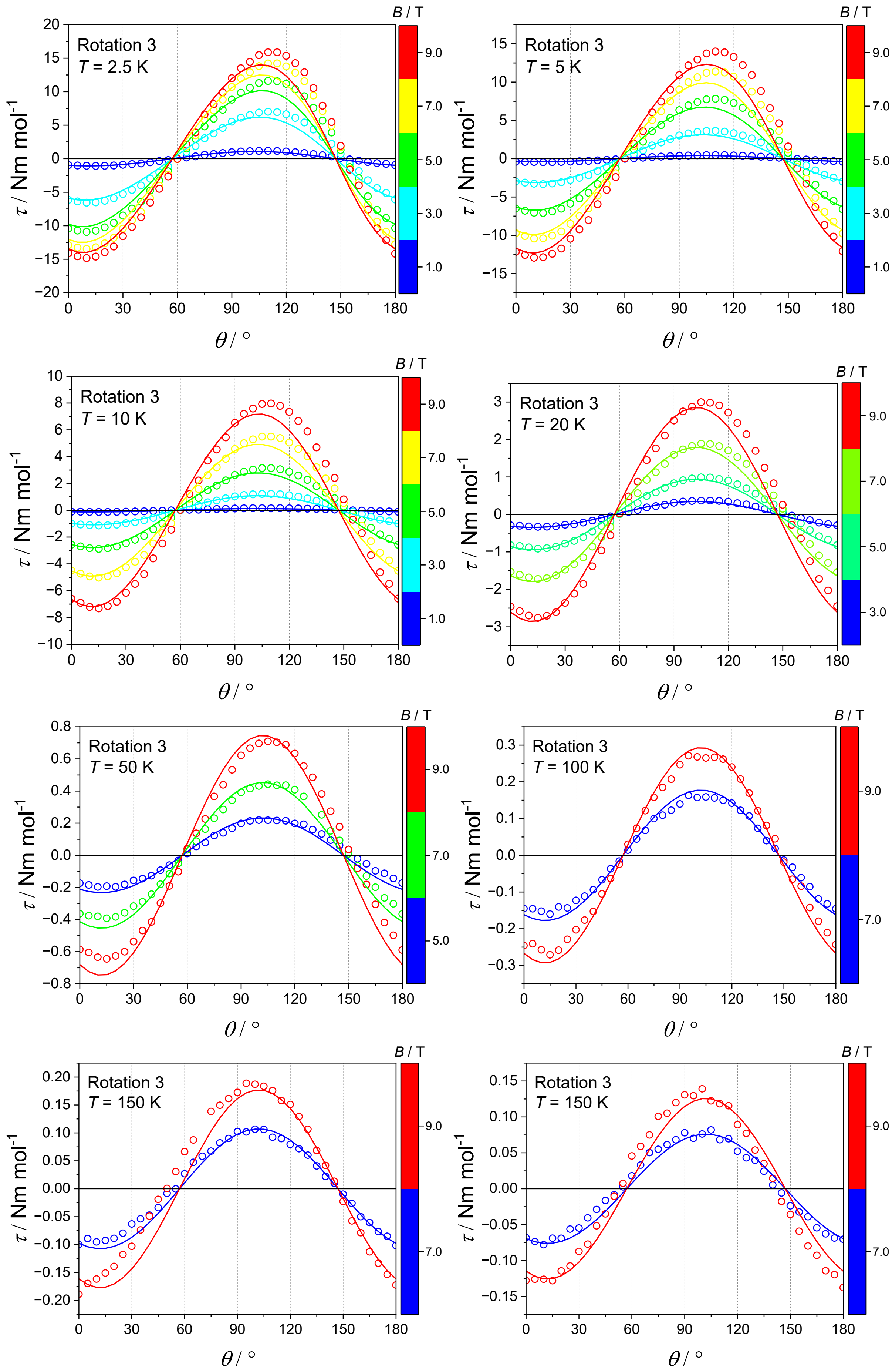

Figure S18. Experimental (circles) and simulated (line) torque curves for rotation 3 of **2** at 2.5, 5, 10, 20, 50, 100, 150 and 200 K at various fields.

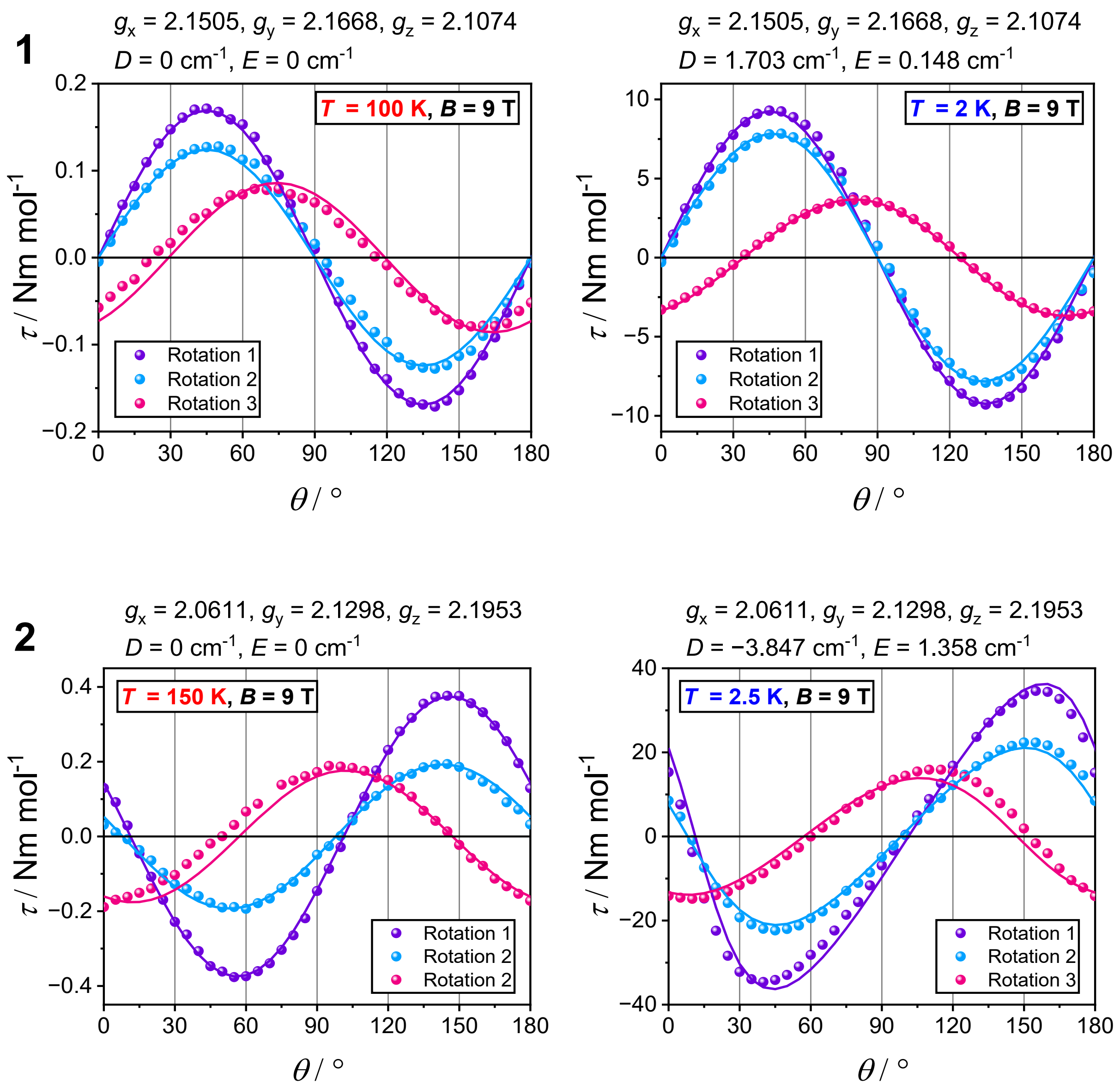

Figure S19. Top left: Experimental (circles) and simulated (line) torque curves for rotation 1, 2 and 3 for **1** at 100 K and 9 T with a fit for just *g* and Euler angles. Top right: Experimental (circles) and simulated (line) torque curves for rotation 1, 2 and 3 for **1** at 2 K and 9 T with a fit for just *D* and *E* with *g* and Euler angles fixed based on the values derived from the 100 K fit. Bottom left: Experimental (circles) and simulated (line) torque curves for rotation 1, 2 and 3 for **2** at 150 K and 9 T with a fit for just *g* and Euler angles. Bottom right: Experimental (circles) and simulated (line) torque curves for rotation 1, 2 and 3 for **2** at 2.5 K and 9 T with a fit for just *D* and *E* with *g* and Euler angles fixed based on the values derived from the 150 K fit.

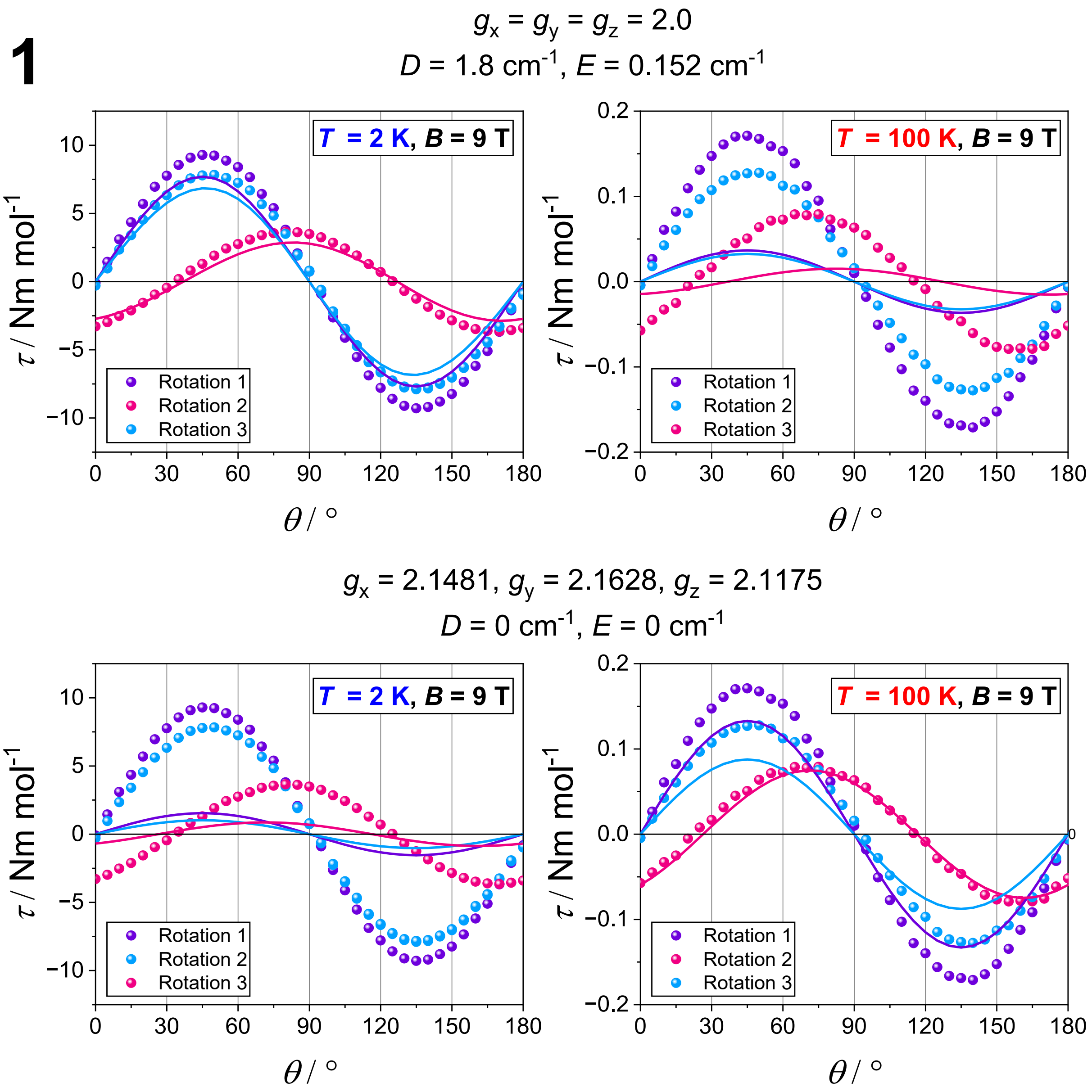

Figure S20. Torque signals for **1** at 2 and 100 K. Solid line represents simulated curves either with isotropic $g = 2$ and ZFS determined from the final CTM fit of **1** (top) or no ZFS and g values determined from the final CTM fit of **1** (bottom).

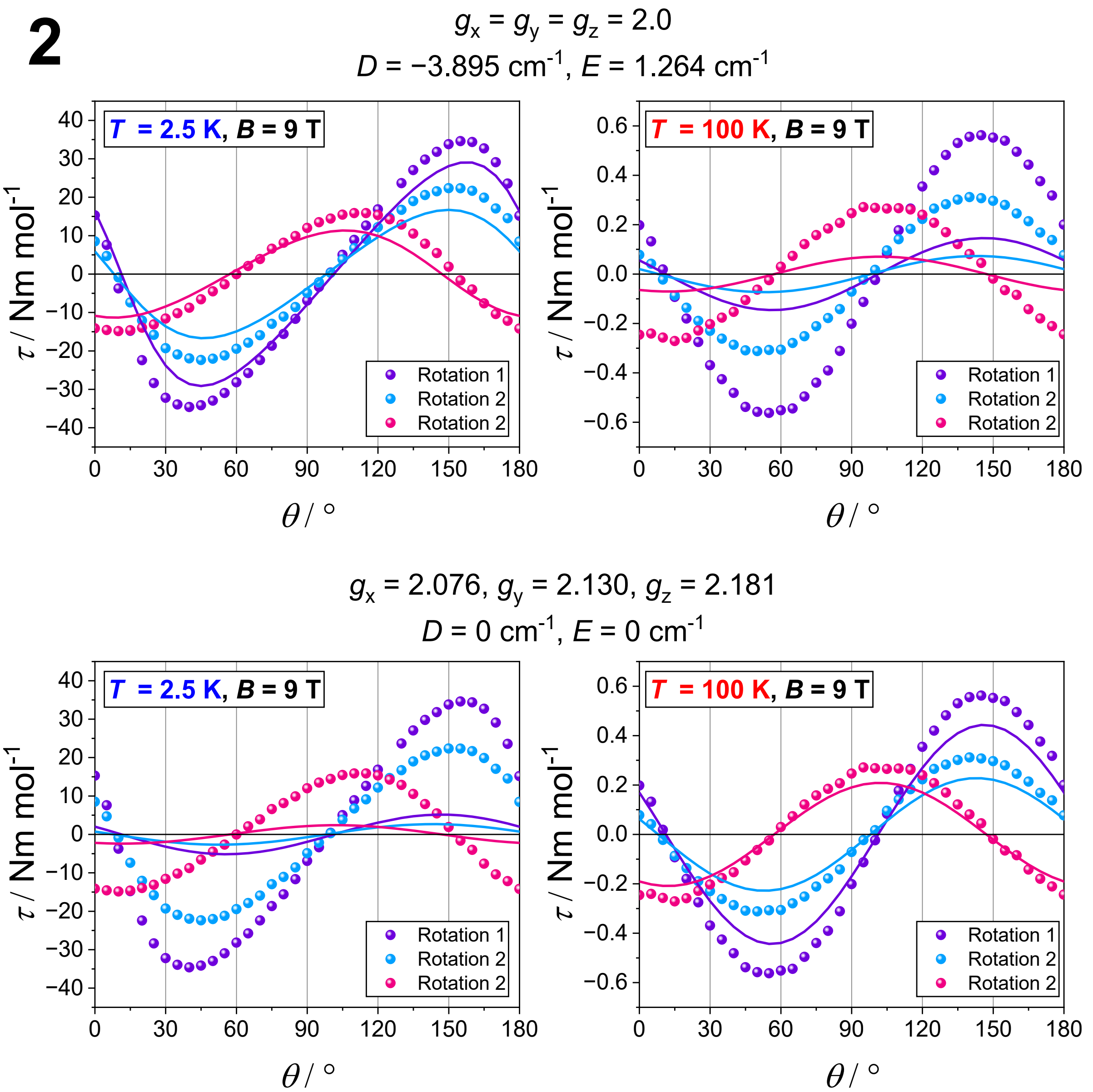

Figure S21. Torque signals for **2** at 2 and 100 K. Solid line represents simulated curves either with isotropic $g = 2$ and ZFS determined from the final CTM fit of **2** (top) or no ZFS and g values determined from the final CTM fit of **2** (bottom).

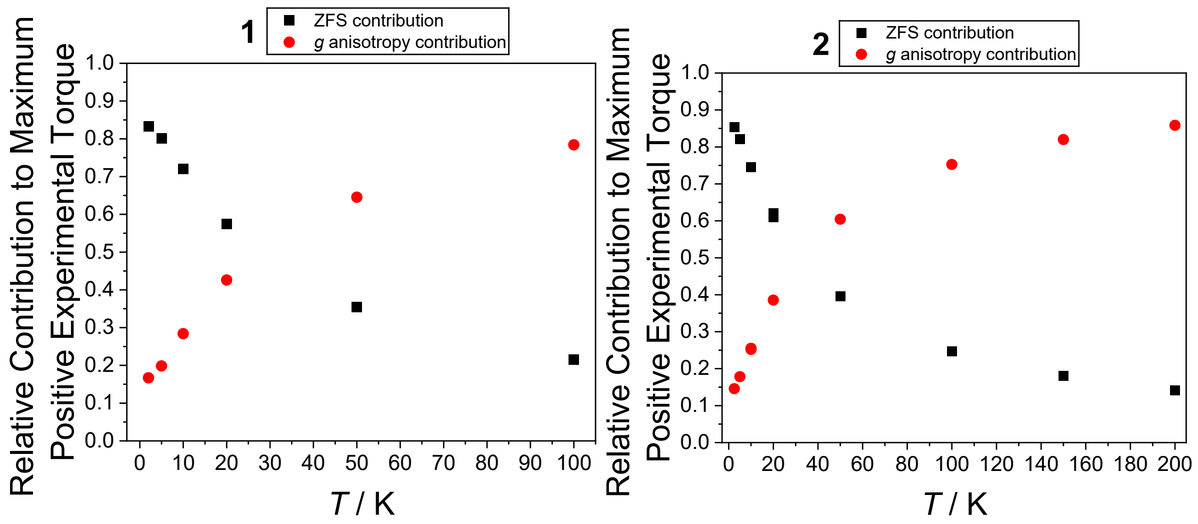

Figure S22. Normalized fractional contribution to the maximum experimental positive torque value (dots, Figure S20, S21) from only ZFS (no *g*-anisotropy, top panels of Figure S20, S21, lines) and *g*-anisotropy (no ZFS, bottom panels of Figure S20, S21, lines) across the full temperature range. The points represent the average data from the three rotations. This illustrates that as temperature increases, the relative contribution of *g*-anisotropy simulated torque curve increases.

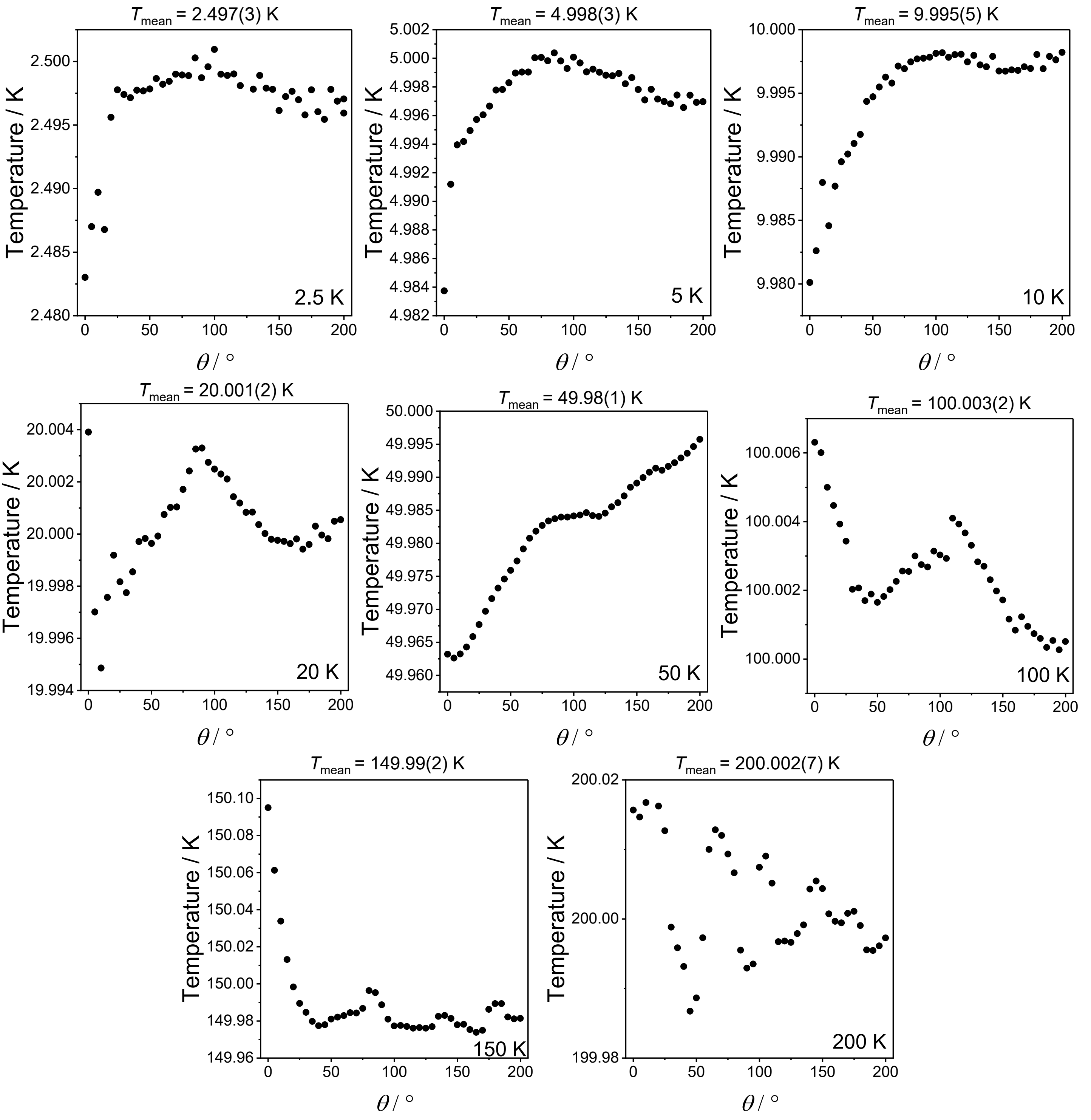

Figure S23. Representative example of the temperature recorded at each rotation angle during CTM measurement (rotation 1 at 9 T for compound **2**). Full analysis of the temperature across all fields and rotations for **2** is presented in Table S8.

Table S8. Across the full temperature range investigated (2.5–200 K), the temperature stability during data acquisition is within ±0.002–0.008 K, and the deviation of the mean measured temperature from the nominal set temperature lies within ±0.001–0.013 K. The measured temperature corresponds to the instrument sensor that is placed just below the sample plate reading. The actual temperature of the sample may be different to that provided by the instrument. In addition, calibration and thereby accuracy of the temperature measurement may introduce a systematic offset. Across the full temperature range investigated (2.5–200 K), the temperature stability during acquisition lies within ±0.002–0.008 K, and the deviation of the mean measured temperature from the nominal set point lies within ±0.001–0.013 K. The reported temperature corresponds to the instrument sensor located directly beneath the sample plate. The true sample temperature may differ slightly due to calibration offsets or thermal gradients, which would introduce a systematic shift affecting absolute accuracy but not relative measurement precision.

Set temperature point (Tset) / K	Mean measured temperature ($\bar{T}$) / K	Deviation of mean measured temperature with set temperature ($\Delta T = \bar{T} - T$set) / K	Standard deviation (SD)(stability) of the measured temperature / K	Means absolute deviation (MAD) from mean measured temperature / K
2.5	2.4958	0.0042	0.0047	0.0037
5	4.9989	0.0011	0.002	0.0016
10	10.0009	0.0009	0.0023	0.018
20	20.0004	0.0004	0.0034	0.0026
50	50.0035	0.0035	0.0056	0.0043
100	100.002	0.002	0.004	0.0032
150	149.9875	0.0125	0.0075	0.0068
200	200.0035	0.0035	0.008	0.0065

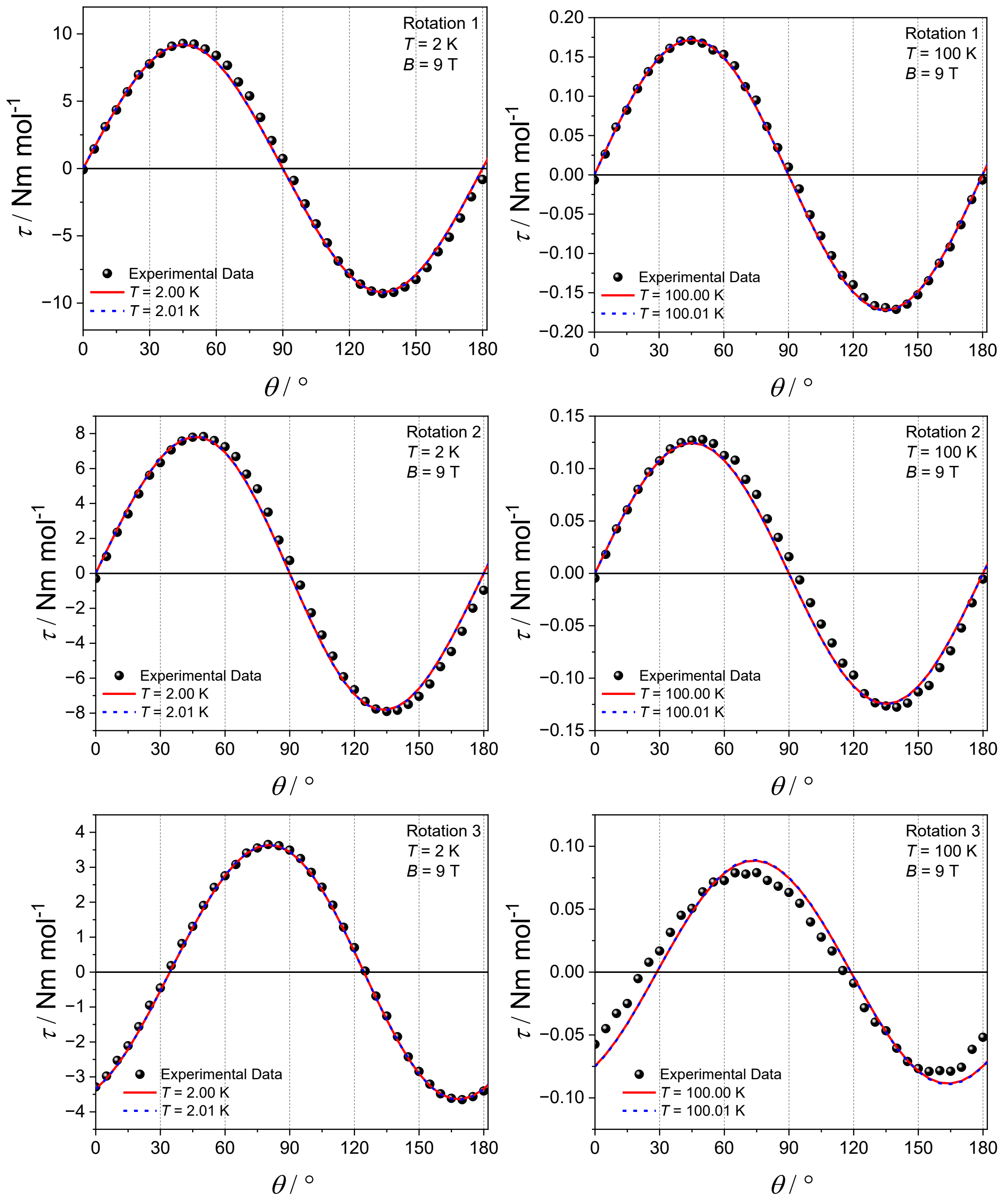

Figure S24. Experimental (circles) data for **1** and simulated (line) torque curves using the Spin Hamiltonian parameters determined from CTM (g_x =2.1481, g_y = 2.1628, g_z = 2.1175, D = 1.80 cm^{-1}, E = 0.152 cm^{-1}) with T = 2.00 or 100.00 K (red line), or T = 2.01 or 100.01 K (blue dots). Subsequent fitting of the data (all temperatures, fields, and rotations) with T = ±0.01 K resulted in: g_x =2.1481, g_y = 2.1629, g_z = 2.1173, D = 1.801 cm^{-1}, E = 0.1521 cm^{-1}.

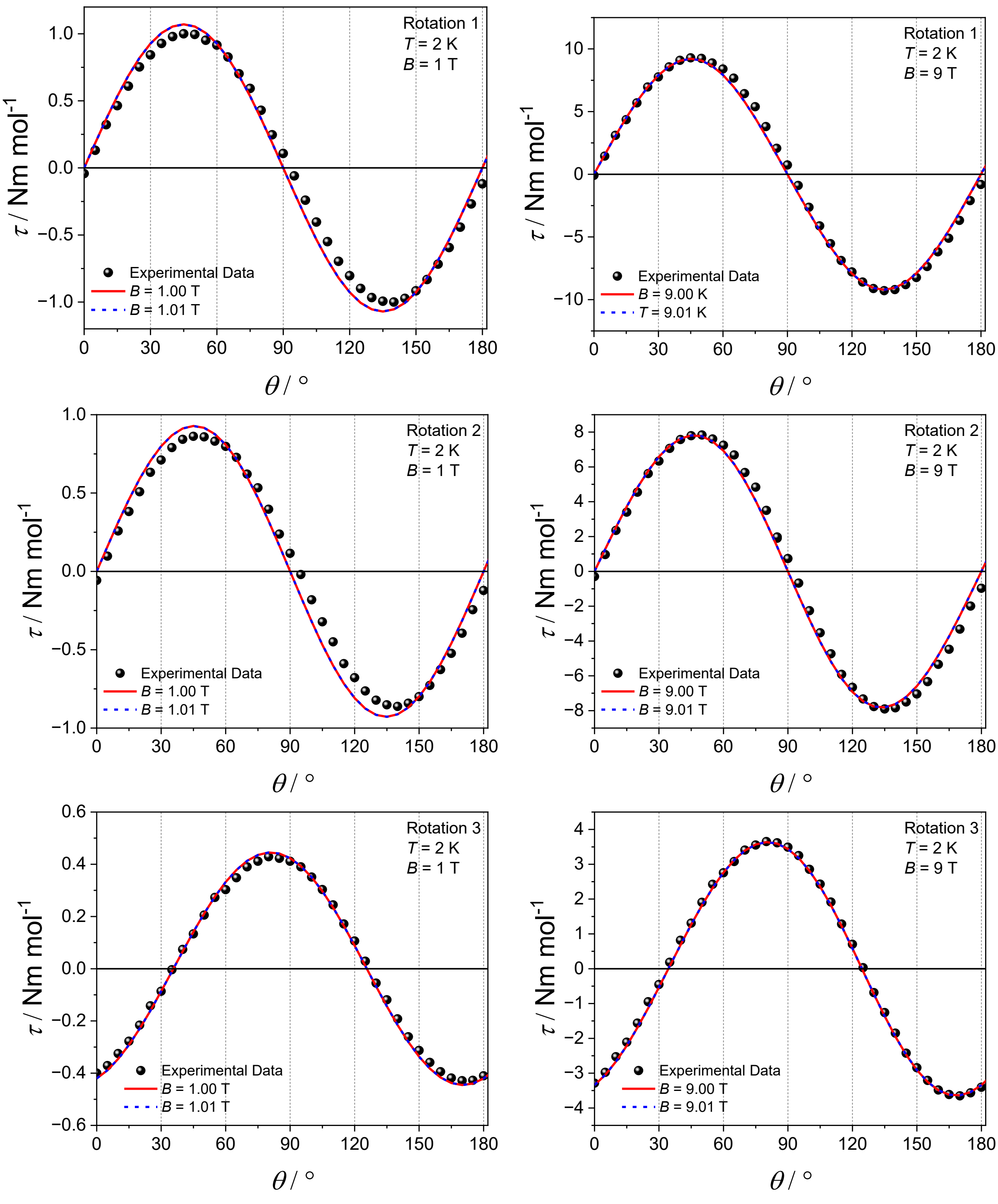

Figure S25. Experimental (circles) data for **1** and simulated (line) torque curves using the Spin Hamiltonian parameters determined from CTM (g_x =2.1481, g_y = 2.1628, g_z = 2.1175, D = 1.80 cm^{-1}, E = 0.152 cm^{-1}) with B = 1.00 or 9.00 T (red line), or B = 1.01 or 9.01 T (blue dots). Subsequent fitting of the data (all temperatures, fields, and rotations) with B= ±0.01 T resulted in: g_x =2.1481, g_y = 2.1628, g_z = 2.1175, D = 1.799 cm^{-1}, E = 0.1520 cm^{-1}

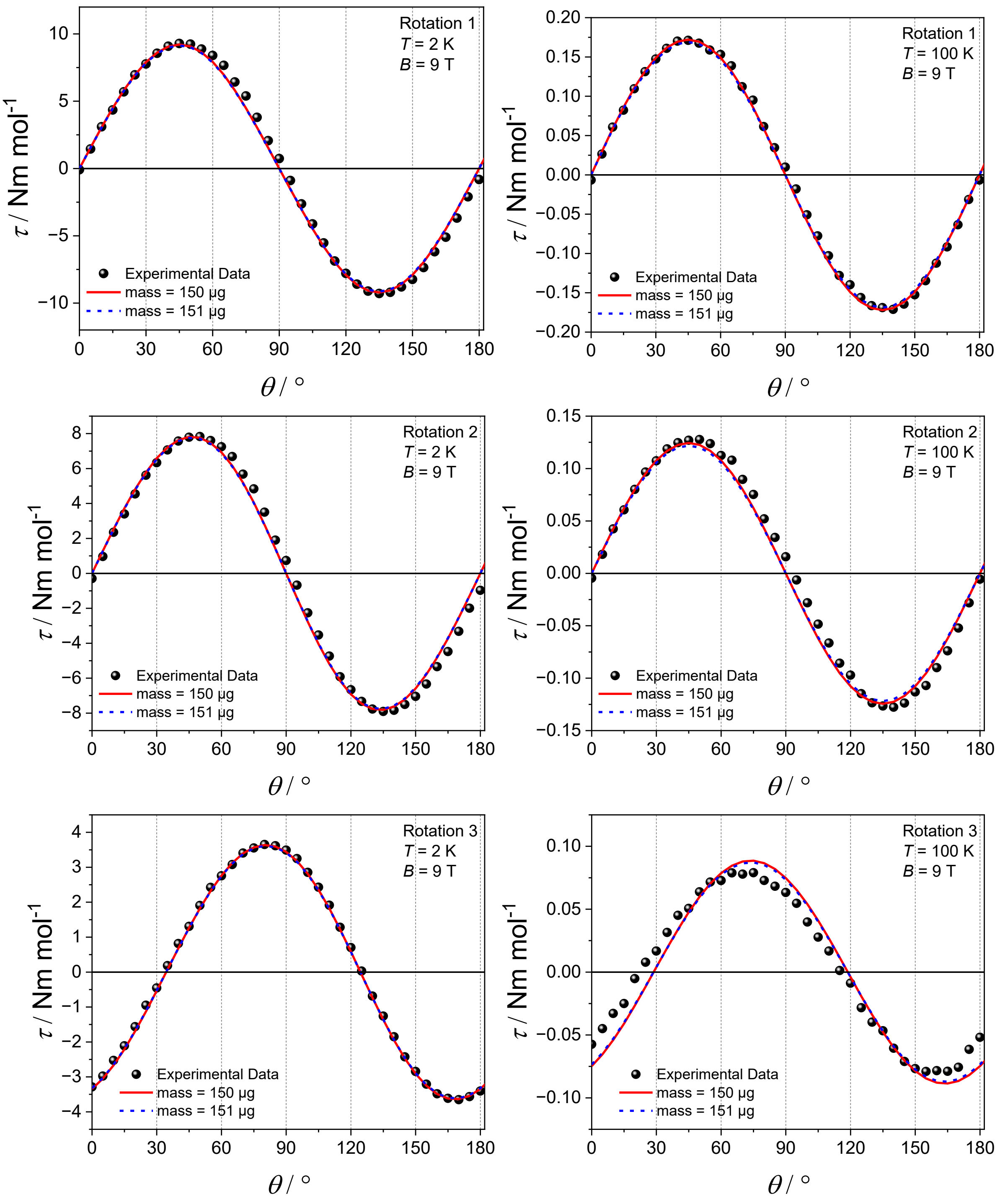

Figure S26. Experimental (circles) data for **1** and simulated (line) torque curves using the Spin Hamiltonian parameters determined from CTM (g_x =2.1481, g_y = 2.1628, g_z = 2.1175, D = 1.80 cm^{-1}, E = 0.152 cm^{-1}) with mass = 150 (red line) or 151 μg (blue dots). Subsequent fitting of the data (all temperatures, fields, and rotations) with mass = ±1 μg resulted in: g_x =2.1481, g_y = 2.1628, g_z = 2.1175, D = 1.796 cm^{-1}, E = 0.1530 cm^{-1}.

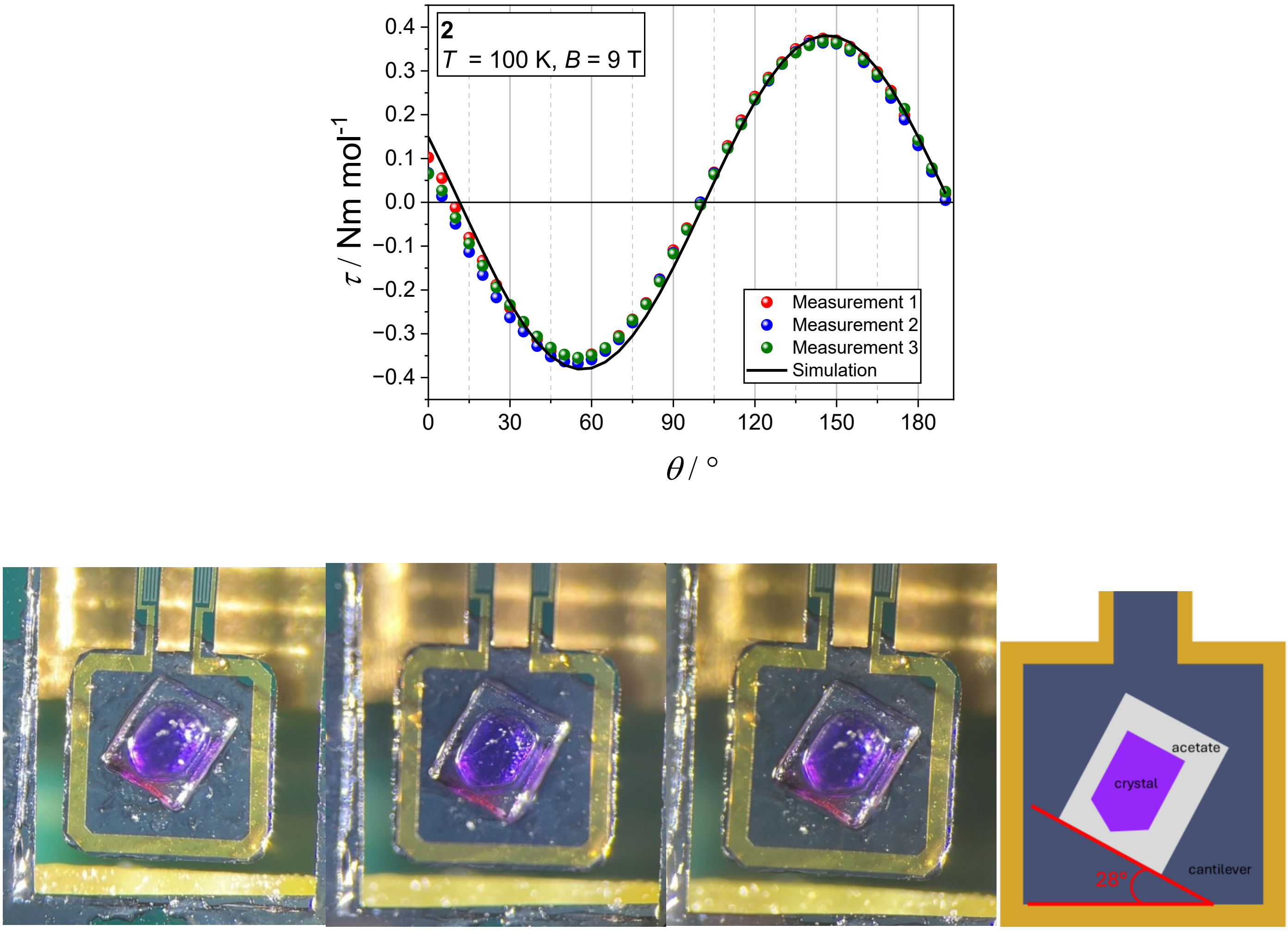

Figure S27. Torque curves for **2** measured at 100 K and 9 T to assess reproducibility with respect to manual crystal alignment. The crystal was mounted at an arbitrary but known orientation of approximately 28° relative to the cantilever (photographs taken down the microscope) three times. After each measurement, the crystal was removed and remounted (done twice), each time manually realigned to the same nominal 28° orientation by eye. The three resulting experimental torque traces (circles) overlap almost perfectly, demonstrating that small user-dependent variations in mounting angle produce only negligible and reproducible deviations in the measured torque. The solid line corresponds to the simulated torque curve at this same orientation, generated without any additional parameter adjustment using the final Spin Hamiltonian parameters and Euler angles obtained from the global CTM analysis (Table 1). The excellent agreement between simulation and all three independent measurements confirms (i) the robustness of the extracted parameters, (ii) the insensitivity of CTM to minor remounting variations, and (iii) that orientation-related uncertainties contribute minimally to the overall error budget under the stable, well-calibrated conditions employed.

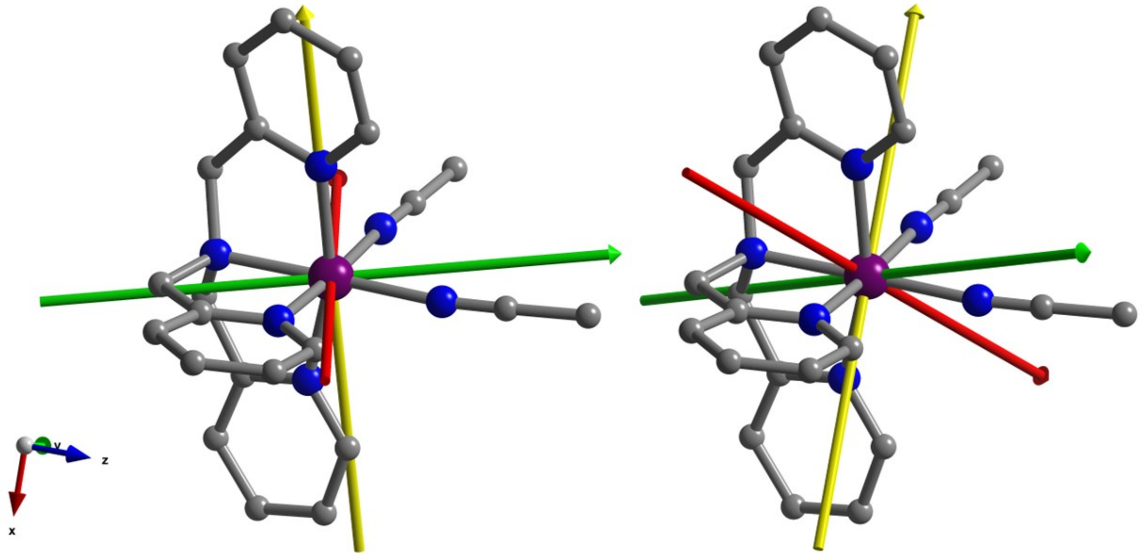

Figure S28. Representation of the possible experimentally determined magnetic reference frame of **1** (left: solution 1, right: solution 2): g_x (intermediate axis) in yellow, g_y (easiest axis) in green, and g_z (hard axis) in red. Solution 1 (on the left) is postulated as the correct solution based on comparison with CASSCF/NEVPT2 results. An xyz crystallographic reference frame is provided for the two solutions (red is x, green is y, blue is z). The two reported solutions both reproduce the data. Hydrogen atoms have been omitted for clarity. Color code: Ni (purple), C (grey), N (blue).

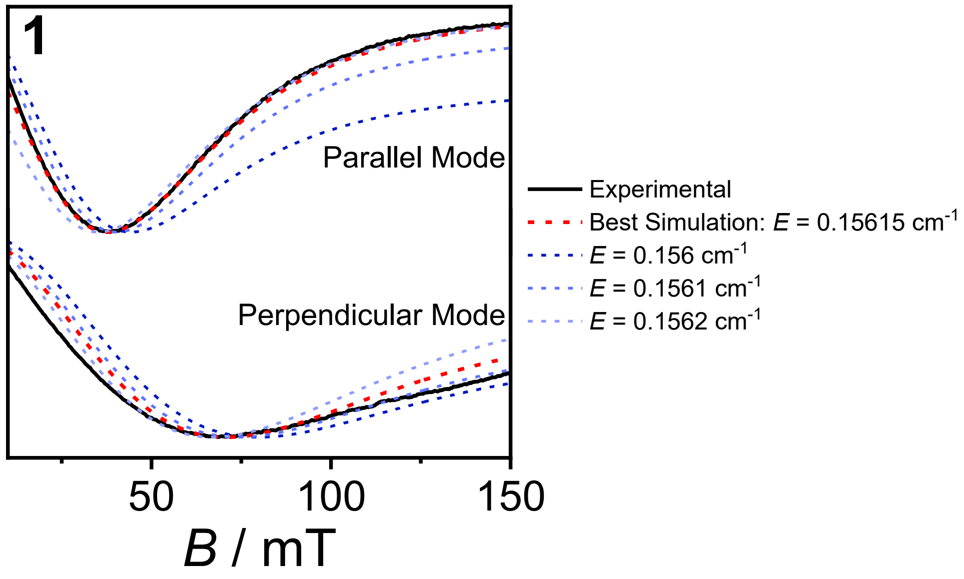

Figure S29. X-band EPR spectra of **1** (black line) in perpendicular (9.387403 GHz) and parallel (9.382712 GHz) mode at 10 K. Red dotted lines is best simulation using parameters g_x = 2.1481, g_y = 2.1628, g_z = 2.1175, D = 1.80 cm^{-1}, E = 0.15615 cm^{-1}. To test for the precision of E, alternate simulations are presented in the dotted blue lines (maintaining g_x = 2.1481, g_y = 2.1628, g_z = 2.1175, D = 1.80 cm^{-1}) using E = 0.156 (dark blue), 0.1561 (blue), and 0.1562 (light blue) cm^{-1}. Line broadening of [3 60] and [20 39] were used for perpendicular and parallel mode, respectively, across all simulations. E strain of 0.001 cm^{-1} was used for all simulations.

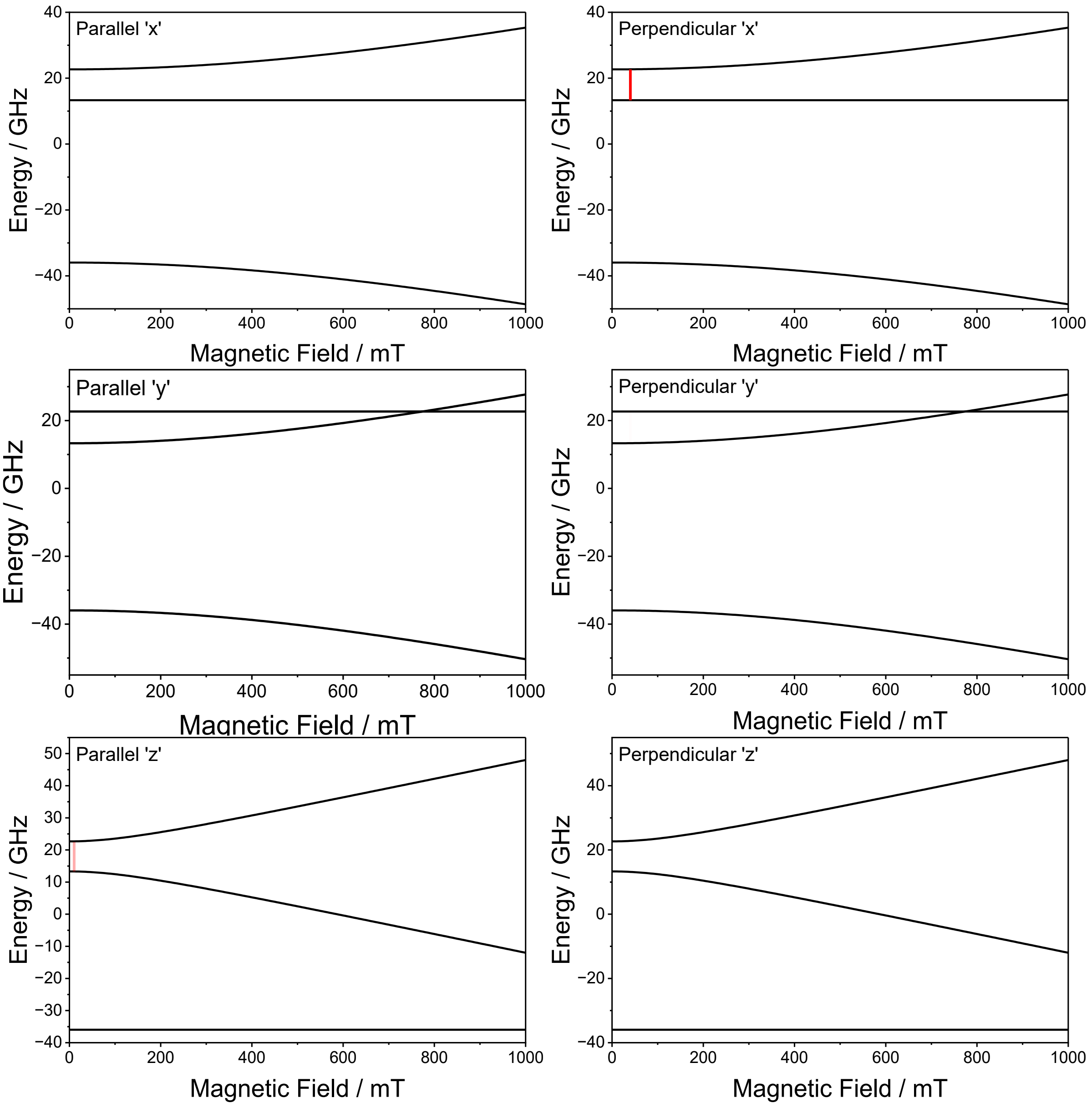

Figure S30. Zeeman plots for **1** for the magnetic field applied along the *x* (top), *y* (middle), and *z* (bottom) directions of the *D* and *g* tensors, with the transitions calculated for the parallel (left) and perpendicular mode (right) cw X-band EPR in red. The parameters used for the simulation: $g_x = 2.1481$, $g_y = 2.1628$, $g_z = 2.1175$, $D = 1.8$ cm^{-1}, and $E = 0.15615$ cm^{-1}.

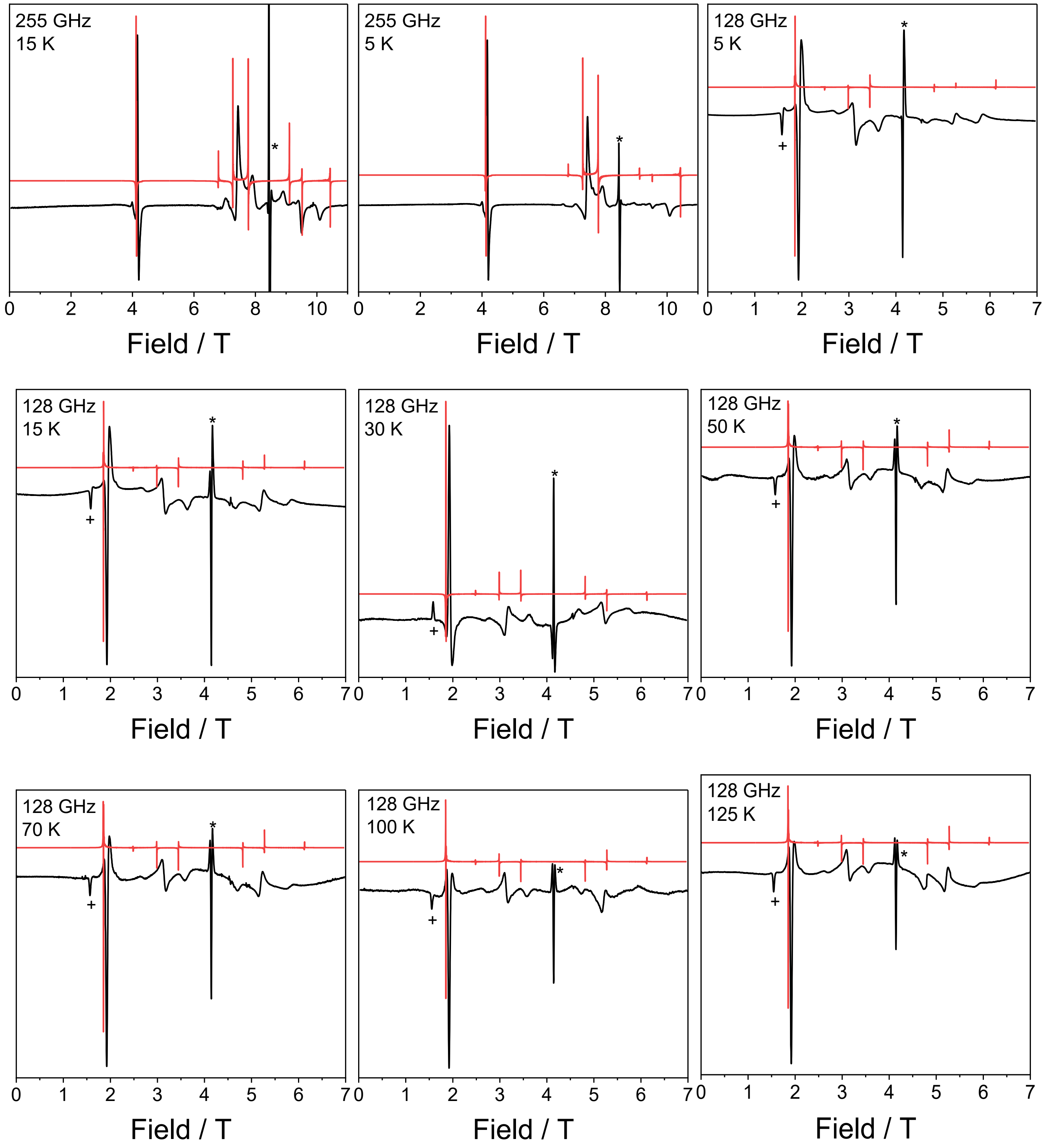

Figure S31. High-frequency EPR spectra of **1** at 255.36 GHz (5 and 15 K) and 127.68 GHz (5, 15, 30, 50, 70, 100 and 150 K) in black (* denotes double quantum transition, + denotes impurity). Simulation in red from the Spin Hamiltonian parameters derived from CTM: $g_x = 2.1481$, $g_y = 2.1628$, $g_z = 2.1175$, $D = 1.80$ cm^{-1}, $E = 0.152$ cm^{-1}.

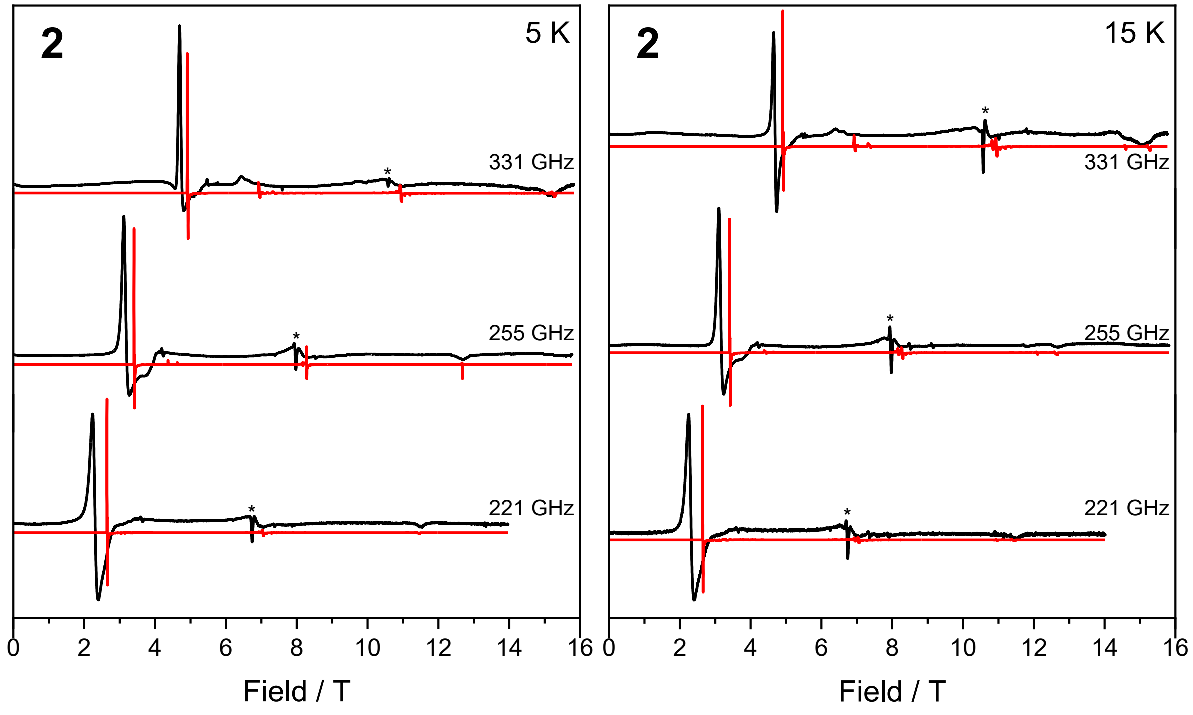

Figure S32. High-frequency EPR spectra of **2** at 220.8 GHz (5, 15 K), 255.36 GHz (5, 15 K), and 331.2 GHz (5, 15 K) in black (* denotes double quantum transition). Simulation in red from the Spin Hamiltonian parameters derived from CTM: $g_x = 2.076$, $g_y = 2.130$, $g_z = 2.182$, $D = -3.895$ cm^{-1}, $E = 1.264$ cm^{-1}.

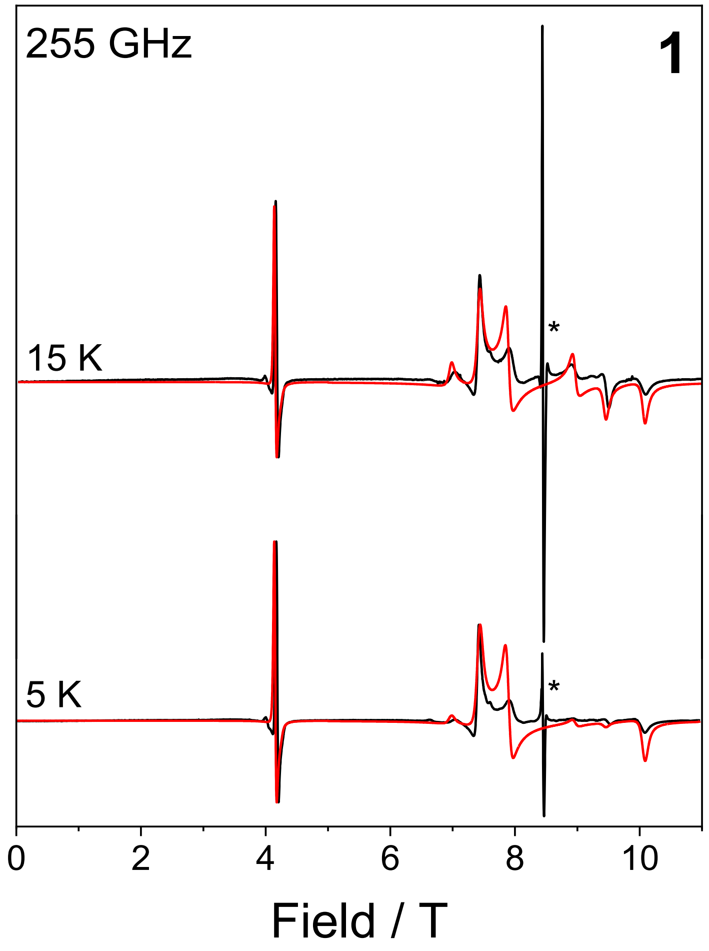

Figure S33. High-frequency EPR spectra of **1** at 255.36 GHz at 5 and 15 K in black (* double quantum transition). Best simulation in red: $g_x = 2.155(5)$, $g_y = 2.155(5)$, $g_z = 2.14(1)$, $D = 1.55(5)$ cm^{-1}, $E = 0.16(1)$ cm^{-1}.

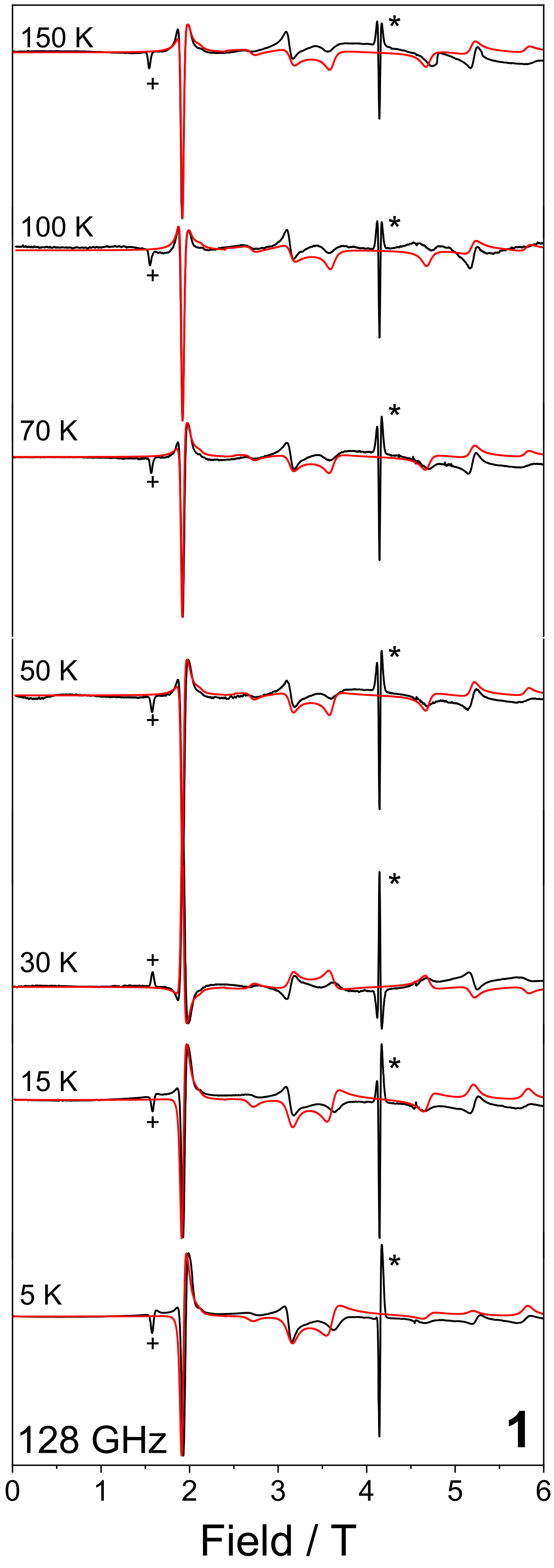

Figure S34. High-frequency EPR spectra of **1** at 127.68 GHz at 5, 15, 30, 50, 70, 100 and 150 K in black (* denotes double quantum transition, + denotes the ΔM = ±2 transition of the air-damaged fraction of the sample). Best simulation in red: $g_x = 2.155(5)$, $g_y = 2.155(5)$, $g_z = 2.14(1)$, $D = 1.55(5)$ cm^{-1}, $E = 0.16(1)$ cm^{-1}.

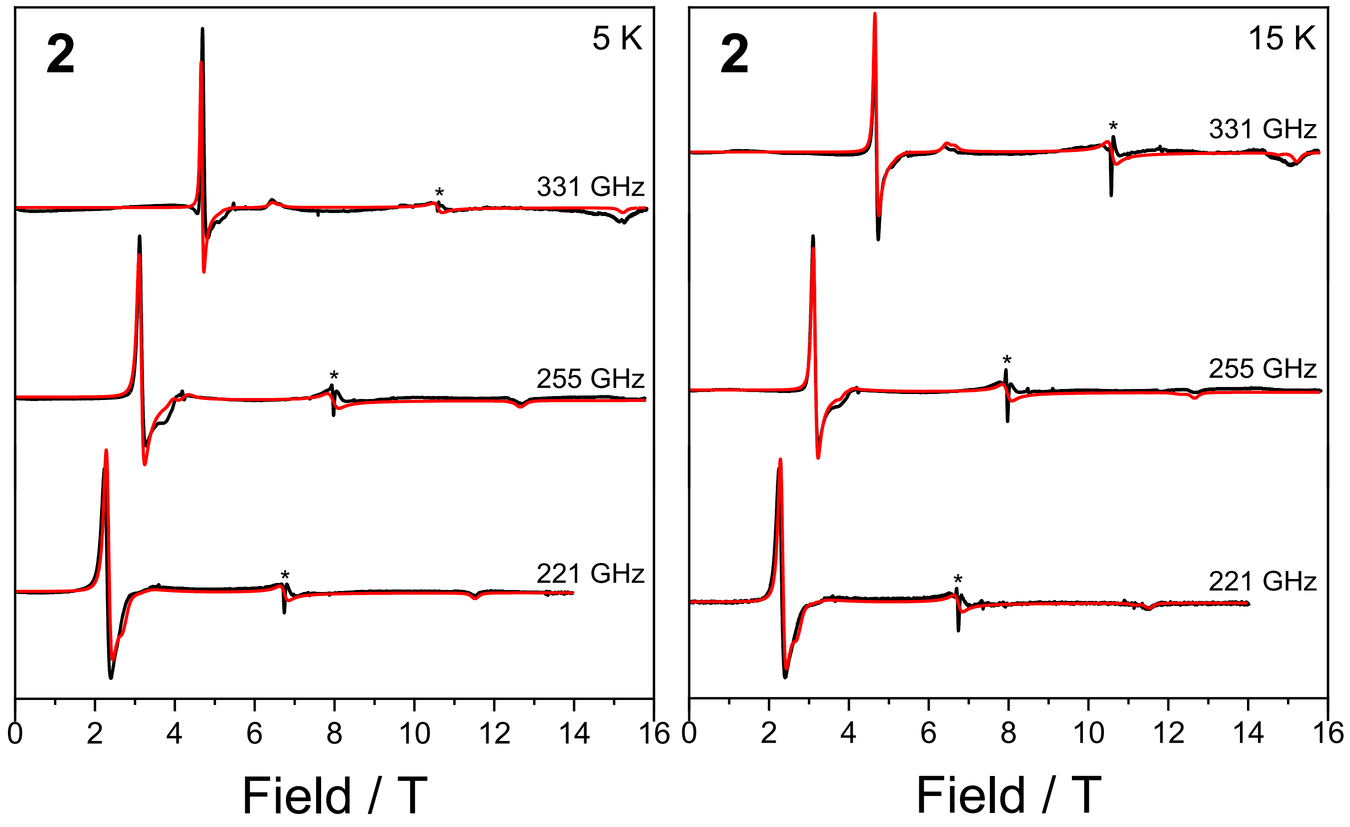

Figure S35. High-frequency EPR spectra of **2** at 220.8, 255.36, and 331.2 GHz at 5 and 15 K in black (* denotes double quantum transition). Best simulation in red: g_x = 2.14(1), g_y = 2.16(1), g_z = 2.21(1), D = −4.25(5) cm^{-1}, E = 1.416(16) cm^{-1}

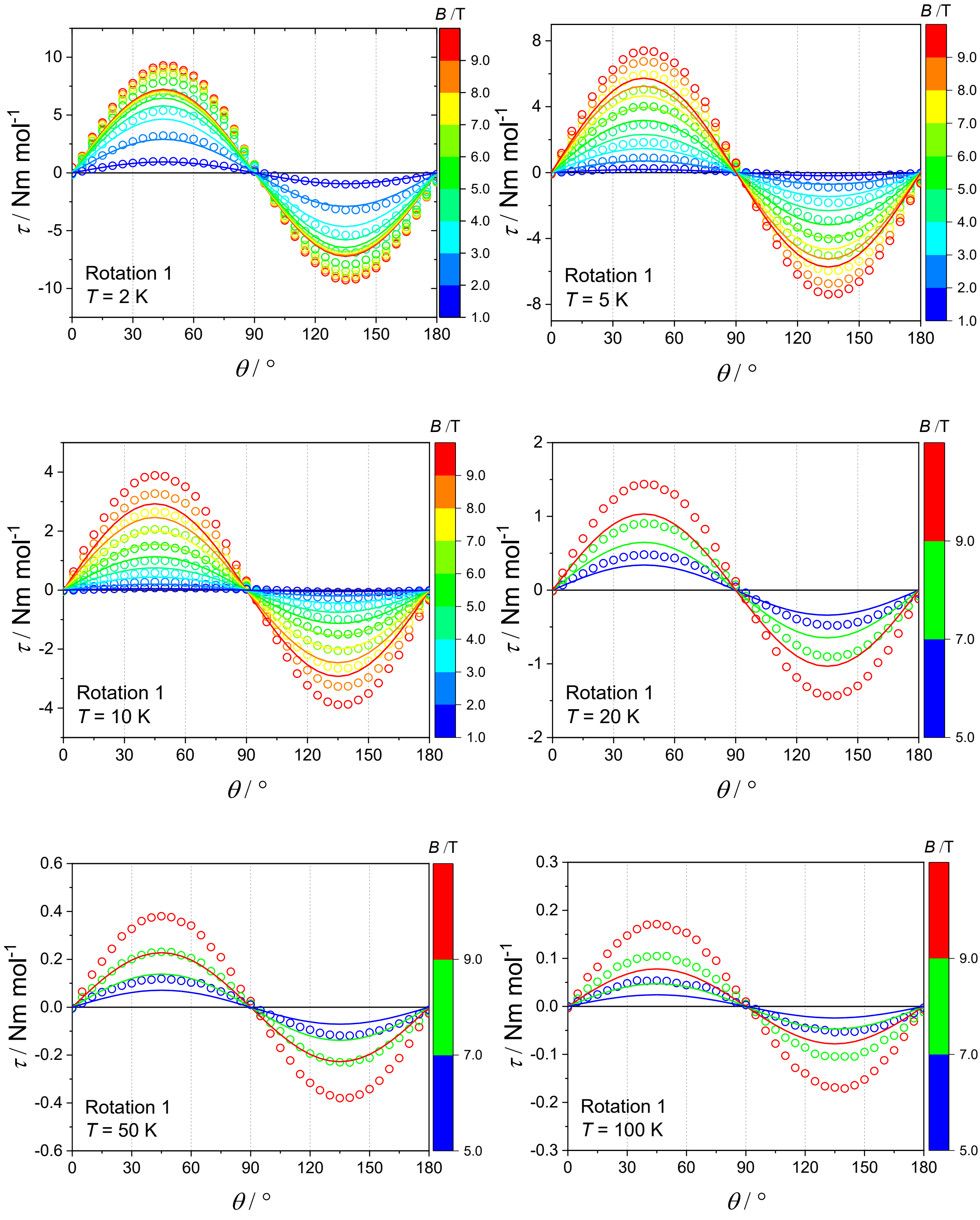

Figure S36. Experimental (circles) and simulated (line) torque curves using the Spin Hamiltonian parameters determined from HF-EPR (g_x = 2.155, g_y = 2.155, g_z = 2.14, D = 1.55 cm^{-1}, E = 0.16 cm^{-1}) for rotation 1 of **1** at 2, 5, 10, 20, 50 and 100 K at various fields.

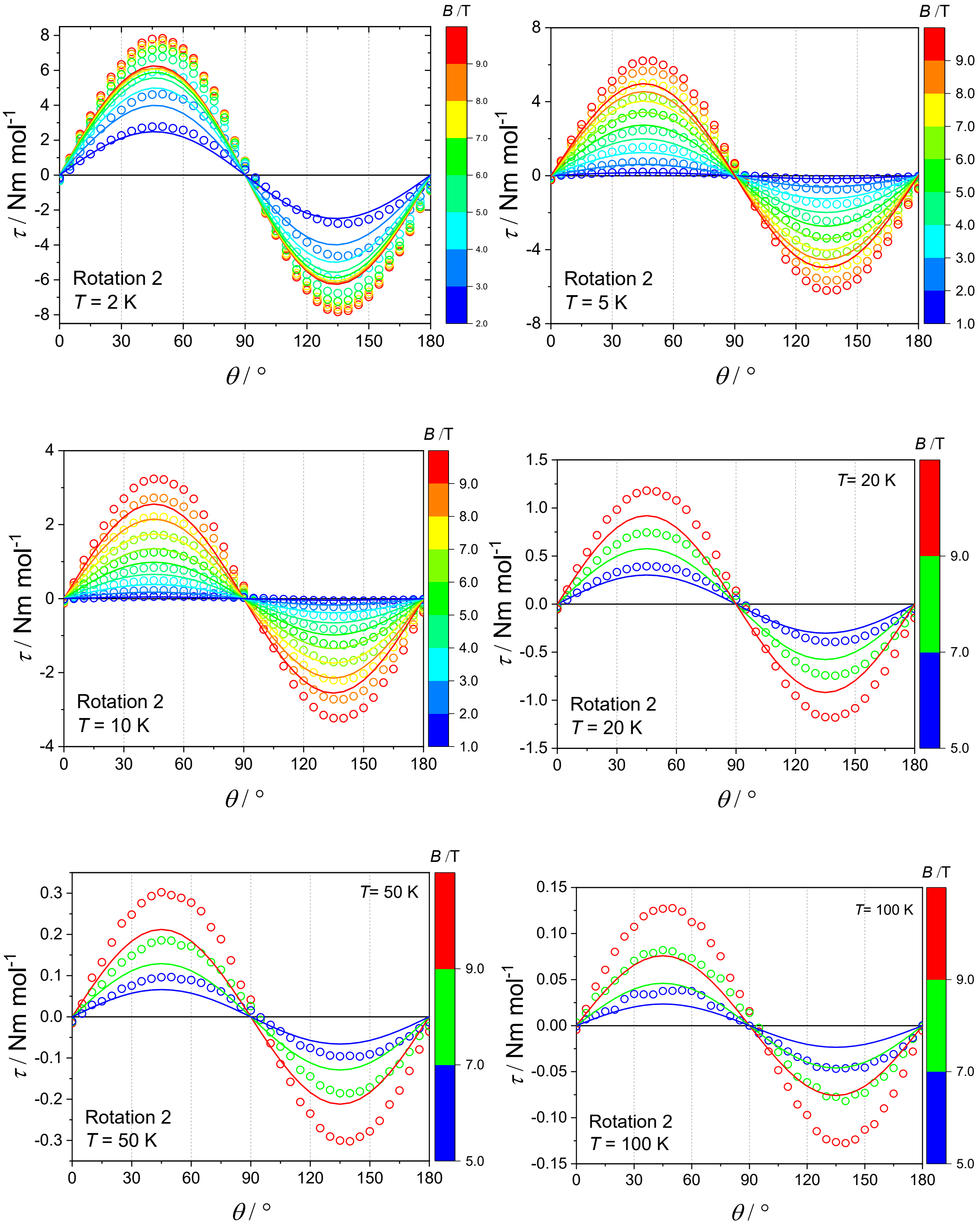

Figure S37. Experimental (circles) and simulated (line) torque curves using the Spin Hamiltonian parameters determined from HF-EPR (g_x = 2.155, g_y = 2.155, g_z = 2.14, D = 1.55 cm^{-1}, E = 0.16 cm^{-1}) for rotation 2 of **1** at 2, 5, 10, 20, 50 and 100 K at various fields.

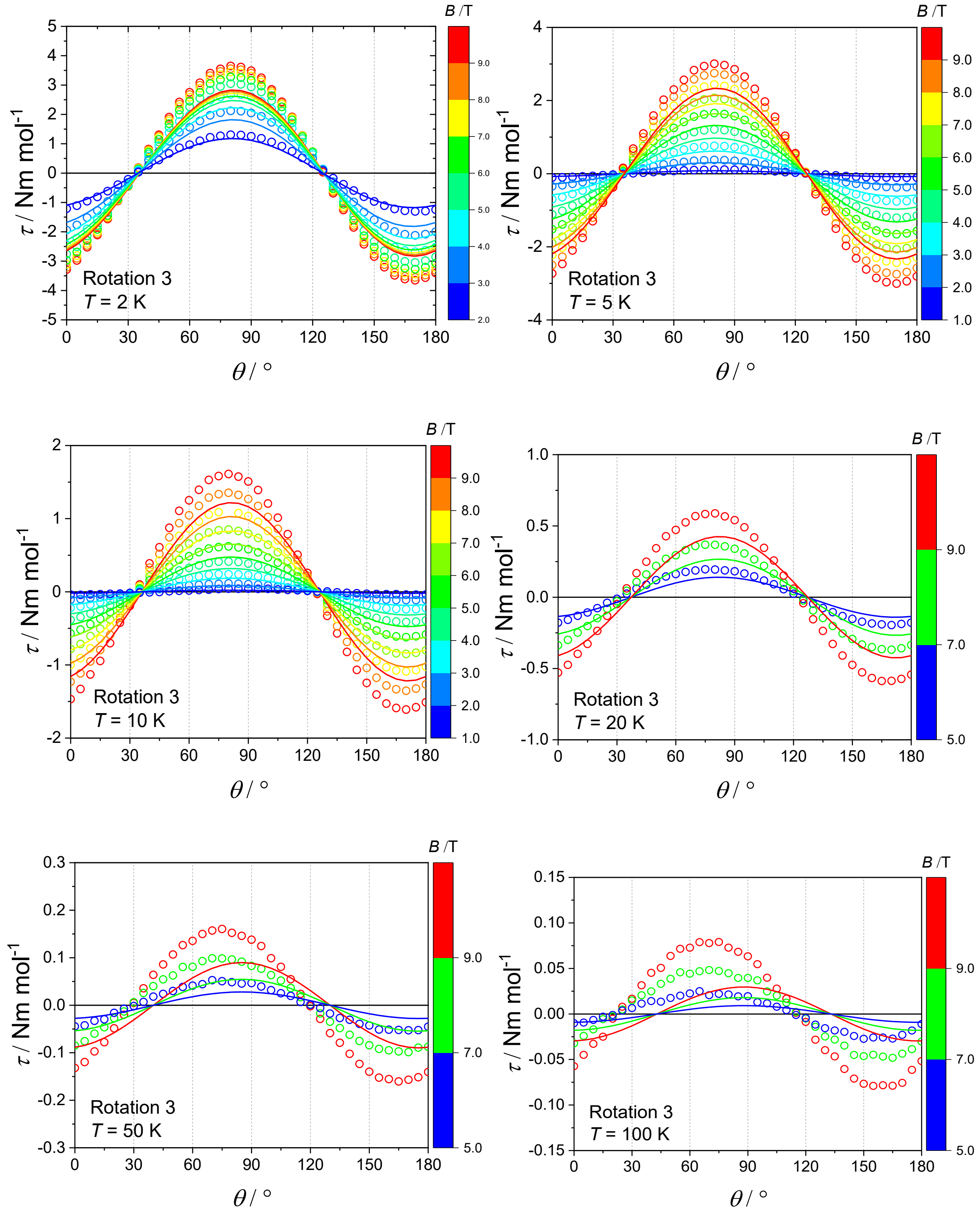

Figure S38. Experimental (circles) and simulated (line) torque curves using the Spin Hamiltonian parameters determined from HF-EPR (g_x = 2.155, g_y = 2.155, g_z = 2.14, D = 1.55 cm^{-1}, E = 0.16 cm^{-1}) for rotation 3 of **1** at 2, 5, 10, 20, 50 and 100 K at various fields.

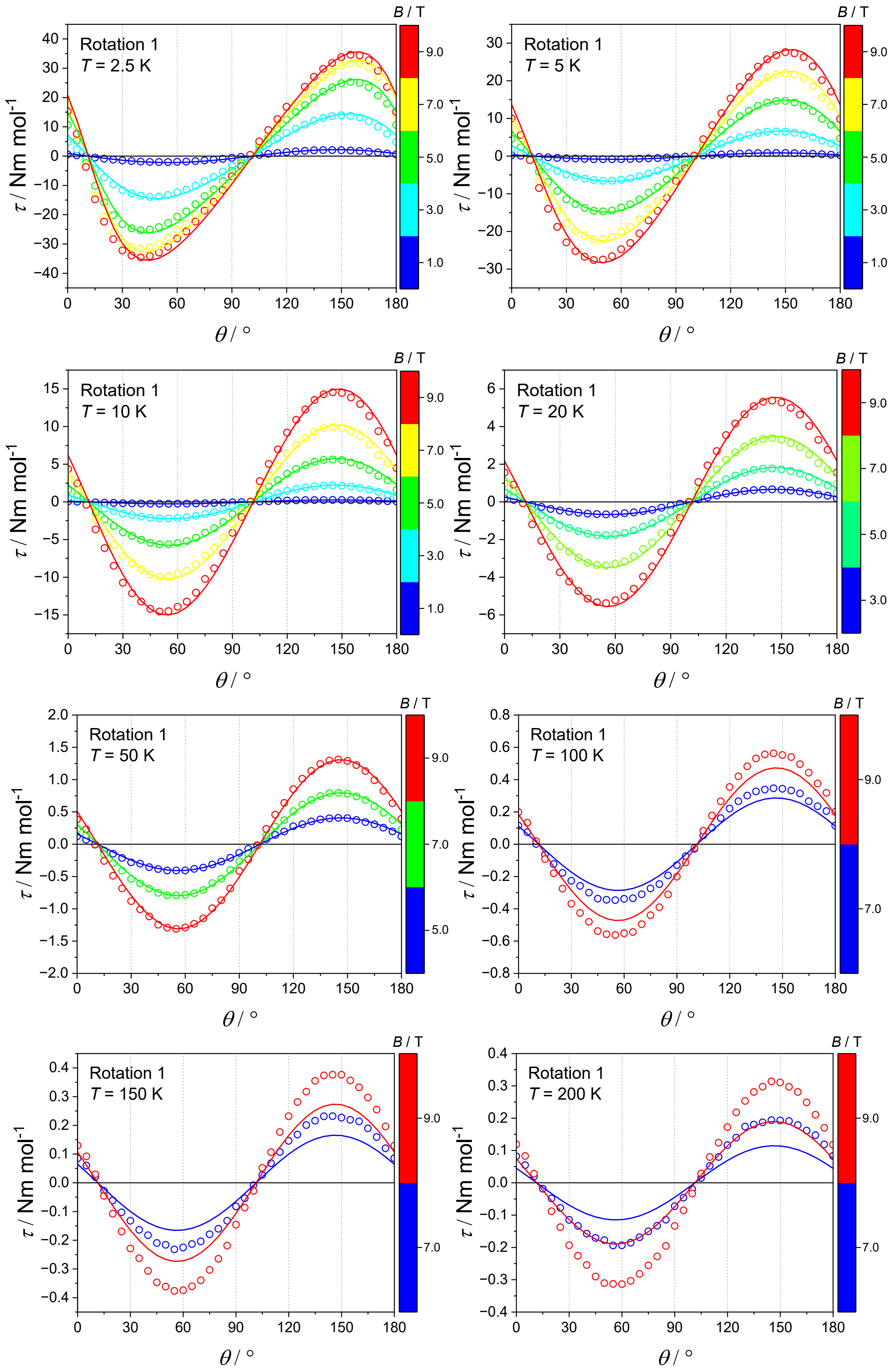

Figure S39. Experimental (circles) and simulated (line) torque curves using the Spin Hamiltonian parameters determined from HF-EPR ($g_x = 2.14$, $g_y = 2.16$, $g_z = 2.21$, $D = -4.25$ cm^{-1}, $E = 1.416$ cm^{-1}) for rotation 1 of **2** at 2, 5, 10, 20, 50 and 100 K at various fields.

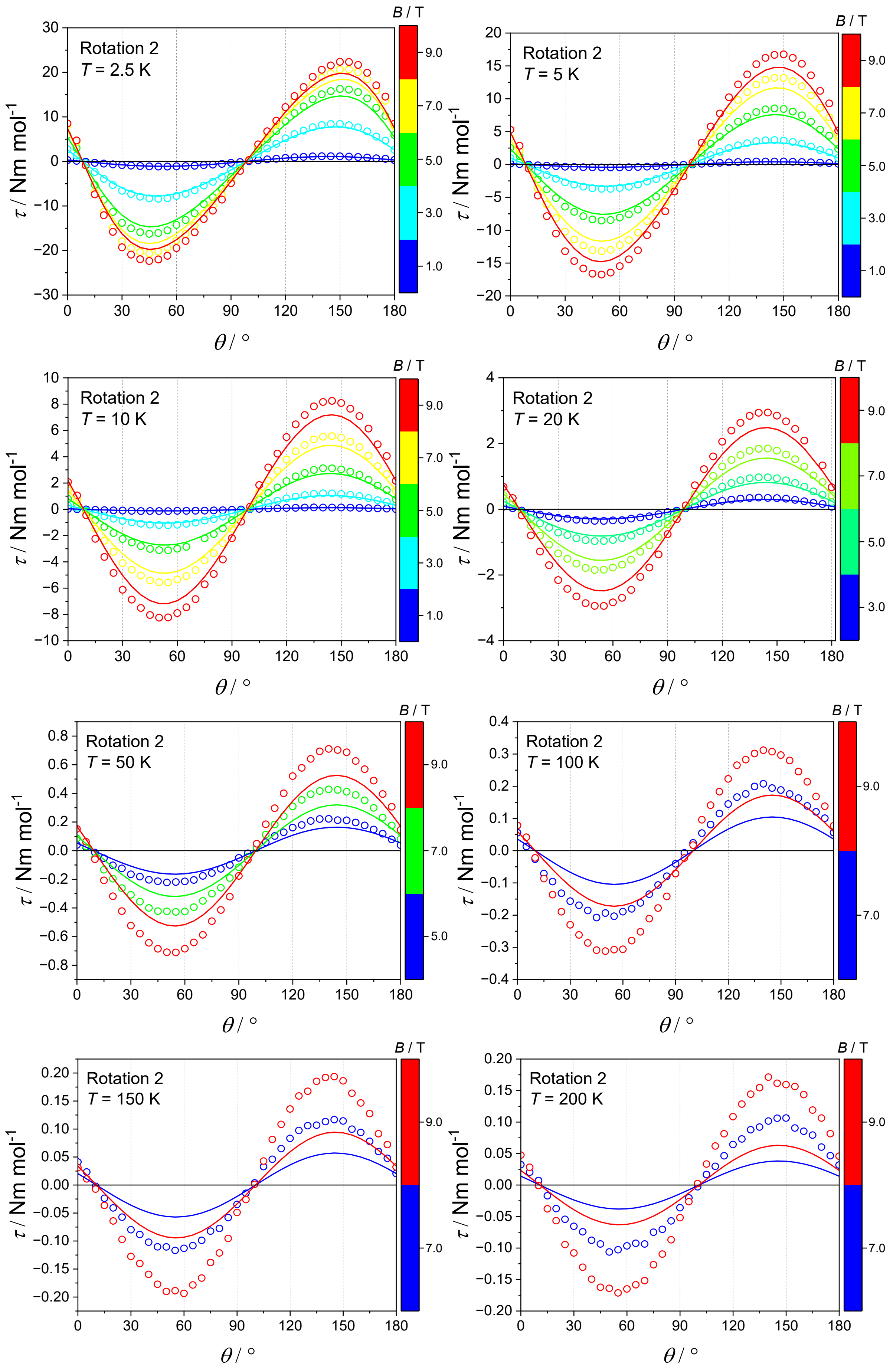

Figure S40. Experimental (circles) and simulated (line) torque curves using the Spin Hamiltonian parameters determined from HF-EPR ($g_x = 2.14$, $g_y = 2.16$, $g_z = 2.21$, $D = -4.25$ cm^{-1}, $E = 1.416$ cm^{-1}) for rotation 2 of **2** at 2, 5, 10, 20, 50 and 100 K at various fields.

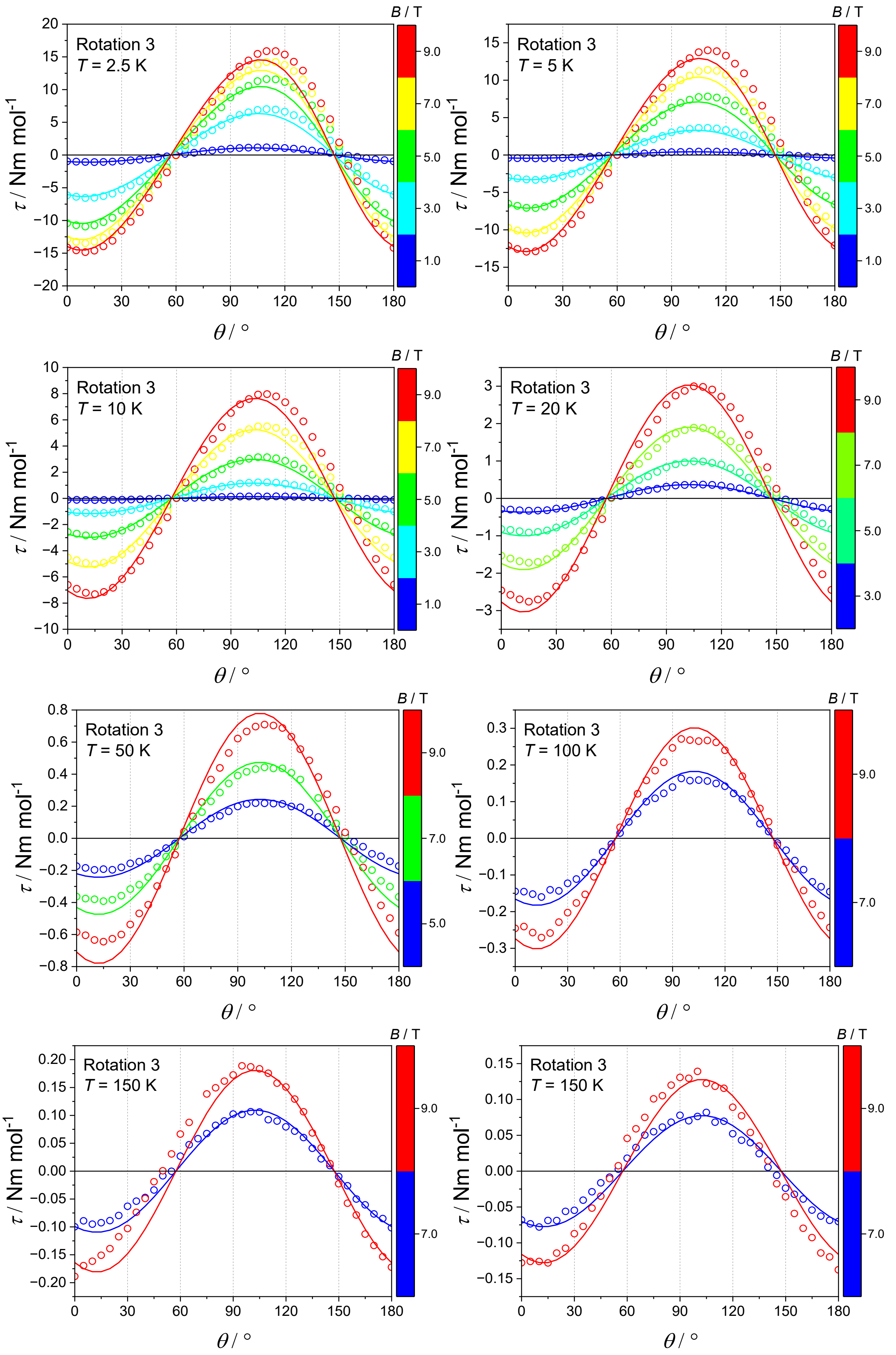

Figure S41. Experimental (circles) and simulated (line) torque curves using the Spin Hamiltonian parameters determined from HF-EPR ($g_x = 2.14$, $g_y = 2.16$, $g_z = 2.21$, $D = -4.25$ cm^{-1}, $E = 1.416$ cm^{-1}) for rotation 3 of **2** at 2, 5, 10, 20, 50 and 100 K at various fields.

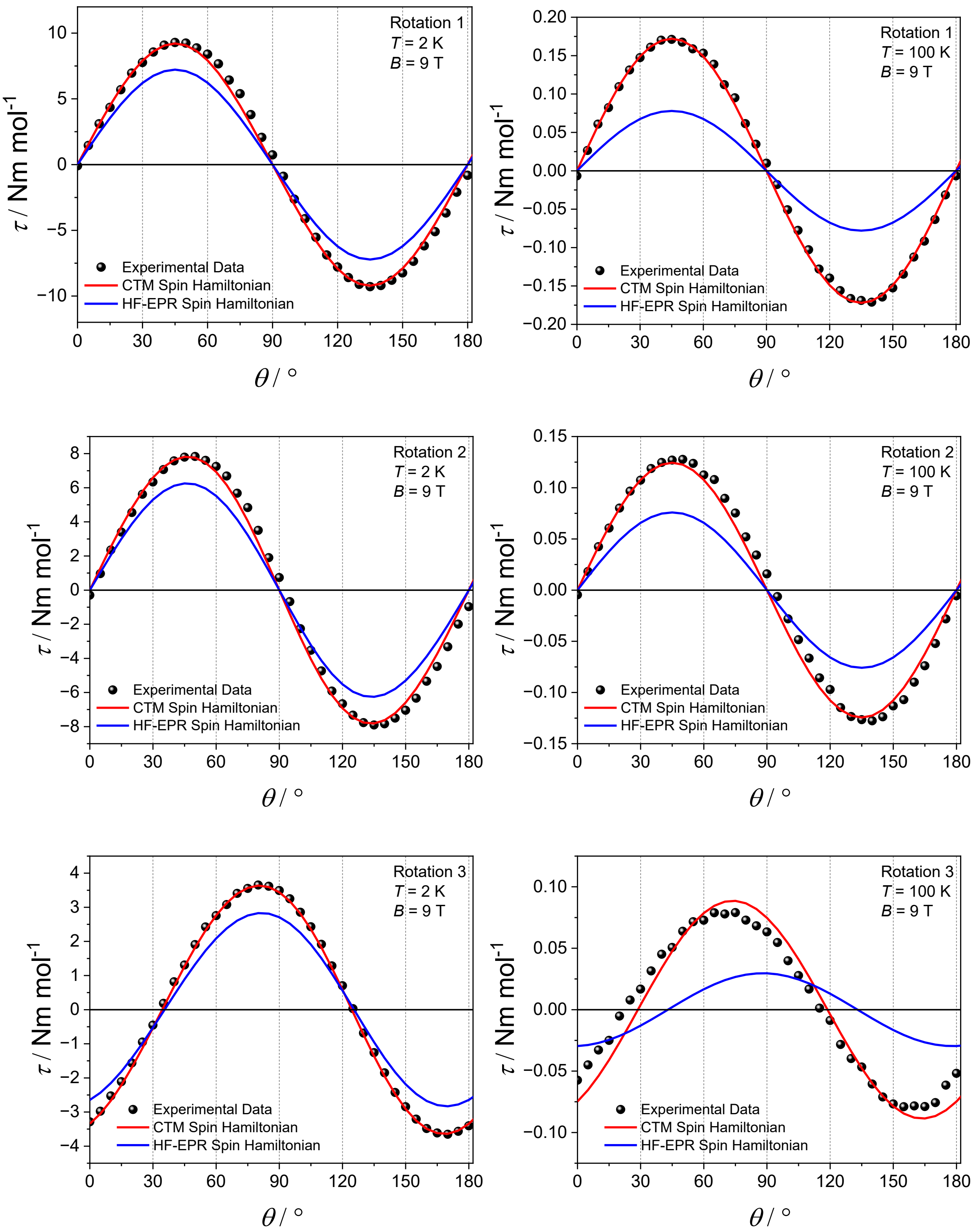

Figure S42. Experimental (circles) and simulated (line) torque curves using the Spin Hamiltonian parameters determined from CTM (red line) (g_x =2.1481, g_y = 2.1628, g_z = 2.1175, D = 1.80 cm^{-1}, E = 0.152 cm^{-1}) and HF-EPR (blue line) (g_x = 2.155, g_y = 2.155, g_z = 2.14, D = 1.55 cm^{-1}, E = 0.16 cm^{-1}) for rotation 1,2, and 3 of **1** at 2 and 100 K at 9 T.

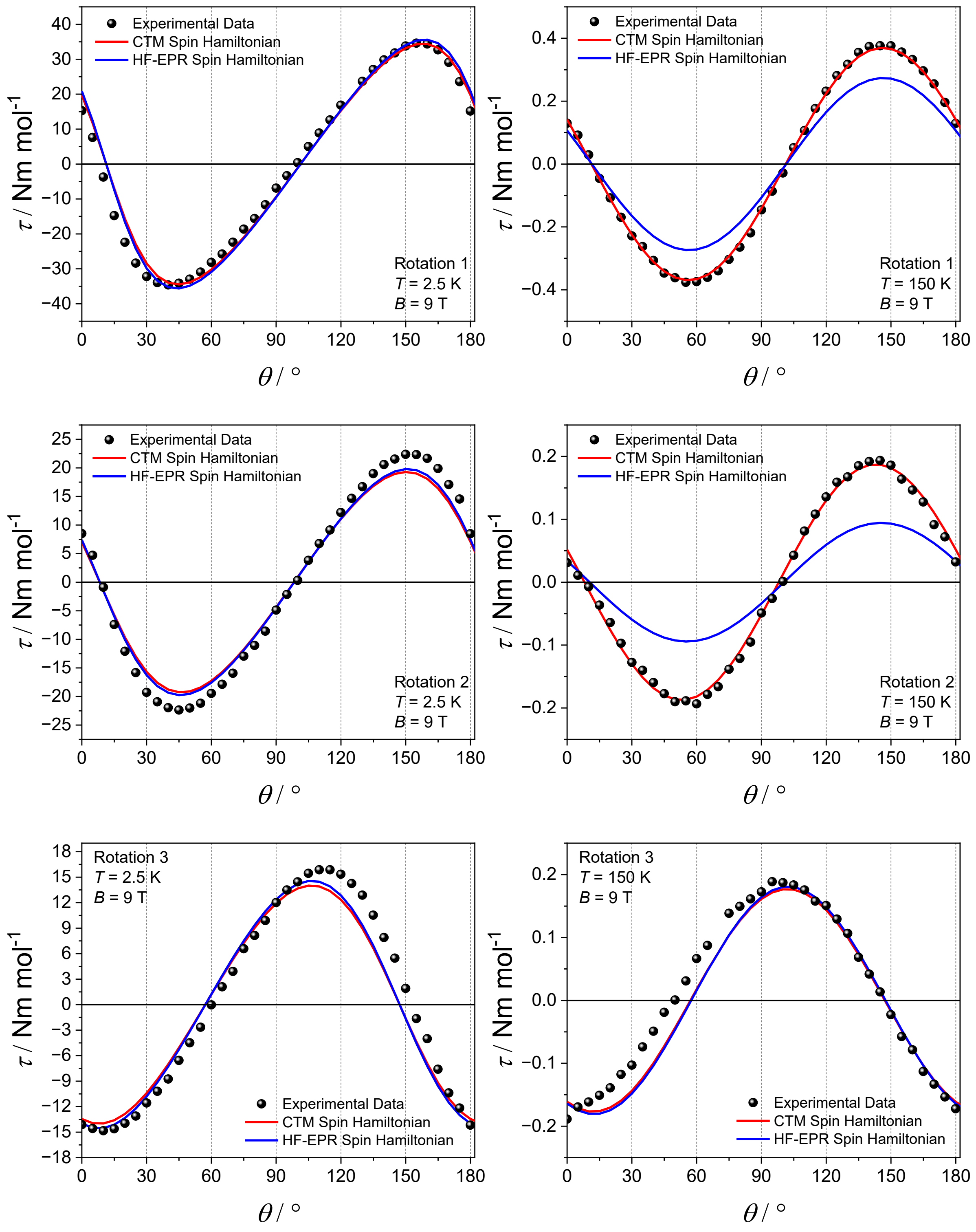

Figure S43. Experimental (circles) and simulated (line) torque curves using the Spin Hamiltonian parameters determined from CTM (red line) (g_x = 2.076, g_y = 2.130, g_z = 2.182, D = −3.895 cm^{-1}, E = 1.264 cm^{-1}) and HF-EPR (blue line) (g_x = 2.14, g_y = 2.16, g_z = 2.21, D = −4.25 cm^{-1}, E = 1.416 cm^{-1}) for rotation 1,2, and 3 of **2** at 2.5 and 150 K at 9 T.

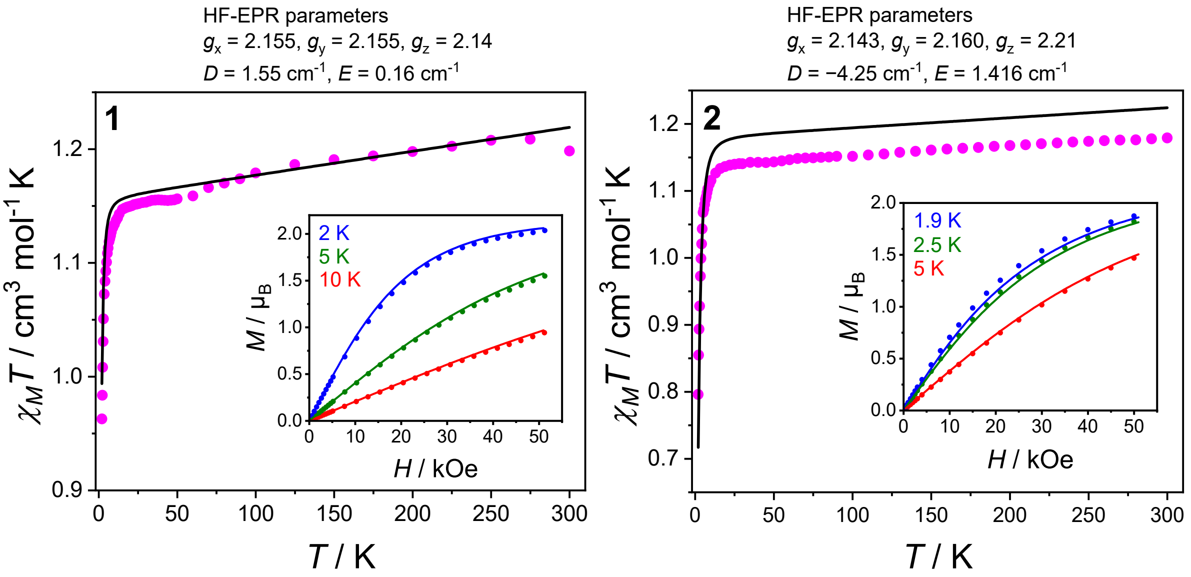

Figure S44. $\chi_M T$ vs T for **1** and **2**. Inset: magnetization vs field at three temperatures. Solid lines are simulations using HF-EPR data best-fit parameters (Table 1) and inclusion of temperature-independent paramagnetism (TIP): **1**: $g_x = 2.155$, $g_y = 2.155$, $g_z = 2.14$, $D = 1.55$ cm^{-1}, $E = 0.16$ cm^{-1}, TIP = 2.1×10^{-4} cm^3 mol^{-1}. **2**: $g_x = 2.14$, $g_y = 2.16$, $g_z = 2.21$, $D = -4.25$ cm^{-1}, $E = 1.416$ cm^{-1}, TIP = 1.5×10^{-4} cm^3 mol^{-1}.

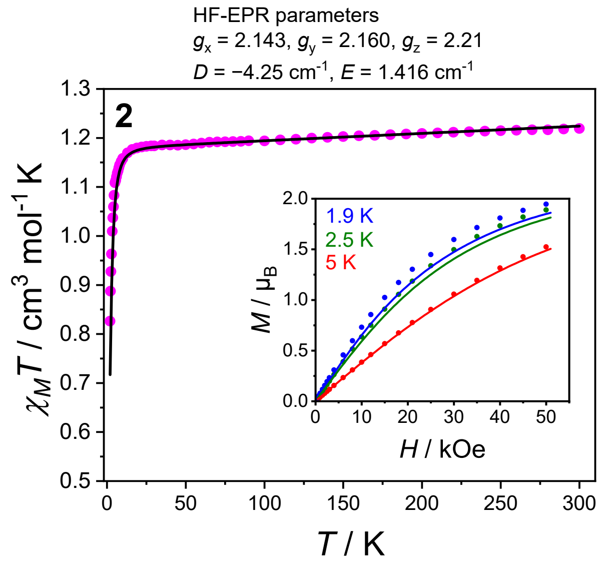

Figure S45. $\chi_M T$ vs T for **2**. Inset: magnetization vs field at three temperatures. Solid lines are simulations using HF-EPR data best-fit parameters (Table 1) and inclusion of temperature-independent paramagnetism (TIP): **1**: $g_x = 2.155$, $g_y = 2.155$, $g_z = 2.14$, $D = 1.55$ cm^{-1}, $E = 0.16$ cm^{-1}, TIP = 2.1×10^{-4} cm^3 mol^{-1}. The experimental data has been adjusted to account for errors in the mass of the sample, such that the room temperature experimental $\chi_M T$ matches the simulated data. This is to show that an error in powder mass or molar mass cannot account for the discrepancies between the experimental data and the simulation using the Spin Hamiltonian parameters derived from HF-EPR.

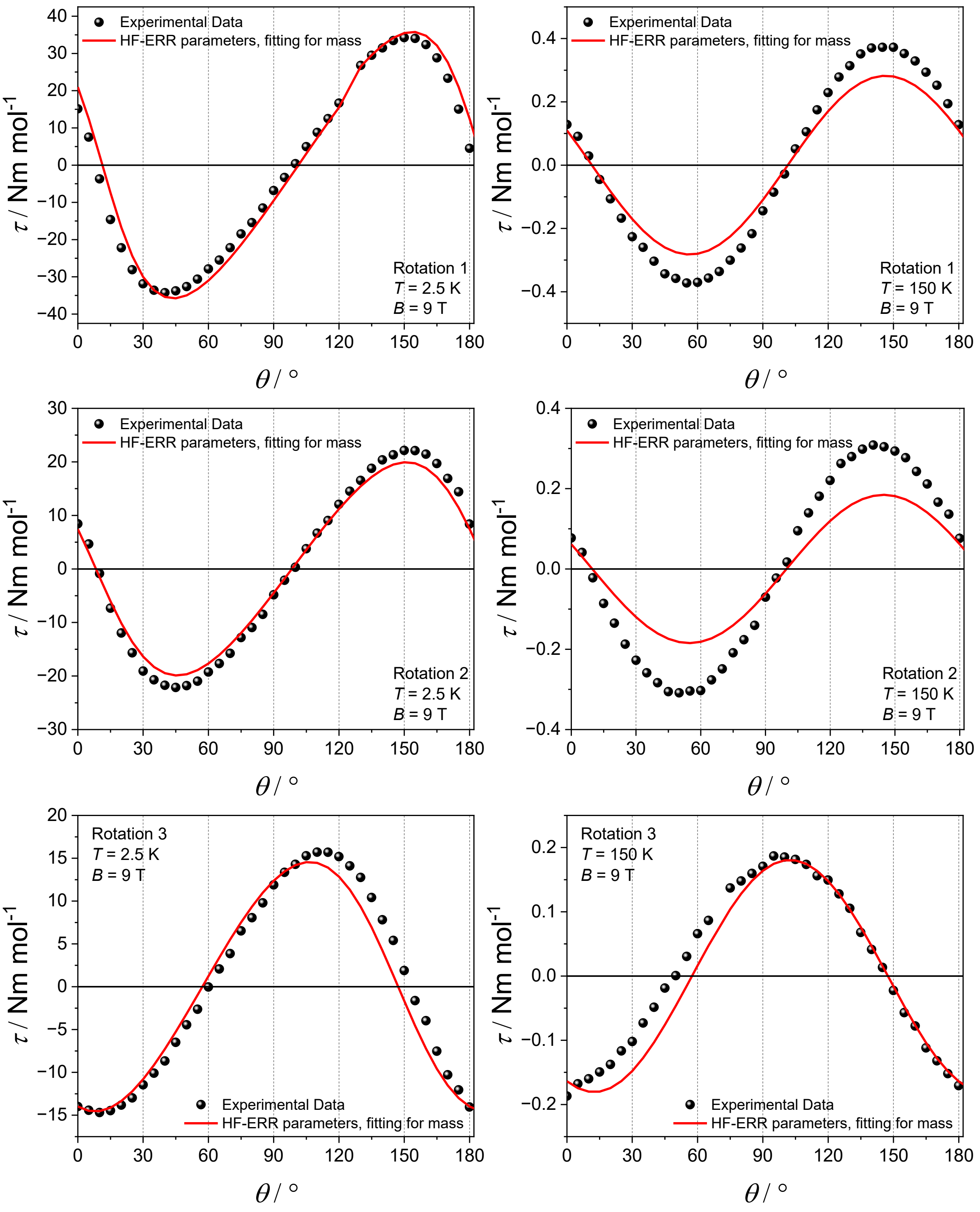

Figure S46. Experimental (circles) and simulated (line) torque curve determined by fitting the crystal mass and fixing the Spin Hamiltonian parameters determined from HF-EPR ($g_x = 2.14$, $g_y = 2.16$, $g_z = 2.21$, $D = -4.25$ cm^{-1}, $E = 1.416$ cm^{-1}) and the Euler angles for rotation 1,2, and 3 of **2** at 2.5 and 150 K at 9 T.

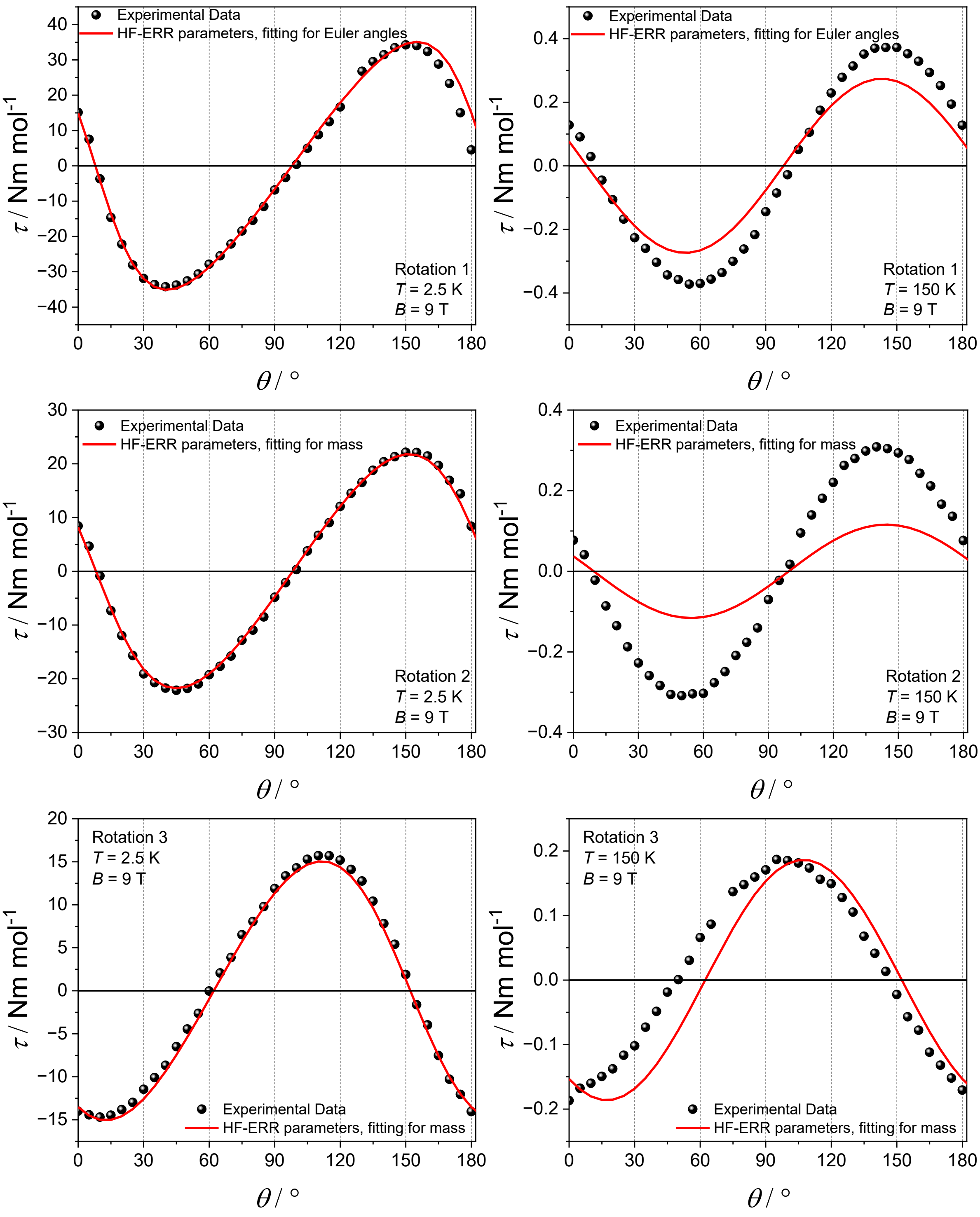

Figure S47. Experimental (circles) and simulated (line) torque curve determined by fitting the Euler angles and fixing the Spin Hamiltonian parameters determined from HF-EPR ($g_x = 2.14$, $g_y = 2.16$, $g_z = 2.21$, $D = -4.25$ cm^{-1}, $E = 1.416$ cm^{-1}) and the crystal mass for rotation 1,2, and 3 of **2** at 2.5 and 150 K at 9 T.

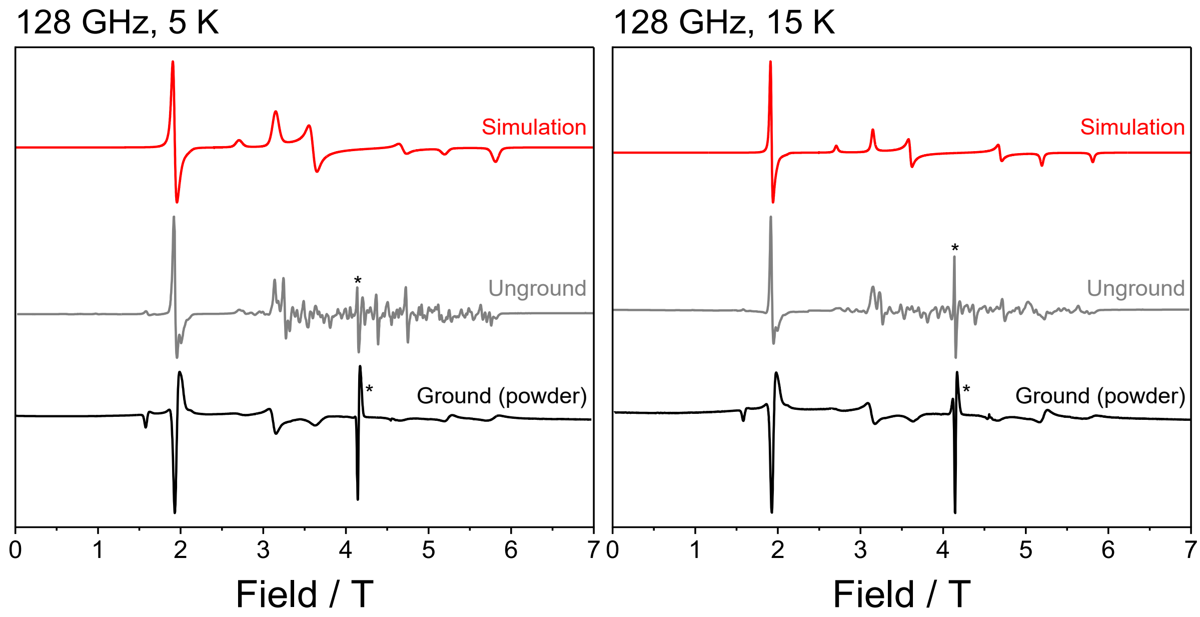

Figure S48. High-frequency EPR spectra of **1** at 127.68 GHz at 5 and 15 K on a ground (powder) sample in black and an unground (non-powder) sample in grey (* denotes double quantum transition). Simulation in red: $g_x = 2.155(5)$, $g_y = 2.155(5)$, $g_z = 2.14(1)$, $D = 1.55(5)$ cm^{-1}, $E = 0.16(1)$ cm^{-1}. In the unground sample there are additional resonances as expected for a non-statistical distribution of microcrystals.

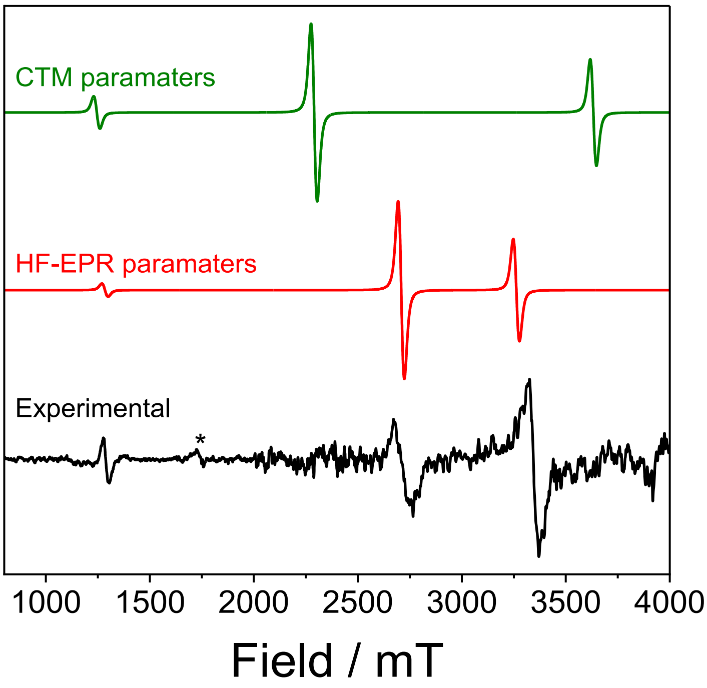

Figure S49. Preliminary continuous wave W-band (94.01 GHz) EPR spectra of a single crystal of **2** at 35 K, with the magnetic field parallel to −*c**. Red line is simulation using parameters from HF-EPR (g_x = 2.155, g_y = 2.155, g_z = 2.14, D = 1.55 cm^{-1}, E = 0.16 cm^{-1}). Green line is simulation using parameters from CTM (g_x =2.1481, g_y = 2.1628, g_z = 2.1175, D = 1.80 cm^{-1}, E = 0.152 cm^{-1}). Line broadening of [25 25] mT was used. The spectrum is better simulated with the HF-EPR parameters compared with those determined from CTM, supporting that the difference between CTM and HF-EPR is not related to a single-crystal vs powder effect. * Signal from an impurity in the cavity.

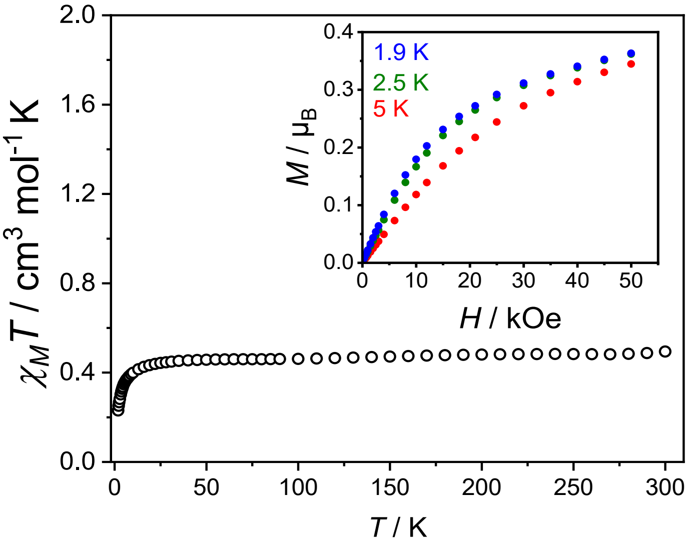

Figure S50. $\chi_M T$ vs T for $[Fe^{II}(tpa)(MeCN)_2](OTf)_2$. Inset: magnetization vs field at three temperatures. In theory complex $[Fe(tpa)(MeCN)_2](OTf)_2$ is low spin Fe^{II} and should be diamagnetic.[1,32] However, this complex was shown to undergo a low spin/high spin Fe^{II} spin crossover interconversion in solution.[1,32] This results in trapped high spin Fe^{II} population in the solid-state, accounting for the $\chi_M T$ ~0.45 –0.5 cm^3 mol^{-1} K between 25 and 300 K.

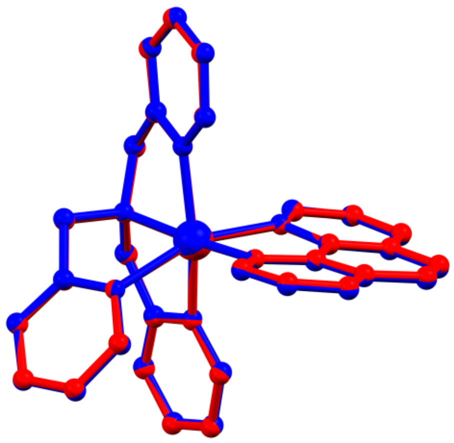

Figure S51. Cationic structure of **2** determined by X-ray diffraction at 100 K (blue) and 293 K (red). Octahedral SHAPE: 2.78 (100 K), 2.76 (293 K). Σ: 117° (100 K), 117° (293 K). Θ: 388° (100 K), 386° (293 K).

Table S9. Spin Hamiltonian parameters for **2** calculated using CASSCF/NEVPT2 on the 293 and 100 K structures (non-optimized), and optimized structure at 0 K.

	g_x	g_y	g_z	D / cm^{-1}	E/D
293 K	2.176	2.198	2.200	−5.79	0.28
100 K	2.173	2.196	2.225	−5.67	0.27
0 K optimized	2.192	2.217	2.244	−5.95	0.33

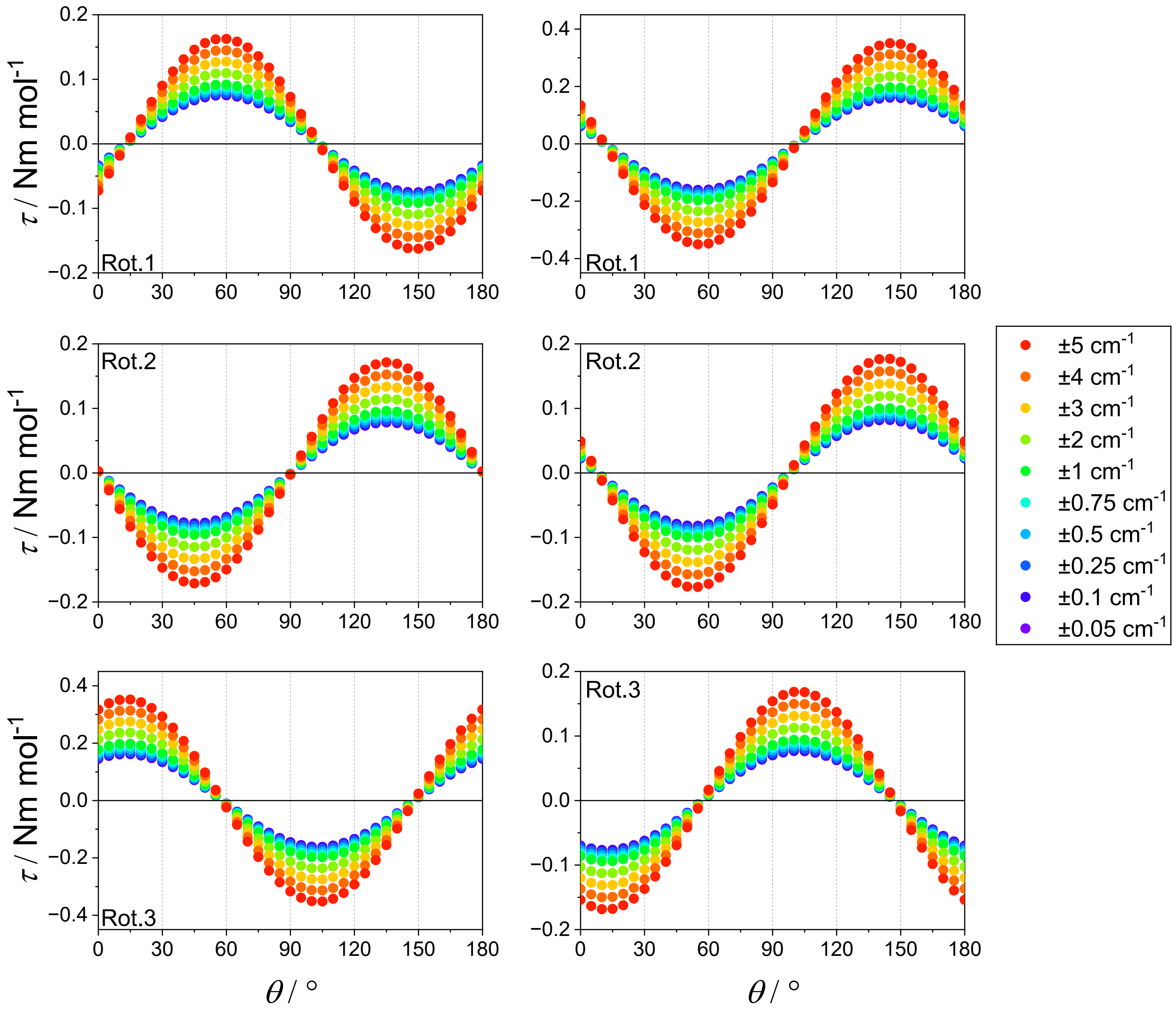

Figure S52. Simulated experimental CTM curves for a $g_x = 2.00$, $g_y = 2.02$, $g_z = 2.04$ system (using euler angles, sample mass, molar mass, and scaling factor used for the CTM fits of **2**) with varying D values at 100 K and 9 T.

Table S10. The g_x, g_y and g_z values determined from fitting simulated 'experimental' torque curves for $g_x = 2.00$, $g_y = 2.02$, $g = 2.04$ (for for −*D*) and $g_x = 2.02$, $g_y = 2.04$, $g_z = 2.00$ (for +*D*) at 100 K and 9 T using Euler angles, sample mass, molar mass, and scaling factor for **2** for a range of *D* values (assuming *E*/*D* = 1/3).

D cm^{-1}	g_x	g_y	g_z	Mean Absolute Error [a]
0.05	2.01999	2.04024	1.99976	1.63×10^{-4}
0.1	2.01998	2.04048	1.99951	3.30×10^{-4}
0.25	2.01996	2.04121	1.99879	8.20×10^{-4}
0.5	2.01997	2.04241	1.99757	1.62×10^{-3}
0.75	2.01996	2.04363	1.99637	2.43×10^{-3}
1	2.01997	2.04485	1.99518	3.23×10^{-3}
2	2.02009	2.04984	1.99049	6.48×10^{-3}
3	2.02035	2.05496	1.98592	9.80×10^{-3}
4	2.02076	2.06024	1.98145	1.32×10^{-2}
5	2.02127	2.06551	1.97721	1.65×10^{-2}
−0.05	1.9998	2.02	2.0402	1.33×10^{-4}
−0.1	1.9995	2.02	2.0405	3.33×10^{-4}
−0.25	1.9988	2.02	2.0412	8.00×10^{-4}
−0.5	1.9976	2.02	2.0425	1.63×10^{-3}
−0.75	1.9965	2.0201	2.0437	2.43×10^{-3}
−1	1.9953	2.0201	2.0450	3.27×10^{-3}
−2	1.991	2.0206	2.0503	6.63×10^{-3}
−3	1.987	2.0214	2.0560	1.01×10^{-2}
−4	1.9834	2.0226	2.0620	1.37×10^{-2}
−5	1.9801	2.0241	2.0683	1.74×10^{-2}

[a] Mean absolute error from $g_x = 2.00$, $g_y = 2.02$, $g_z = 2.04$ (for −*D*) and $g_x = 2.02$, $g_y = 2.04$, $g_z = 2.00$ (for +*D*). i.e average difference between g_{fixed} and g_{fit}

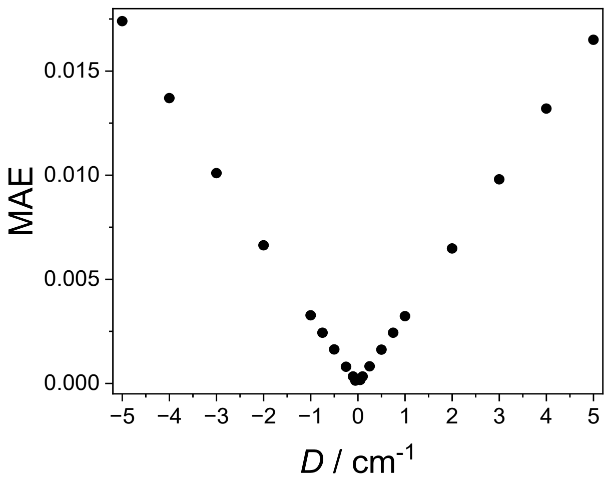

Figure S53. Mean absolute error between g_{set} and g_{fit} for various magnitudes of D.

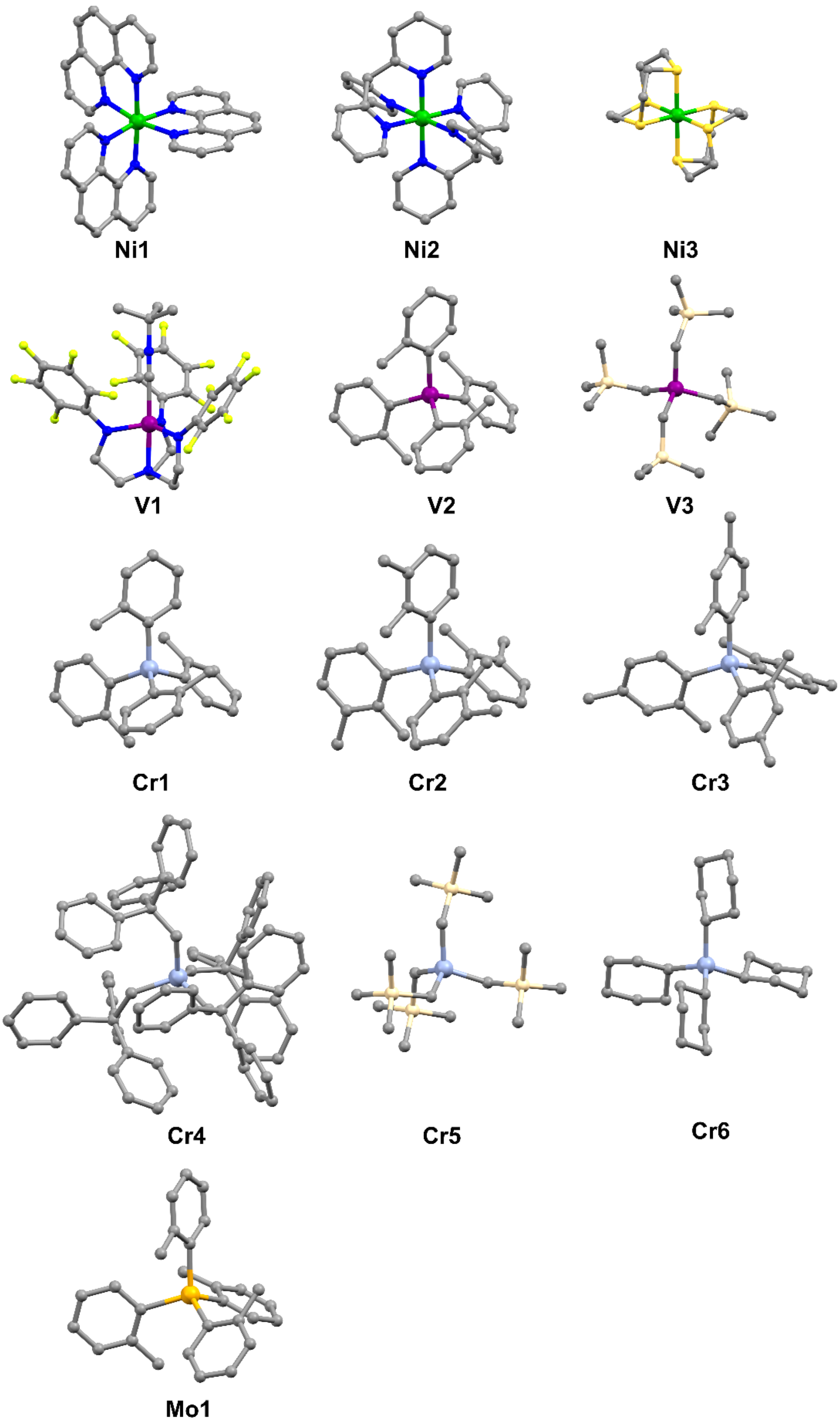

Figure S54. Molecular structures of literature *S* = 1 qubits and optically-addressable qubits. Carbon = grey, N = blue, S = yellow, F = light green, Si = cream, Ni = green, V = purple, Cr = light blue, Mo = orange.[33–38]

REFERENCES

(1) Ward, A. L.; Elbaz, L.; Kerr, J. B.; Arnold, J. Nonprecious Metal Catalysts for Fuel Cell Applications: Electrochemical Dioxygen Activation by a Series of First Row Transition Metal Tris(2-Pyridylmethyl)Amine Complexes. *Inorg. Chem.* **2012**, *51*, 4694–4706.

(2) Gransbury, G. K.; Livesay, B. N.; Janetzki, J. T.; Hay, M. A.; Gable, R. W.; Shores, M. P.; Starikova, A.; Boskovic, C. Understanding the Origin of One- or Two-Step Valence Tautomeric Transitions in Bis(Dioxolene)-Bridged Dinuclear Cobalt Complexes. *J Am Chem Soc* **2020**, *142*, 10692–10704.

(3) Bushmin, D. S.; Samsonenko, D. G.; Talsi, E. P.; Lyakin, O. Y.; Bryliakov, K. P. Diverting Ni-Catalyzed Direct Benzylic C−H Hydroxylation towards Trifluoroethoxylation. *ChemCatChem* **2024**, *16*, e202301346.

(4) Sheldrick, G. M. SHELXT – Integrated Space-Group and Crystal-Structure Determination. *Acta Crystallogr., Sect. A: Found. Crystallogr.* **2015**, *71*, 3–8.

(5) Sheldrick, G. M. Crystal Structure Refinement with SHELXL. *Acta Crystallogr., Sect C: Struct. Chem.* **2015**, *71*, 3–8.

(6) Dolomanov, O. V.; Bourhis, L. J.; Gildea, R. J.; Howard, J. A. K. K.; Puschmann, H. OLEX2: A Complete Structure Solution, Refinement and Analysis Program. *J. Appl. Crystallogr.* **2009**, *42*, 339–341.

(7) Bain, G. A.; Berry, J. F. Diamagnetic Corrections and Pascal's Constants. *J. Chem. Educ.* **2008**, *85*, 532.

(8) Stoll, S.; Schweiger, A. EasySpin, a Comprehensive Software Package for Spectral Simulation and Analysis in EPR. *J. Mag. Res.* **2006**, *178*, 42–55.

(9) Barra, A. L.; Hassan, A. K.; Janoschka, A.; Schmidt, C. L.; Scha, V. Broad-band quasi-optical HF-EPR spectroscopy: Application to the study of the ferrous iron center from a rubredoxin mutant. *Appl. Magn. Reson.* **2006**, *30*, 385–397.

(10) Suaud, N.; Rogez, G.; Rebilly, J. N.; Bouammali, M. A.; Guihéry, N.; Barra, A. L.; Mallah, T. Playing with Magnetic Anisotropy in Hexacoordinated Mononuclear Ni(II) Complexes, An Interplay Between Symmetry and Geometry. *Appl. Magn. Reson.* **2020**, *51*, 1215–1231.

(11) Collison, D.; Helliwell, M.; Jones, V. M.; Mabbs, F. E.; McInnes, E. J. L.; Riedi, P. C.; Smith, G. M.; Pritchard, R. G.; Cross, W. I. Single and Double Quantum Transitions in the Multi-Frequency Continuous Wave Electron Paramagnetic Resonance (CwEPR) of Three Six Co-ordinate Nickel(II) Complexes: [Ni(EtL)$_2$(Me$_5$dien)] and [Ni(5-Methylpyrazole)$_6$]X$_2$, X = (ClO$_4$)$^-$ or (BF$_4$)$^-$. The single crystal X-ray structure at room temperature of [Ni(5-methylpyrazole)$_6$](ClO$_4$)$_2$. *J. Chem. Soc - Faraday Trans.* **1998**, *94*, 3019–3025.

(12) Neese, F. The ORCA Program System. *Wiley Interdiscip. Rev.: Comput. Mol. Sci.* **2012**, 2, 73−78.

(13) Neese, F. Software Update: The ORCA Program System—Version 5.0. *WIREs Comput. Mol. Sci.* **2022**, *12*, 1–15.

(14) Weigend, F.; Ahlrichs, R. Balanced Basis Sets of Split Valence, Triple Zeta Valence and Quadruple Zeta Valence Quality for H to Rn: Design and Assessment of Accuracy. *Phys. Chem. Chem. Phys.* **2005**, *7*, 3297–3305.

(15) Adamo, C.; Barone, V. Toward Reliable Density Functional Methods without Adjustable Parameters: The PBE0 Model. *J. Chem. Phys.* **1999**, *110*, 6158–6170.

(16) Ganyushin, D.; Neese, F. A Fully Variational Spin-Orbit Coupled Complete Active Space Self-Consistent Field Approach: Application to Electron Paramagnetic Resonance *g*-Tensors. *J. Chem. Phys* **2013**, *138*.

(17) Kollmar, C.; Sivalingam, K.; Helmi.ch-Paris, B.; Angeli, C.; Neese, F. A Perturbation-Based Super-CI Approach for the Orbital Optimization of a CASSCF Wave Function. *J. Comput. Chem.* **2019**, *40*, 1463–1470.

(18) Kollmar, C.; Sivalingam, K.; Guo, Y.; Neese, F. An Efficient Implementation of the NEVPT2 and CASPT2 Methods Avoiding Higher-Order Density Matrices. *J. Chem. Phys.* **2021**, *155*.

(19) Ganyushin, D.; Neese, F. First-Principles Calculations of Zero-Field Splitting Parameters. *J. Chem. Phys.* **2006**, *125*.

(20) Llunell, M.; Casanova, D.; Cirera, J.; Alemany, P.; Alvarez, S. SHAPE, 2.1. Universitat de Barcelona: Barecelona, Spain 2013.

(21) Alvarez, S.; Avnir, D.; Llunell, M.; Pinsky, M. Continuous Symmetry Maps and Shape Classification. The Case of Six-Coordinated Metal Compounds. *New J. Chem.* **2002**, *26*, 996–1009.

(22) Ketkaew, R.; Tantirungrotechai, Y.; Harding, P.; Chastanet, G.; Guionneau, P.; Marchivie, M.; Harding, D. J. OctaDist: A Tool for Calculating Distortion Parameters in Spin Crossover and Coordination Complexes. *Dalton Trans.* **2021**, *50*, 1086–1096.

(23) Jorgensen, C. K. Comparative Crystal Field Studies of some Ligands and the Lowest Singlet State of Paramagnetic Nickel(II) Complexes. *Acta Chemica Scandinavica*. **1955**, *9*, 1362–1377.

(24) East, N. R.; Dab, C.; Förster, C.; Heinze, K.; Reber, C. Coupled Potential Energy Surfaces Strongly Impact the Lowest-Energy Spin-Flip Transition in Six-Coordinate Nickel(II) Complexes. *Inorg. Chem.* **2023**, *62*, 9025–9034.

(25) González, E.; Rodrigue-Witchel, A.; Reber, C. Absorption Spectroscopy of Octahedral Nickel(II) Complexes: A Case Study of Interactions between Multiple Electronic Excited States. *Coord. Chem. Rev.* **2007**, *251*, 351–363.

(26) Hart, S.M.; Boeyens, J. C. A.; Hancock, R. D. Mixing of states and the determination of ligand field parameters for high-spin octahedral complexes of nickel(II). Electronic spectrum and structure of bis(1,7-diaza-4-thiaheptane)nickel(II) perchlorate. *Inorg. Chem.* **1983**, *22*, 982–986.

(27) Reedijk, J.; van Leeuwen, P. W. N. M.; Groeneveld, W. L. A Semi-empirical Energy-level Diagram for Octahedral, Nickel(II) Complexes. *Recueil des Travaux Chimiques des Pays-Bas* **1968**, *87*, 129–143.

(28) Brorson, M.; Schäffer, C. E. Orthonormal Interelectronic Repulsion Operators in the Parametrical *D*q Model. Application of the Model to Gaseous Ions. *Inorg. Chem.* **1988**, *27*, 2522–2530.

(29) Falvello, L. R.; Hitchman, M. A.; Palacio, F.; Pascual, I.; Schultz, A. J.; Stratemeier, H.; Tomás, M.; Urriolabeitia, E. P.; Young, D. M. Tunable Molecular Distortion in a Nickel Complex Coupled to a Reversible Phase Transition in the Crystalline State. *J. Am. Chem. Soc.* **1999**, *121*, 2808–2819.

(30) Knox, K.; Shulman, R. G.; Sugano, S. Covalency Effects in $KNiF_3$. II. Optical Studies. *Phys. Rev.* **1963**, *130*, 512–516.

(31) Perfetti, M. Cantilever Torque Magnetometry on Coordination Compounds: From Theory to Experiments. *Coord. Chem. Rev.* **2017**, *348*, 171–186.

(32) Diebold, A.; Hagen, K. S. Iron(II) Polyamine Chemistry: Variation of Spin State and Coordination Number in Solid State and Solution with Iron(II) Tris(2-Pyridylmethyl)Amine Complexes. *Inorg. Chem.* **1998**, *37*, 215–223.

(33) Wojnar, M. K.; Laorenza, D. W.; Schaller, R. D.; Freedman, D. E. Nickel(II) Metal Complexes as Optically Addressable Qubit Candidates. *J. Am. Chem. Soc.* **2020**, *142*, 14826–14830.

(34) Wojnar, M. K.; Kundu, K.; Kairalapova, A.; Wang, X.; Ozarowski, A.; Berkelbach, T. C.; Hill, S.; Freedman, D. E. Ligand Field Design Enables Quantum Manipulation of Spins in Ni^{2+} Complexes. *Chem. Sci.* **2024**, *15*, 1374–1383.

(35) Fataftah, M. S.; Bayliss, S. L.; Laorenza, D. W.; Wang, X.; Phelan, B. T.; Wilson, C. B.; Mintun, P. J.; Kovos, B. D.; Wasielewski, M. R.; Han, S.; Sherwin, M. S.; Awschalom, D. D.; Freedman, D. E. Trigonal Bipyramidal V^{3+} Complex as an Optically Addressable Molecular Qubit Candidate. *J. Am. Chem. Soc.* **2020**, *142*, 20400–20408

(36) Laorenza, D. W.; Mullin, K. R.; Weiss, L. R.; Bayliss, S. L.; Deb, P.; Awschalom, D. D.; Rondinelli, J. M.; Freedman, D. E. Coherent Spin-Control of $S = 1$ Vanadium and Molybdenum Complexes. *Chem. Sci.* **2024**, *15*, 14016–14026.

(37) Bayliss, S. L.; Laorenza, D. W.; Mintun, P. J.; Kovos, B. D.; Freedman, D. E.; Awschalom, D. D. Optically Addressable Molecular Spins for Quantum Information Processing. *Science* **2020**, *370*, 1309–1312.

(38) Laorenza, D. W.; Kairalapova, A.; Bayliss, S. L.; Goldzak, T.; Greene, S. M.; Weiss, L. R.; Deb, P.; Mintun, P. J.; Collins, K. A.; Awschalom, D. D.; Berkelbach, T. C.; Freedman, D. E. Tunable Cr^{4+} Molecular Color Centers. *J. Am. Chem. Soc.* **2021**, *143*, 21350–21363.
